# Discovery and Evaluation of Enantiopure 9*H*-pyrimido[4,5-*b*]indoles as Nanomolar GSK-3β Inhibitors with Improved Metabolic Stability

**DOI:** 10.3390/ijms21217823

**Published:** 2020-10-22

**Authors:** Stanislav Andreev, Tatu Pantsar, Ahmed El-Gokha, Francesco Ansideri, Mark Kudolo, Débora Bublitz Anton, Giulia Sita, Jenny Romasco, Christian Geibel, Michael Lämmerhofer, Márcia Ines Goettert, Andrea Tarozzi, Stefan A. Laufer, Pierre Koch

**Affiliations:** 1 Institute of Pharmaceutical Sciences, Department of Medicinal and Pharmaceutical Chemistry, Eberhard Karls University Tübingen, Auf der Morgenstelle 8, 72076 Tübingen, Germany; stanislav.andreev@uni-tuebingen.de (S.A.); tatu.pantsar@uni-tuebingen.de (T.P.); ahmed.abdelaleem@science.menofia.edu.eg (A.E.-G.); francesco.ansideri@uni-tuebingen.de (F.A.); mark.kudolo@uni-tuebingen.de (M.K.); stefan.laufer@uni-tuebingen.de (S.A.L.); 2 School of Pharmacy, Faculty of Health Sciences, University of Eastern Finland, P.O. Box 1627, 70211 Kuopio, Finland; 3 Chemistry Department, Faculty of Science, Menoufia University, Gamal Abdel-Nasser Street, Shebin El-Kom 32511, Egypt; 4 Cell Culture Laboratory, Postgraduate Program in Biotechnology, University of Vale do Taquari (Univates), Lajeado 95914-014, Brazil; debora.anton@univates.br (D.B.A.); marcia.goettert@univates.br (M.I.G.); 5 Department of Pharmacy and Biotechnology, Alma Mater Studiorum, University of Bologna, Via Irnerio, 48, 40126 Bologna, Italy; giulia.sita2@unibo.it; 6 Department for Life Quality Studies, Alma Mater Studiorum, University of Bologna, Corso D’Augusto, 237, 47921 Rimini, Italy; jenny.romasco@unibo.it (J.R.); andrea.tarozzi@unibo.it (A.T.); 7 Institute of Pharmaceutical Sciences, Department of Pharmaceutical (Bio-)Analysis, Eberhard Karls University Tübingen, Auf der Morgenstelle 8, 72076 Tübingen, Germany; christian.geibel@uni-tuebingen.de (C.G.); michael.laemmerhofer@uni-tuebingen.de (M.L.); 8 Department of Pharmaceutical/Medicinal Chemistry II, Institute of Pharmacy, University of Regensburg, Universitätsstraße 31, 93053 Regensburg, Germany

**Keywords:** protein kinase, kinase inhibitor, 9*H*-pyrimido[4,5-*b*]indole, glycogen synthase kinase-3β, metabolic stability

## Abstract

Glycogen synthase kinase-3β (GSK-3β) is a potential target in the field of Alzheimer’s disease drug discovery. We recently reported a new class of 9*H*-pyrimido[4,5-*b*]indole-based GSK-3β inhibitors, of which 3-(3-((7-chloro-9*H*-pyrimido[4,5-*b*]indol-4-yl)(methyl)amino)piperidin-1-yl)propanenitrile (**1**) demonstrated promising inhibitory potency. However, this compound underwent rapid degradation by human liver microsomes. Starting from **1**, we prepared a series of amide-based derivatives and studied their structure–activity relationships against GSK-3β supported by 1 µs molecular dynamics simulations. The biological potency of this series was substantially enhanced by identifying the eutomer configuration at the stereocenter. Moreover, the introduction of an amide bond proved to be an effective strategy to eliminate the metabolic hotspot. The most potent compounds, (*R*)-3-(3-((7-chloro-9*H*-pyrimido[4,5-*b*]indol-4-yl)(methyl)amino)piperidin-1-yl)-3-oxopropanenitrile (***(R)*-2**) and (*R*)-1-(3-((7-bromo-9*H*pyrimido[4,5-*b*]indol-4-yl)(methyl)amino)piperidin-1-yl)propan-1-one (***(R)*-28**), exhibited IC_50_ values of 480 nM and 360 nM, respectively, and displayed improved metabolic stability. Their favorable biological profile is complemented by minimal cytotoxicity and neuroprotective properties.

## 1. Introduction

Glycogen synthase kinase-3 (GSK-3) is a ubiquitously expressed protein existing in two highly related isoforms, GSK-3α and GSK-3β [1]. This serine/threonine kinase is involved in several signal cascades and is assumed to phosphorylate more than 30 different substrates, highlighting its multifaceted role in intracellular processes [1,2]. Under physiological conditions, complex mechanisms ensure a strict regulation and proper functionality of this highly multitasking enzyme. In turn, aberrant activity of GSK-3 is assumed to be a critical factor for the development of diverse pathologies, including diabetes, cancer, bipolar disorder, and especially Alzheimer’s disease (AD) [3]. In particular, GSK-3β is implicated in crucial mechanisms associated with AD pathology. These include tau hyperphosphorylation and Aβ generation, the two major histopathological hallmarks of the disease [4]. Therefore, GSK-3β is a potential target for novel disease-modifying AD therapeutics motivating drug discovery efforts in the field of small molecule kinase inhibitors.

**Figure 1:**
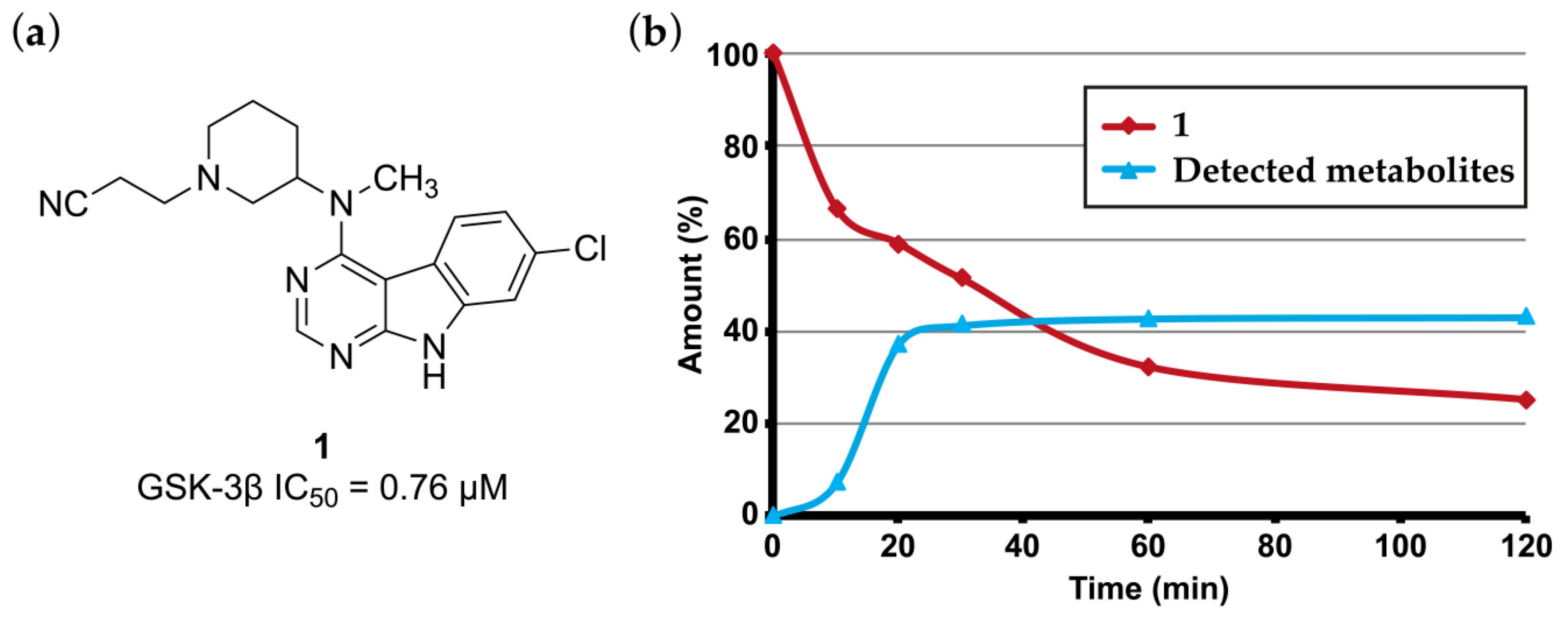
(**a**) Structure and biological activity of compound **1;** (**b**) metabolic degradation and formation of detected metabolites of **1**.

Recently, we reported on the optimization of a novel class of 7-chloro-9*H*-pyrimido[4,5-*b*]indole-based glycogen synthase kinase-3β (GSK-3β) inhibitors, including compound **1** (Figure 1a) [5]. This tertiary alicyclic amine with a promising biological activity on the target enzyme, however, suffered from poor metabolic stability when exposed to human liver microsomes (HLMs). In the HLM stability assay, inhibitor **1** underwent rapid biotransformation, resulting in a limited half-life of approximately 30 min (Figure 1b).

Tertiary alicyclic amines represent common yet metabolically vulnerable motifs in pharmaceutical agents. Their oxidative metabolization typically includes chemical modifications such as α-carbonyl introduction and ring opening as well as oxygenation or dealkylation of the nitrogen atom [6]. Correspondingly, the observed metabolic lability of **1** can be ascribed to the (2-cyanoethyl)piperidine substructure of the molecule. The mass spectrometry-based profiling of the metabolites formed from **1** in the HLM experiment indicated an extensive elimination of the cyanoethyl substituent through C–N bond cleavage. However, we found this moiety to be of utmost importance for the biochemical activity of **1** in our preliminary optimization study. This suggests that its removal would compromise the activity of this compound.

**Scheme 1:**
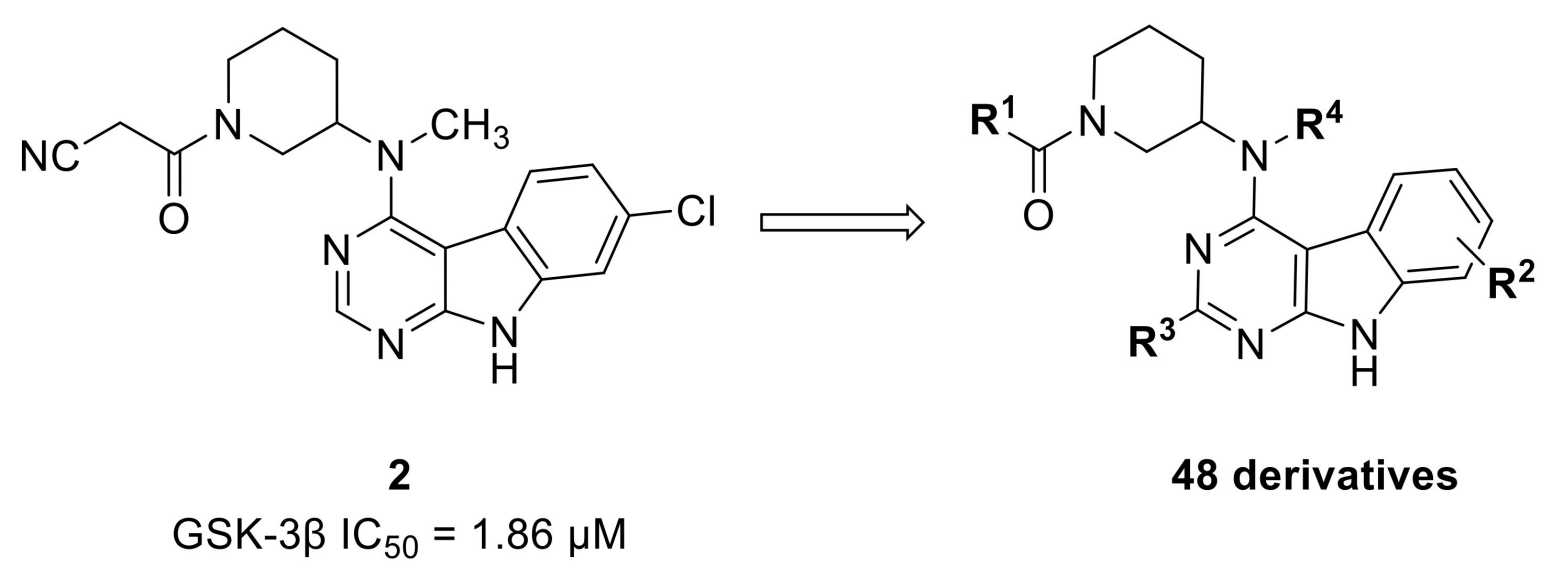
Development of novel amide-based Glycogen synthase kinase-3β (GSK-3β) inhibitors; IC_50_: halfmaximal inhibitory concentration.

Herein, we present a strategy to improve the metabolic stability of this class of 9*H*-pyrimido[4,5-*b*]indole-based GSK-3β inhibitors while maintaining the biological activity. We hypothesized that the introduction of an acyl substituent on the piperidine nitrogen atom was a suitable approach to eliminate the potential metabolic hotspot. To this end, we refocused our attention on the reported amide derivative **2**, which was 2.5-fold less active than **1** [5]. We used this compound as a template in order to design and optimize a series of novel amide-based GSK-3β inhibitors by applying a variety of structural modifications (Scheme 1). The obtained biological data established structure–activity relationships (SARs), which were substantiated by in silico approaches. The most promising candidates were assessed for their metabolic stability in the HLM experiment and further were characterized in cellular assays.

## 2. Results and Discussion

### 2.1. Biological Evaluation

In our initial attempts to optimize **2** for GSK-3β inhibition, we focused on the piperidine nitrogen substituent and evaluated its effect on the compound potency (Table 1). The application of bulky moieties including aromatic rings (**8** and **10**), an ethyl ester function (**9**), or a *tert*-butyloxy group (**6c**) led to inactive compounds. Analogs carrying substituents with longer aliphatic moieties such as butanoyl- (**11**), isovaleryl- (**12**), or cyclopropylacetyl- (**15**) were inactive or exhibited significantly higher IC_50_ values than **2**. Derivatives with shorter hydrocarbon chains, i.e., cyclopropanoyl- (**39**), acryloyl- (**40**), 3-(dimethylamino)propanoyl- (**13**), and *tert*-butanoyl- (**38**), were found to retain moderate biological activity. In agreement with this trend, a slight potency improvement was seen with the acetyl- and propanoyl-substituted compounds **14** and **16** compared to their cyanoacetyl counterpart. Perhaps surprisingly, the 3,3,3-trifluoropropanoyl derivative **17** was clearly less active than its propanoyl congener **16**. This dramatical difference in potency can be rationalized by the larger van der Waals volume of the CF_3_ group as compared to a CH_3_ group [7]. These results indicate that only short-chained substituents such as acetyl-, cyanoacetyl-, or propanoyl- are tolerated in this position.

To assess the effect of the carbonyl group functionality, we replaced it with an oxetane ring (**41**). This four-membered heterocyclic motif has been successfully applied in medicinal chemistry programs in order to improve the metabolic stability of drug candidates [8]. The resulting compound **41**, however, displayed a 4-fold higher IC_50_ value than **2**. An oxetane is considered to form weaker hydrogen bonds than an amide carbonyl group [9]. Thus, we ascertain that carbonyl group-mediated interactions are important for the binding affinity of **2**.

**Table 1:** Structures and biological activities of compounds **2**, **6c**, **8**–**17**, and **38**–**41**.

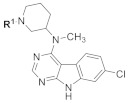					
Cpd.	R^1^	IC_50_ (µM) Mean ± SEM GSK-3β ^a^	Cpd.	R^1^	IC_50_ (µM) Mean ± SEM GSK-3β ^a^
**2**	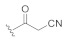	1.86 ± 0.11 ^b^	**14**	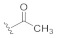	1.57 ± 0.30
**6c**	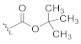	≥10	**15**	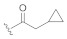	≥10
**8**	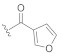	5.46 ± 1.03	**16**	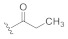	1.71 ± 0.52
**9**	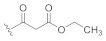	≥10	**17**	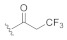	≥10
**10**	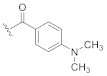	≥10	**38**	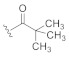	4.39 ± 0.09
**11**	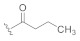	7.07 ± 1.03	**39**	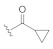	3.49 ± 0.66
**12**	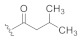	≥10	**40**	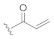	3.92 ± 0.56
**13**	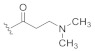	3.83 ± 0.08	**41**	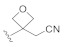	6.70 ^c^

^a^ IC_50_ values were determined in an ADP Glo™ Kinase assay [10,11] (for details, see the Supplementary Materials) and are the means of at least two independent experiments; ^b^ data taken from [5]; ^c^ *n* = 1.

The results obtained from the piperidine substituent series highlighted the suitability of both the cyanoacetyl and propanoyl substituent in this position. Based on these results, we elected to maintain both moieties for further optimization and shifted our focus to other positions of the scaffold that are amenable to modification (Table 2).

Replacement of the chlorine atom in the 7-position of the tricyclic core (R^2^ in Table 2) with different halides, i.e., fluorine (**19**), bromine (**20**), and iodine (**21**), as well as removal of this substituent (**18**) was well tolerated. In fact, the inhibitory potency within these compounds marginally increased alongside the van der Waals radii of the halogens. In contrast, the introduction of a methoxy (**22**) or CF_3_ (**23**) group in this position gave inactive derivatives, presumably due to a steric clash at the target binding site. Similarly, the addition of a methyl group in the 2-position of the 7-chloro-9*H*-pyrimido[4,5-*b*]indole (**24**) resulted in a substantial loss in potency. Within this series, the removal of the *N*-methyl group (R^4^ in Table 2) typically proved unfavorable to the activity (**45**–**50**), which prompted us to maintain this substituent.

Accordingly, the activity trend observed for the halogen series in Table 2 was maintained with the propionamides (Table 3). The only exception was the iodine derivative (**29**), which exhibited a 5-fold reduced potency compared to its bromine counterpart **28**. Unfortunately, no inhibition data could be generated for the methylated propionamide **27** due to insufficient aqueous solubility.

The relocation of the R^2^ substituent to the adjacent 6-position retained potency in the case of chlorine (**30** and **34**) and bromine (**31** and **35**) (Table 4). In contrast to compound **22**, the methoxy substituent was also tolerated in this position (**32** and **36**). However, compounds **33** and **37** carrying chlorine in the 5-position of the tricyclic scaffold suffered from a decrease in activity relative to their regioisomers **2** and **15**.

As observed in several instances [12,13], the bioactivity of enantiomers may be strikingly different. The compounds presented in this work have a stereocenter in the 3-position of the piperidine ring. We expected the conformations of the different enantiomers to be unidentical, which motivated us to examine the influence of stereochemistry on the compound activity. To this end, we prepared the enantiopure analogs ***(R)*-2**/***(S)*-2** and ***(R)*-20**/***(S)*-20** of inhibitors **2** and **20**, respectively, and determined their IC_50_ values. Within these two matched pairs, the respective *(S)*-configured enantiomers showed a dramatic loss in activity, while their *(R)*-configured stereoisomers displayed IC_50_ values in the nanomolar range. Having identified the eutomer configuration, we also prepared ***(R)*-28**, which followed the same trend and exhibited an improved IC_50_ value of 360 nM. These findings demonstrated that only the *(R)*-enantiomer displays GSK3β inhibition among these compounds.

### 2.2. Molecular Modeling

**Figure 2:**
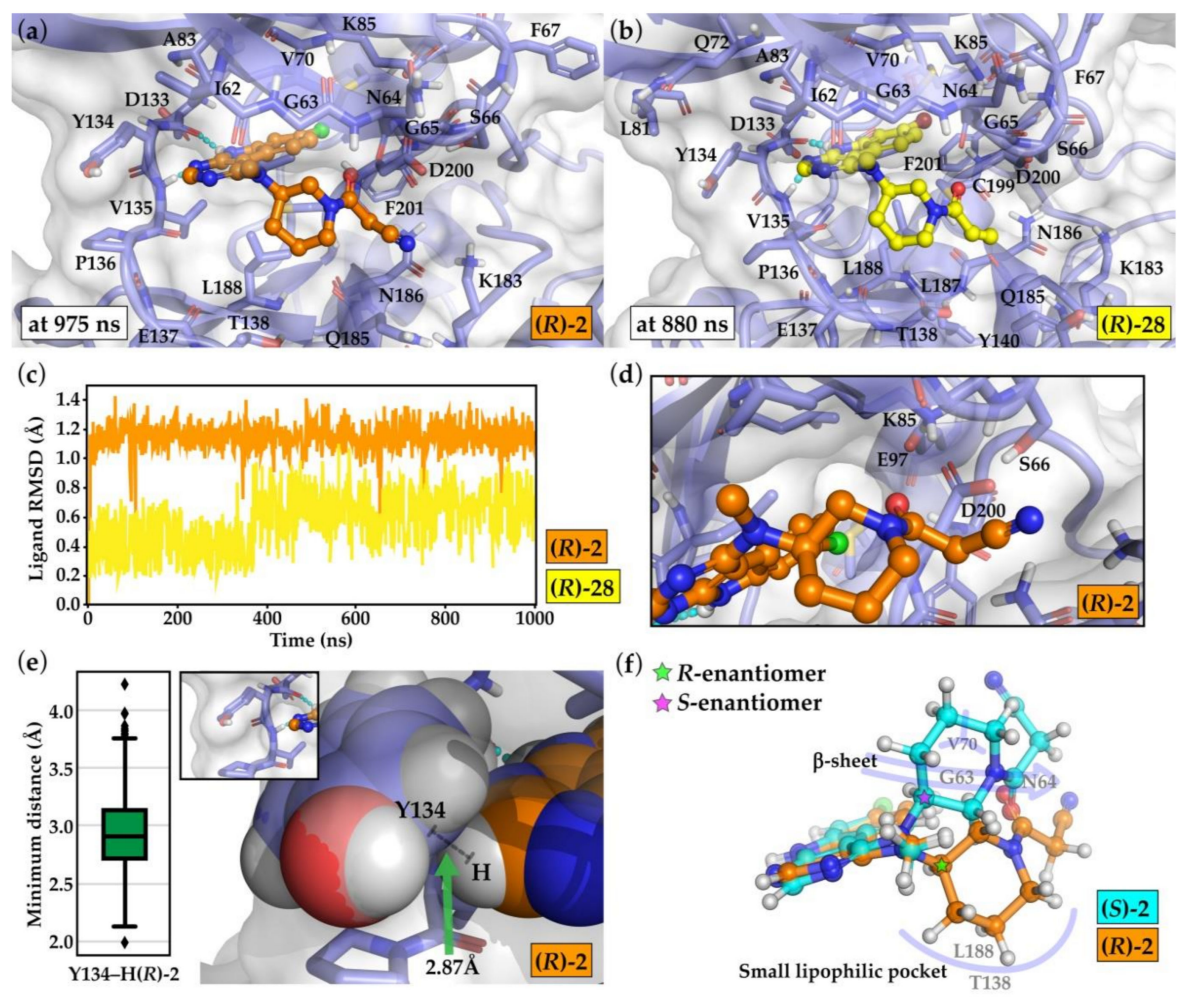
Putative binding mode of the described GSK3β inhibitors: (**a**) representative snapshot of the ***(R)*-2** binding conformation during the 1 μs molecular dynamics (MD) simulation. All residues located within 4 Å of the ligand during the simulation are shown as sticks. Hydrogen bonds are depicted with cyan dashed lines; (**b**) representative snapshot of the ***(R)*-28** binding conformation during the 1 μs MD simulation; (**c**) the ligands appear extremely stable in the shown conformation throughout the simulations as demonstrated by their root-mean-square deviation (RMSD) values; (**d**) the carbonyl oxygen points towards the polar residues Asp200, Lys85, Ser66, and Glu97 but does not form any specific direct interactions with GSK-3β; (**e**) the proton in the 2-position of the pyrimidine ring is in close proximity to the side chain of Tyr134. Here, the conformation of (**a**) is illustrated, where the distance is 2.87 Å. The ***(R)*-2** and Tyr134 atoms are shown as spheres representing their van der Waals radius; and (**f**) comparison of the preferred quantum mechanics (QM)-derived conformations of the different enantiomers of **2**. ***(S)*-2** is unable to obtain the binding conformation as observed for ***(R)*-2**. In the preferred ***(S)*-2** conformation, the piperidine ring would clash with the β-sheet and Val70 and would not occupy the small lipophilic pocket formed by Leu188 and Thr138.

To gain insight into the binding interactions of these amide-based compounds within the ATP site of GSK-3β, we performed 1 µs molecular dynamics (MD) simulations for the most potent inhibitors ***(R)*-2** and ***(R)*-28**. The observed binding modes for ***(R)*-2** and ***(R)*-28** are highly similar and provide plausible explanations for structure–activity relationships of the compound series (Figure 2a,b). Both compounds display extremely stable hydrogen bonding interactions to the hinge region residues Asp133 and Val135 (>95% frequency, Figure S1). The halogen substituent in the 7-position of the tricyclic scaffold (chlorine in ***(R)*-2** and bromine in***(R)*-28**) is pointing towards the hydrophobic region I of the kinase. Furthermore, the observed low values of ligand root-mean-square deviation (RMSD) highlight the stable binding conformation of both ligands.

The carbonyl oxygen, which points towards the polar residues Asp200, Lys85, Ser66, and Glu87, does not form any direct interactions to any residues of GSK3β throughout the simulations (Figure 2d). Nevertheless, water-mediated interactions are evident for the carbonyl group (Figure S1), which might explain the enhanced activity of **2** compared to its oxetane bioisoster **41**.

The piperidine ring of both compounds occupies a small lipophilic pocket formed by Leu188 (and Thr138) in the bottom of the binding site (Figure 2a,b). The amide substituent on the piperidine nitrogen atom is oriented towards the solvent interface with polar residues, e.g., Lys183. We postulate that this space, which does not present any suitable binding sites for accommodation, especially of bulkier lipophilic substituents, provides an explanation for the observed activity trends in Table 1.

The proton in the 2-position of the pyrimidine ring is in close proximity and in contact with the side chain of hinge residue Tyr134 (Figure 2e). This observation offers an explanation for the inactivity of 2-methyl derivative **24**, as this substitution would result in a steric clash with the side chain of Tyr134.

The remarkable activity difference among the stereoisomers can be realized via the comparison of the preferred quantum mechanics (QM)-derived conformations of ***(R)*-2** and ***(S)*-2** (Figure 2f). The superimposition of the enantiomers clearly demonstrates that a favorable configuration of the piperidine is not possible for ***(S)*-2**. This enantiomer not only would be unable to occupy the small lipophilic pocket but also would clash with the β-sheet next to the G-loop (Figure 2f).

### 2.3. Microsomal Stability

As our initial aim was to improve the metabolic stability of the compounds, we evaluated the most potent eutomers ***(R)*-2** and ***(R)*-28** in our in-house microsomal stability assay. To this end, the compounds were incubated with pooled male and female HLM for 120 min, and the compound degradation as well as the formation of metabolites was monitored by liquid chromatography-mass spectrometry (LC-MS) analysis.

**Figure 3:**
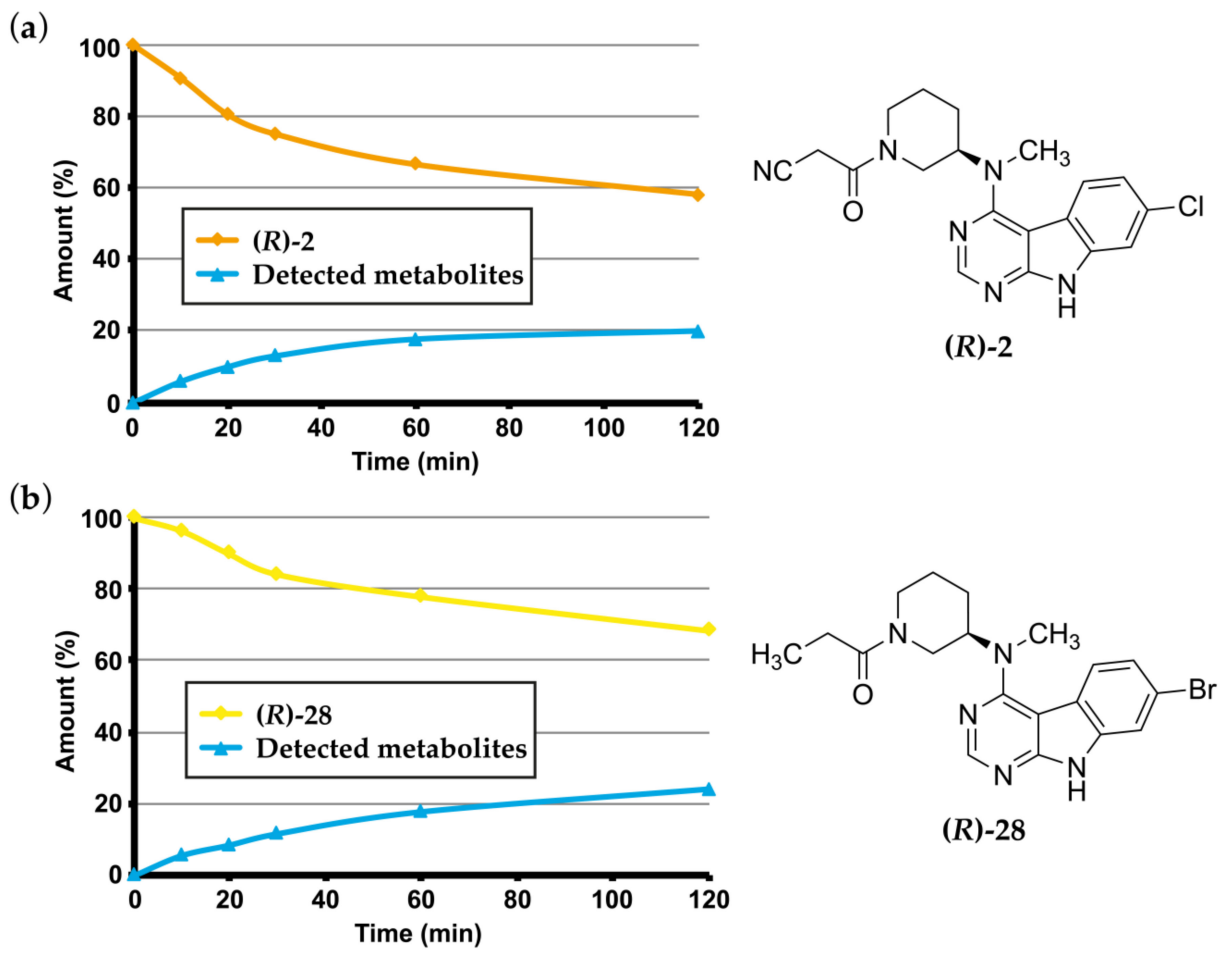
Microsomal degradation and formation of detected metabolites of (**a**) ***(R)*-2** and (**b**)***(R)*-28** during the incubation time of 120 min.

In contrast to the labile tertiary amine **1**, the enantiopure cyanoacetamide compound ***(R)*-2** displayed a favorable metabolic profile in the microsomal stability assay (Figure 3a). The metabolization of this inhibitor was characterized by a slower degradation rate and consequently a larger fraction of intact compound after the time span of 120 min. Some metabolites with an *m*/*z* ratio of 399 were detected in the LC-MS analysis and likely result from monooxygenation of the parent compound. However, no elimination of the piperidine nitrogen substituent was seen, which supports our initial hypothesis concerning stability of the amide bond.

The propanoyl analog ***(R)*-28** even showed a slightly enhanced stability compared to its cyanoacetamide counterpart. Nearly 70% of unchanged inhibitors was detected after the incubation time of 120 min (Figure 3b). We assume that this noticeable decrease in decomposition can be attributed to the lack of the metabolically vulnerable nitrile group. Similar to ***(R)*-2**, the LC-MS-based metabolite profiling indicates formation of oxygenated derivatives of ***(R)*-28** yet no cleavage of the piperidine amide bond.

### 2.4. Cell Data

To extend the biological profile of inhibitor ***(R)*-28**, we evaluated the in vitro cytotoxic potential of this compound on a variety of cell lines. These included two wild-type cell lines (human lung fibroblast cell line MRC-5 and Chinese hamster ovary cell line CHO-K1) as well as three cancer cell lines (hepatocellular carcinoma cell line HepG2, human breast adenocarcinoma cell line MCF-7, and human neuroblastoma cell line SH-SY5Y). The cells were treated with different concentrations of ***(R)*-28**, and their viability was assessed by the 3-(4,5-dimethylthiazol-2-yl)-2,5-diphenyltetrazolium bromide (MTT) colorimetric assay (for details, see Supplementary Materials). Even at the highest tested concentration of 10 µM, minimal to no cytotoxic effects were seen for ***(R)*-28** on all cell lines, demonstrating a highly favorable cytotoxicity profile (Figures S2 and S3).

These results motivated us to further characterize compound ***(R)*-28** by its ability to inhibit GSK-3β and to exert neuroprotective effects in neuronal SH-SY5Y cells (for details, see Supplementary Materials). Initially, we determined the concentrations of ***(R)*-28** not associated to neurotoxicity by the MTT assay [14]. Therefore, concentrations of 1 and 5 µM were selected for the assays in SH-SY5Y cells, which were performed according to previously described protocols [15,16,17]. At the tested concentration of 1 μM, ***(R)*-28** inhibited GSK3β activity in terms of inactive phospho-GSK3α/β (Ser21/9) increase and active phospho-GSK3α/β (Tyr279/Tyr216) decrease after 1 h of treatment in neuronal SH-SY5Y cells (Figure S4).

Next, we investigated the neuroprotective effects of ***(R)*-28** in SH-SY5Y cells against the neurotoxicity induced by neurotoxins. These included hydrogen peroxide (H_2_O_2_, 100 μM) and amyloid-β 1–42 oligomers (OAβ_1−42_, 10 μM), that mimic general oxidative stress and Alzheimer’s disease (AD), respectively. In these experiments, the concomitant treatment of 5 μM ***(R)*-28** with neurotoxins significantly decreased the neurotoxicity elicited by H_2_O_2_ but not OAβ_1−42_ (Figure S5). Still, these neuroprotective effects against the oxidative stress underline the potential usefulness of inhibitor ***(R)*-28** in the AD therapeutic area.

### 2.5. Chemistry

**Scheme 2:**
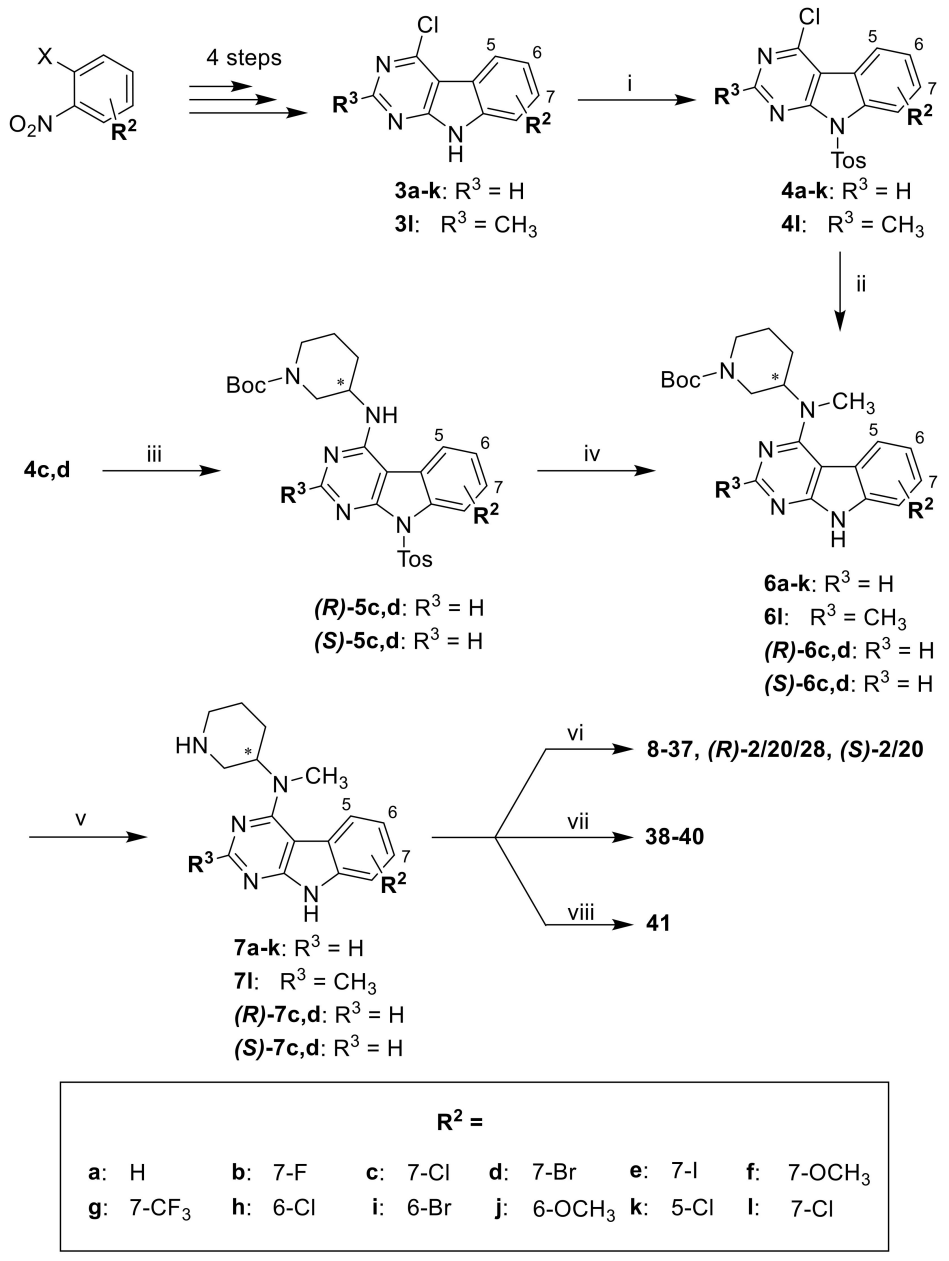
Synthetic route to final compounds **8**–**41**, ***(R)*-2/20/28**, and***(S)*-2/20** listed with their structures in Table 1, Table 2, Table 3 and Table 4. Reagents and conditions: (i) *p*-toluenesulfonyl chloride, NaH, THF, rt (82%-quant.); (ii) (1) 1-Boc-3-(methylamino)piperidine, DIPEA, DMF, 70 °C, (2) K*t*BuO or Na*t*BuO, THF or DMF, rt (43–74% over two steps); (iii) *(R)*-1-Boc-3-aminopiperidine in case of ***(R)*-5c**,**d** or *(S)*-1-Boc-3-aminopiperidine in case of ***(S)*-5c**,**d**, DIPEA, DMF, 70 °C (62–80%); (iv) (1) methyl iodide, NaH, DMF, −10 °C to rt, (2) K*t*BuO, THF, rt (24–70% over two steps); (v) TFA, DCM, rt (76%-quant.); (vi) corresponding carboxylic acid, appropriate amide coupling reagent, DIPEA or TEA, DCM, rt (22–83%); and (vii) **7c**, corresponding acid chloride, DIPEA, THF or DCM, 0 °C to rt (47–76%); (viii) **7c**, 2-(oxetan-3-yliden)acetonitrile, EtOH, 70 °C (76%). Abbreviations: THF: tetrahydrofurane; DMF: dimethylformamide; DIPEA: *N*,*N*-diisopropylethylamine; TFA: trifluoroacetic acid; DCM: dichloromethane; DCC: *N*,*N′*-dicyclohexylcarbodiimide; TEA: triethylamine; rt: room temperature.

We previously reported a synthetic route for the preparation of compound **2** [5]. This strategy demonstrated broad applicability within the herein presented study, as it provided access to the majority of final compounds with only minor alterations in the experimental protocols (Scheme 2). 4-Chloro-9*H*-pyrimido[4,5-*b*]indoles **3a**–**l** were prepared in four steps from commercially available *o*-halonitrobenzenes according to modified literature procedures (for details, see Supplementary Materials) [5,18,19,20]. These tricyclic intermediates were protected by a tosyl group on the indole nitrogen, resulting in **4a**–**l**. The aliphatic side chain was then introduced by treatment of **4a**–**l** with appropriate amines under basic conditions. In the case of **6a**–**l**, we used racemic 1-Boc-3-(methylamino)piperidine, which was prepared as described previously [5]. For the synthesis of analogs ***(R)*-5c**,**d** and ***(S)*-5c**,**d**, enantiopure 1-Boc-3-aminopiperidine building blocks were utilized. This demanded an additional methylation step, which was carried out with methyl iodide under strictly anhydrous, basic conditions [21]. Subsequent cleavage of the orthogonal protecting groups furnished precursors **7a**–**l**, ***(R)*-7c**,**d**, and ***(S)*-7c**,**d**, which were acylated on the piperidine nitrogen to access the final compounds.

Searching for a generally applicable and regioselective amide coupling procedure, various reagents including 3-(ethyliminomethylidenamino)-*N*,*N*-dimethyl-propan-1-amine (EDCI), benzotriazol-1-yl-oxytripyrrolidinophosphonium hexafluorophosphate (PyBOP), and *O*-(benzotriazol-1-yl)-*N*,*N*,*N*′,*N*′-tetramethyluronium tetrafluoroborate (TBTU) were tested. The application of EDCI suffered from a slow and inefficient conversion, while utilizing PyBOP required a laborious separation of the liberated tri(pyrrolidin-1-yl)phophine oxide. In contrast, the use of TBTU conveniently provided the final compounds in short reaction times and high purity and thus became the method of choice in the course of the project. In addition, compounds **38**, **39**, and **40** were prepared using commercially available acid chlorides applying common procedures. Compound **41** was accessible via Michael reaction of **7c** with 2-(oxetan-3-yliden)acetonitrile (synthesized from (triphenylphosphoranyliden)acetonitrile [22] and oxetan-3-one according to the literature [9]) [23].

**Scheme 3:**
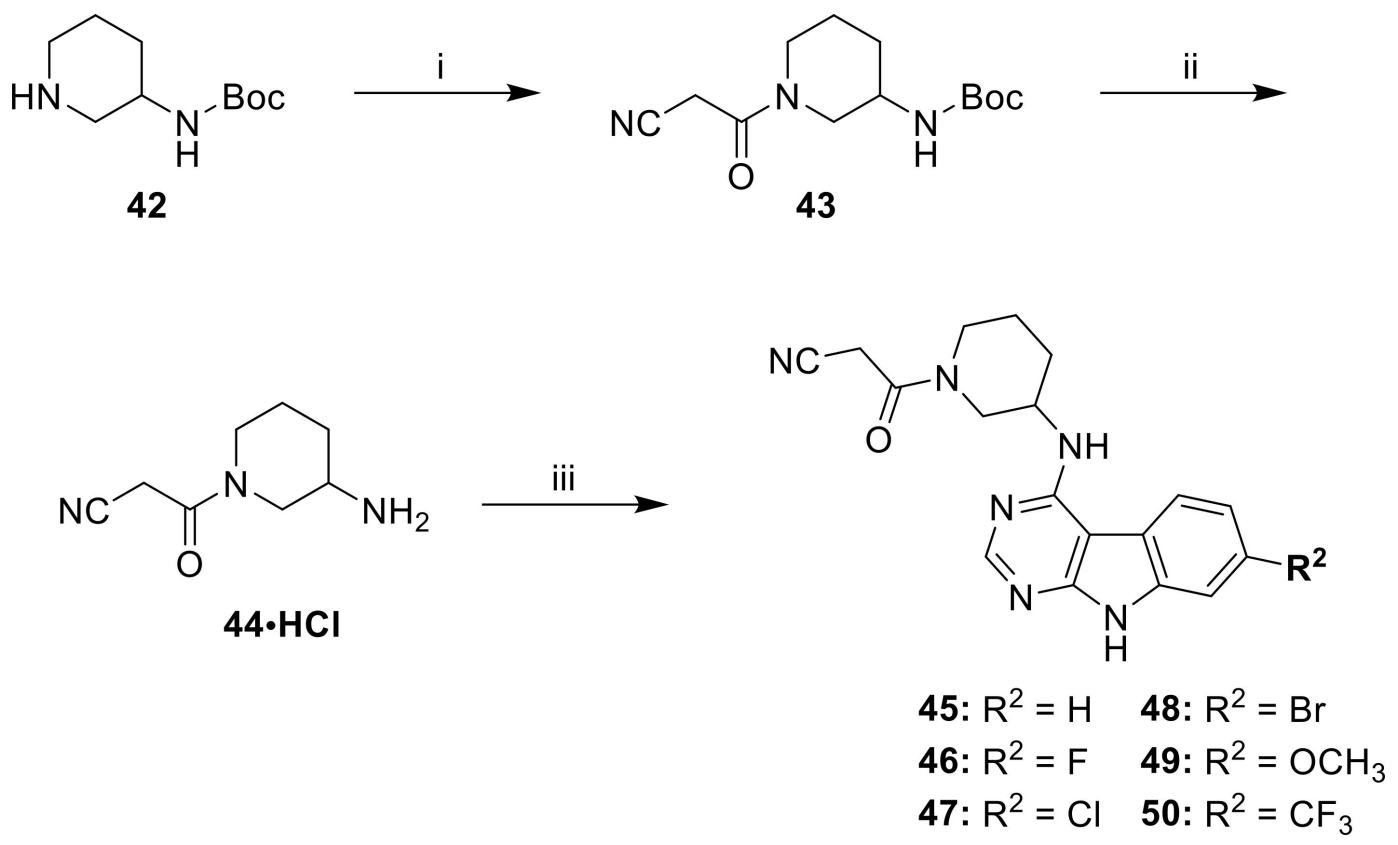
Synthetic route to final compounds **45**–**50** listed with their structures in Table 2. Reagents and conditions: (i) cyanoacetic acid, DCC, DCM, 0 °C to rt (71%); (ii) 4N HCl in dioxane, THF, rt (96%); and (iii) (1) corresponding 4-chloro-9-tosyl-9*H*-pyrimido[4,5-*b*]indole (**4a**–**d** and **4f**–**g**), DIPEA, DMF, 70 °C and (2) K*t*BuO or Na*t*BuO, THF or DMF, rt (7–53% over two steps). Abbreviations: THF: tetrahydrofurane; DMF: dimethylformamide; DIPEA: *N*,*N*-diisopropylethylamine; TFA: trifluoroacetic acid; DCM: dichloromethane; DCC: *N*,*N*′-dicyclohexylcarbodiimide; TEA: triethylamine; rt: room temperature.

For convenient access to the *N*-desmethyl analogs **45**–**50**, a 3-aminopiperidine building block with a preinstalled cyanoacetyl substituent (**44**) was synthesized (Scheme 3). Following a published protocol, 3-(Boc-amino)piperidine (**42**) was coupled with cyanoacetic acid in the presence of *N*,*N*′-dicyclohexylcarbodiimide (DCC) furnishing **43**, which was Boc-deprotected with HCl in dioxane to afford the hydrochloride salt of **44 [21]**. This precursor was reacted with the appropriate 4-chloro-9-tosyl-9*H*-pyrimido[4,5-*b*]indoles (**4a**–**d** and **4f**,**g**), and the resulting S_N_Ar products were finally detosylated to obtain **45**–**50**.

### 2.6. Conclusion

We used the moderately potent 9*H*-pyrimido[4,5-*b*]indole **2** as a lead structure to design a series of novel amide-based GSK-3β inhibitors. The newly synthesized compounds were evaluated for their biological activity on the targeted kinase in an ADP Glo^™^ assay. Compounds **2** and **28** demonstrated an optimized scaffold decoration pattern for GSK-3β inhibition. Remarkably, we observed a strong influence of the stereoconfiguration on the activity of this compound series. The *(R)*-enantiomers were found to be nanomolar inhibitors of GSK-3β, while a substantial loss in activity was seen with the *(S)*-configured counterparts. Furthermore, the most potent nanomolar inhibitors ***(R)*-2** and ***(R)*-28** and their binding modes were examined by 1 µs molecular dynamics simulations, rationalizing the SARs of the series. Most importantly, these inhibitors exhibited enhanced stability in the HLM assay as well as a minimal toxicity along with neuroprotective effects in a cellular context. The favorable properties of these compounds motivate additional studies to further assess the biological effects of this class of GSK-3β inhibitors and to finally elucidate the binding mode to the target enzyme.

## 3. Materials and Methods

### 3.1. Molecular Modelling

All in silico work was conducted with Maestro (Schrödinger Release 2019-3/4: Maestro, Schrödinger, LLC, New York, NY, USA, 2019) using OPLS3e force field [24]. Illustrations were made with PyMOL (The PyMOL Molecular Graphics System, Version 2.2.3 Schrödinger, LLC, New York, NY, USA, 2020). First, we prepared the ligands ***(R)*-2** and ***(R)*-28** with LigPrep (Schrödinger, LLC) and then optimized their conformations with the QM Conformer & Tautomer Predictor tool (Schrödinger, LLC), which utilizes Jaguar [25]. In short, this tool optimizes conformations of the compounds with increasing levels of theory, starting with semiempirical method and using density functional theory (DFT) with M06-2X/cc-pVTZ(-f) in the final step. For more detailed description of the QM Conformer & Tautomer Predictor protocol, see the supplementary information in [26]. Next, the ligand of the Protein preparation wizard [27] prepared and energy-minimized GSK-3β structure (PDB ID: 4PTC) [28] was manually replaced with the lowest energy QM-derived structure of ***(R)*-2** or ***(R)*-28**. After this, the new complex was prepared for simulations with the Protein preparation wizard [27]. For MD simulations, we used Desmond [29]. The systems were solvated in the cubic box with the minimum distance to the edges of 13 Å from the protein and neutralized with Cl^—^ions, adding a total of 0.15 M KCl salt. The water molecules were described with the TIP3P water model [30]. The production simulations of 1000 ns were run with NpT ensemble (*T* = 310 K, Nosé–Hoover method; *p* = 1.01325 bar, Martyna–Tobias–Klein method) with the default Desmond settings as described previously [5]. Before the actual production run, the default Desmond relaxation protocol was applied for both systems. For the conformation comparison of different enantiomers of **2** (in Figure 2f), ***(S)*-2** was also prepared with the QM Conformer & Tautomer Predictor tool with the same default settings as ***(R)*-2**, and they were superimposed by their hinge binding moieties.

### 3.2. Chemistry

#### 3.2.1. General Information

All solvents and reagents were purchased from commercial sources and used without further purification, if not stated otherwise. Organic solvents used for analytical chromatography were generally of HPLC grade.

High performance liquid chromatography (HPLC) was performed on a Hewlett Packard HP1090 series II HPLC system (Hewlett-Packard, Palo Alto, CA, USA) or an Agilent 1100 series HPLC system (Agilent, Santa Clara, CA, USA) equipped with a diode array detector detecting at 230 nm and 254 nm. Method A consisted of elution using mobile phase A (MeOH) and mobile phase B (aqueous 0.01 M KH_2_PO_4_ buffer, pH 2.3) in a flow of 1.5 mL/min on a Phenomenex Luna 5 µm C8(2) 100 Å RP column (150 × 4.6 mm) (Phenomenex, Torrance, CA, USA) and the gradient as follows: mobile phase A 40% to 85% during 8 min, mobile phase A 85% constant for 5 min, mobile phase A 85% to 40% during 1 min, mobile phase A 40% constant for 2 min; complete run time 16 min; injection volume 5 µL. Method B consisted of elution using the same mobile phases in a flow of 1.5 mL/min on an XBridge C18 5 µm RP column (150 × 4.6 mm) (Waters, Milford, MA, USA) and the gradient as follows: mobile phase A 45% to 85% during 10 min, mobile phase A constant for 6 min; complete run time 16 min; injection volume 10 µL. The purity of the final compounds was determined at 254 nm and was >95%.

Final compounds ***(R)*-2**/***(S)*-2**, ***(R)*-20**/***(S)*-20**, and ***(R)*-28** were evaluated for their enantiomeric purity by chiral chromatography, which was performed on an Agilent 1290 Infinity series LC system (Agilent, Santa Clara, CA, USA) consisting of a binary pump, a thermostatted column compartment, an autosampler, and a diode array detector. The system was provided with an ultralow dispersion kit (including a Max-Light ultralow dispersion cartridge flow cell with an inner volume of 0.6 µL, an ultralow dispersion needle seat, and capillaries with 0.075-mm inner diameter from autosampler to column compartment (350 mm length) and from column compartment to DAD (220 mm length)) to minimize extra column volume. The method consisted of elution using the mobile phase (64% n-heptane/36% isopropanol) in a flow of 0.4 mL/min on a Chiralpak IA-U 1.6 µm column (100 × 3.0 mm) (Daicel, Osaka/Tokyo, Japan); injection volume was 5 µL. Samples of the stereoisomers and racemates were prepared by diluting 10 mM aliquotes in dimethyl sulfoxide (DMSO) by the factor 10 with the mobile phase to a total concentration of 1 mM. The purity was determined at 254 nm. The enantiomeric excess (ee) was >98% in all cases.

Thin layer chromatography—electrospray ionization—mass spectrometry coupled analysis (TLC-ESI-MS) was performed on an Advion expression^s^ CMS coupling system (Advion, Ithaca, NY, USA). The parameters of the ESI+ mode were as follows: capillary temperature 250 °C, capillary voltage 180 V, source gas temperature 250 °C, and ESI voltage 3500 V. The parameters of the ESI− mode were as follows: capillary temperature 250 °C, capillary voltage 180 V, source gas temperature 250 °C, and ESI voltage 2500 V. The compounds were eluted from the TLC plate with MeOH.

Flash column chromatography was performed on an Interchim puriflash 430 or XS 420 (Interchim, Montluçon, France) on Grace Davison Discovery Sciences Davisil Chromatographic Silica Media LC60A (20–45 µm) (Grace Davison Discovery Sciences, MD, USA) or Interchim puriflash prepacked silica columns (SIHP-JP, 30 µm) (Interchim, Montluçon, France). For preparation of pre-columns, Merck Geduran Si60 63–200 µm silica gel (Merck, Darmstadt, Germany) was used. Mobile phases for each compound were described in the respective experimental procedure.

^1^H and ^13^C Nuclear magnetic resonance (NMR) analysis was performed on 200, 300, and 400 MHz Bruker Avance and 400 MHz Bruker Ascend spectrometers (Bruker, Billerica, MA, USA). Spectra were calibrated to residual peaks of the utilized deuterated solvents. Chemical shifts were reported in parts per million (ppm) relative to tetramethylsilane (*δ* = 0). NMR spectra of compounds with acyl substituents on the piperidine nitrogen frequently showed mixtures of amide bond rotamers resulting in complex reports. The ratio of rotamers was estimated from the respective integrals in the ^1^H-NMR spectra.

Thin layer chromatography (TLC) was performed on silica gel coated aluminum sheets (Merck TLC Silica gel F_254_, Merck, Darmstadt, Germany or Macherey-Nagel Alugram Sil G/UV_254_, Macherey-Nagel, Düren, Germany), detected under UV light (254 nm).

#### 3.2.2. General Procedures

##### (1) General Procedure A

The appropriate intermediate (**3a**,**b** and **3d**–**l**) was suspended in dry tetrahydrofurane (THF). NaH was added, and the mixture was stirred at room temperature (rt) and under N_2_ atmosphere for 15–30 min. *p*-Toluenesulfonyl chloride was added, and the mixture was stirred at rt and under N_2_ atmosphere until reaction control by TLC indicated complete consumption of the starting material. The reaction mixture was poured into ice-cold, water and saturated NH_4_Cl solution was added. The precipitate was filtered off, rinsed with fresh demineralized water, and dried over P_2_O_5_ in vacuo. The crude product was used in the next step without further purification.

##### (2) General Procedure B

The appropriate intermediate (**4a**–**l**) was suspended in dry dimethylformamide (DMF). *N*,*N*-diisopropylethylamine (DIPEA) and the appropriate Boc protected secondary amine were added, and the mixture was stirred at 70–80 °C until reaction control by HPLC indicated sufficient consumption of the starting material. After cooling down to rt, the mixture was poured into ice-cold water and saturated NH_4_Cl solution was added. The resulting precipitate was filtered off, rinsed with fresh demineralized water, and dried over P_2_O_5_ in vacuo. The crude product was used in the next step without further purification, if not stated otherwise.

##### (3) General Procedure C

A solution of the appropriate intermediate (***(R)*-5c**,**d** and ***(S)*-5c**,**d**) in dry DMF was stirred in a flame-dried Schlenk tube under Ar atmosphere and ice-cooling. NaH was added, and the mixture was left to stir for 30 min for deprotonation. Cooling was then switched to a MeOH ice bath prior to adding methyl iodide. The mixture was left to warm to 0 °C and then to rt and stirred under Ar atmosphere until HPLC indicated sufficient consumption of the starting material. The mixture was then poured into ice-cold saturated NH_4_Cl solution. The resulting precipitate was filtered off, washed with demineralized, and dried over P_2_O_5_ in vacuo. The crude product was used in the next step without further purification, if not stated otherwise.

##### (4) General Procedure D

The appropriate intermediate was dissolved in THF (dry or HPLC grade). K*t*BuO was added, and the mixture was stirred at rt and under N_2_ atmosphere until reaction control by HPLC indicated complete consumption of the starting material. Saturated NH_4_Cl solution and ethyl acetate (EtOAc) were added to the reaction mixture, and phases were separated. The aqueous layer was extracted with EtOAc (2–3×). Combined organic layers were dried over Na_2_SO_4_ and concentrated under reduced pressure. The residue was purified by flash column chromatography.

##### (5) General Procedure E

The appropriate intermediate (**6a**,**b**, **6d**–**l**, ***(R)*-6c**,**d**, and ***(S)*-6c**,**d**) was stirred in a 17% (*v*/*v*) mixture of dry dichloromethane (DCM) and trifluoroacetic acid (TFA) at rt and under N_2_ atmosphere until reaction control by HPLC indicated complete consumption of the starting material. The mixture was concentrated under reduced pressure, and saturated NaHCO_3_ solution was added to neutralize residual TFA. The mixture was then extracted repeatedly with EtOAc. MeOH was added to improve the solubility of the product in the organic layer. Combined organic layers were washed with saturated NaHCO_3_ solution (3×), dried over Na_2_SO_4_, and evaporated to dryness. The crude product was used in the next step without further purification, if not stated otherwise.

##### (6) General Procedure F

The appropriate carboxylic acid and PyBOP or TBTU were stirred in dry DCM for 15–30 min at rt and under N_2_ atmosphere. A mixture of the appropriate intermediate (**7a**–**l**, ***(R)*-7c**,**d**, and ***(S)*-7c**,**d**) and DIPEA in dry DCM was added to the activated carboxylic acids and the reaction mixture stirred at rt and under N_2_ atmosphere until reaction control by HPLC indicated complete consumption of the starting material. The mixture was diluted with DCM, washed with saturated NaHCO_3_ solution (2–3×) and saturated NH_4_Cl solution (2–3×), dried over Na_2_SO_4_, and concentrated under reduced pressure. The residue was purified by flash column chromatography.

##### (7) General Procedure G

The appropriate intermediate (**4a**–**d** and **4f**,**g**), **44·HCl**, and DIPEA were stirred in dry DMF at 70 °C overnight. After cooling down to rt, Na*t*BuO was added and the mixture was stirred at rt overnight. Saturated NH_4_Cl solution was added, and the mixture was extracted thrice with EtOAc. Combined organic layers were dried over Na_2_SO_4_ and concentrated under reduced pressure. The residue was purified by flash column chromatography.

#### 3.2.3. Detailed Procedures

##### (1) Detailed Procedures for the Preparation of Intermediates **4a**–**l**

4-Chloro-9-tosyl-9*H*-pyrimido[4,5-*b*]indole (**4a**)

The title compound was prepared from **3a** (500.0 mg, 2.45 mmol), *p*-toluenesulfonyl chloride (585.0 mg, 3.07 mmol), and NaH (147.3 mg of a 60% in mineral oil, 3.68 mmol) in dry THF (16 mL) according to general procedure A (reaction time 2 h); 753 mg of a yellow solid was yielded (82% crude yield) and used in the next step without further purification. ESI-MS: (*m*/*z*) 380.3 [M + Na]^+^, 356.3 [M − H]^−^.

4-Chloro-7-fluoro-9-tosyl-9*H*-pyrimido[4,5-*b*]indole (**4b**)

The title compound was prepared from **3b** (250.0 mg, 2.05 mmol), *p*-toluenesulfonyl chloride (268.8 mg, 1.41 mmol), and NaH (67.7 mg of a 60% dispersion in mineral oil, 1.69 mmol) in dry THF (7.5 mL) according to general procedure A (reaction time 1.5 h); 412 mg of a yellow solid was yielded (97% crude yield) and used in the next step without further purification. ESI-MS: (*m*/*z*) 398.2 [M + Na]^+^, 374.1 [M − H]^−^.

4,7-Dichloro-9-tosyl-9H-pyrimido[4,5-*b*]indole (**4c**)

The title compound was prepared from **3c** as described previously [5].

7-Bromo-4-chloro-9-tosyl-9*H*-pyrimido[4,5-*b*]indole (**4d**)

The title compound was prepared from **3d** (560.0 mg, 1.98 mmol), *p*-toluenesulfonyl chloride (472.4 mg, 2.48 mmol), and sodium hydride (118.9 mg of a 60% dispersion in mineral oil, 2.97 mmol) in dry THF (15 mL) according to general procedure A (reaction time 20 min); 834 mg of a yellow solid was yielded (96% crude yield) and used in the next step without further purification. ESI-MS: (*m*/*z*) 457.8 [M + Na]^+^, 433.9 [M − H]^−^.

4-Chloro-7-iodo-9-tosyl-9*H*-pyrimido[4,5-*b*]indole (**4e**)

The title compound was prepared from **3e** (421.0 mg, 1.35 mmol), *p*-toluenesulfonyl chloride (322.5 mg, 1.69 mmol), and sodium hydride (81.2 mg of a 60% dispersion in mineral oil, 2.03 mmol) in dry THF (10 mL) according to general procedure A (reaction time 1 h); 606 mg of a dark yellow solid was yielded (93% crude yield) and used in the next step without further purification. ESI-MS: (*m*/*z*) 506.3 [M + Na]^+^; 482.4 [M − H]^−^.

4-Chloro-7-methoxy-9-tosyl-9*H*-pyrimido[4,5-*b*]indole (**4f**)

The title compound was prepared from **3f** (710.0 mg, 3.03 mmol) and NaH (182.3 mg of a 60% dispersion in mineral oil, 4.56 mmol) in dry THF (10 mL) according to general procedure A (reaction time 30 min). *p*-Toluenesulfonyl chloride (695.1 mg, 3.65 mmol) was added as solution in THF (2 mL); 1.15 g was yielded (98% yield) and used in the next step without further purification.

4-Chloro-9-tosyl-7-(trifluoromethyl)-9*H*-pyrimido[4,5-*b*]indole (**4g**)

NaH (176.7 mg of a 60% dispersion in mineral oil, 4.42 mmol) was added portion-wise to an ice-cooled stirring suspension of **3g** (800.0 mg, 2.95 mmol) in dry THF (10 mL). The mixture was stirred under ice-cooling for 30 min. A solution of *p*-toluenesulfonyl chloride (673.8 mg, 3.53 mmol) in dry THF (2 mL) was drop-added, while the mixture was left to warm to rt and stirring continued at rt for 30 min. The mixture was poured into saturated NH_4_Cl solution (100 mL). The resulting precipitate was filtered off, washed with demineralized water, and dried over P_2_O_5_ in vacuo; 1.2 g was yielded (96% crude yield) and used in the next step without further purification. ESI-MS: (*m*/*z*) 448.0 [M + H]^+^, 424.0 [M − H]^−^.

4,6-Dichloro-9-tosyl-9*H*-pyrimido[4,5-*b*]indole (**4h**)

The title compound was prepared from **3h** (550.0 mg, 2.31 mmol), *p*-toluenesulfonyl chloride (550.2 mg, 2.89 mmol). and NaH (138.6 mg of a 60% dispersion in mineral oil, 3.47 mmol) in dry THF (18 mL) according to general procedure A (reaction time 40 min); 890 mg of a yellow solid was yielded (98% crude yield) and used in the next step without further purification. ESI-MS: (*m*/*z*) 413.9 [M + Na]^+^, 390.0 [M − H]^−^.

6-Bromo-4-chloro-9-tosyl-9*H*-pyrimido[4,5-*b*]indole (**4i**)

The title compound was prepared from **3i** (740.0 mg, 2.62 mmol), *p*-toluenesulfonyl chloride (624.2 mg, 3.27 mmol), and NaH (157.2 mg of a 60% dispersion in mineral oil, 3.93 mmol) in dry THF (33 mL) according to general procedure A (reaction time 1.5 h); 1.1 g was yielded (96% crude yield) and used in the next step without further purification. ESI-MS: (*m*/*z*) 457.7 [M + Na]^+^, 433.8 [M − H]^−^.

4-Chloro-6-methoxy-9-tosyl-9*H*-pyrimido[4,5-*b*]indole (**4j**)

The title compound was prepared from **3j** (480.0 mg, 2.05 mmol), *p*-toluenesulfonyl chloride (489.6 mg, 2.57 mmol), and sodium hydride (123.3 mg of a 60% dispersion in mineral oil, 3.08 mmol) in dry THF (16 mL) according to general procedure A in a (reaction time 30 min); 750 mg of a brown solid was yielded (94% crude yield) and used in the next step without further purification. ESI-MS: (*m*/*z*) 410.7 [M + Na]^+^, 386.7 [M − H]^−^.

4,5-Dichloro-9-tosyl-9*H*-pyrimido[4,5-*b*]indole (**4k**)

**3k** (600.0 mg, 2.52 mmol) was suspended in dry THF (20 mL), and NaH (151.2 mg of a 60% dispersion in mineral oil, 3.78 mmol) was added. The mixture was stirred at rt and under N_2_ atmosphere for 15 min. *p*-Toluenesulfonyl chloride (600.6 mg, 3.15 mmol) was added and the mixture stirred at rt and under N_2_ atmosphere for 40 min. Saturated NH_4_Cl solution (50 mL), EtOAc (30 mL), and some MeOH were added, and phases were separated. The aqueous layer was extracted with DCM (4 × 30 mL). Combined organic layers were dried over Na_2_SO_4_. Volatiles were removed under reduced pressure to yield 1.1 g of a brown solid (>100% crude yield), which contained excessive *p*-toluenesulfonyl chloride. The crude product was used in the next step without further purification. ESI-MS: (*m*/*z*) 414.7 [M + Na]^+^, 390.7 [M − H]^−^.

4,7-Dichloro-2-methyl-9-tosyl-9*H*-pyrimido[4,5-*b*]indole (**4l**)

The title compound was prepared from **3l** (470.0 mg, 1.86 mmol), *p*-toluenesulfonyl chloride (444.3 mg, 2.33 mmol), and NaH (111.9 mg of a 60% dispersion in mineral oil, 2.80 mmol) in dry THF (16 mL) according to general procedure A (reaction time 20 min); 759 mg of a beige solid was yielded (100% crude yield) and used in the next step without further purification.

##### (2) Detailed Procedures for the Preparation of Enantiopure Intermediates **(*R*)-5c**,**d** and **(*S*)-5c**,**d**

*tert*-Butyl (*R*)-3-((7-chloro-9-tosyl-9*H*-pyrimido[4,5-*b*]indol-4-yl)amino)piperidine-1-carboxylate (**(*R*)-5c**)

The title compound was prepared from **4c** (465.0 mg, 1.19 mmol), *(R)*-1-Boc-3-aminopiperidine (284.9 mg, 1.42 mmol), and DIPEA (444.3 mg, 3.44 mmol) in dry DMF (11 mL) according to general procedure B (reaction time 16 h). Purification by flash column chromatography (SiO_2_, hexane–EtOAc–MeOH 60:38:2) gave 462 mg of a pale yellow solid (69% yield). ^1^H-NMR (300 MHz, CDCl_3_) δ 8.60 (s, 1H), 8.49 (s, 1H), 8.05 (d, *J* = 7.9 Hz, 2H), 7.68 (s, 1H), 7.35 (d, *J* = 7.5 Hz, 1H), 7.25 (d, *J* = 8.9 Hz, 2H, overlap with CHCl_3_ signal), 6.44–4.91 (m, 1H), 4.55–4.31 (m, 1H), 4.23–3.66 (m, 2H), 3.54–2.97 (m, 2H), 2.46–2.01 (m, 4H), 1.97–1.79 (m, 1H), 1.75–1.30 (m, 11H); ESI-MS: (*m*/*z*) 578.0 [M + Na]^+^, 553.9 [M − H]^−^; HPLC method A: t_r_ = 10.118 min.

*tert*-Butyl (*R*)-3-((7-bromo-9-tosyl-9*H*-pyrimido[4,5-*b*]indol-4-yl)amino)piperidine-1-carboxylate (**(*R*)-5d**)

The title compound was prepared from **4d** (465.0 mg, 1.07 mmol), *(R)*-1-Boc-3-aminopiperidine (277.2 mg, 1.38 mmol), and DIPEA (412.9 mg, 3.19 mmol) in dry DMF (12 mL) according to general procedure B (reaction time 7 h). Purification by flash column chromatography (SiO_2_, hexane–EtOAc–MeOH 67:31.5:1.5) gave 400 mg of a light yellow solid (62% yield). ESI-MS: (*m*/*z*) 599.8 [M + H]^+^, 621.7 [M + Na]^+^, 597.7 [M − H]^−^; HPLC method A: t_r_ = 10.848 min.

*tert*-Butyl (*S*)-3-((7-chloro-9-tosyl-9*H*-pyrimido[4,5-*b*]indol-4-yl)amino)piperidine-1-carboxylate (**(*S*)-5c**)

The title compound was prepared from **4c** (460.0 mg, 1.17 mmol), *(S)*-1-Boc-3-aminopiperidine (305.3 mg, 1.53 mmol), and DIPEA (444.8 mg, 3.44 mmol) in dry DMF (11 mL) according to general procedure B (reaction time 9 h). Purification by flash column chromatography (SiO_2_, DCM–MeOH 97.5:2.5) gave 523 mg of a yellow solid (80% yield). ESI-MS: (*m*/*z*) 578.8 [M + Na]^+^, 554.8 [M − H]^−^; HPLC method A: t_r_ = 10.577 min.

*tert*-Butyl (*S*)-3-((7-bromo-9-tosyl-9*H*-pyrimido[4,5-*b*]indol-4-yl)amino)piperidine-1-carboxylate (**(*S*)-5d**)

The title compound was prepared from **4d** (510.0 mg, 1.17 mmol), *(S)*-1-Boc-3-aminopiperidine (315.8 mg, 1.58 mmol), and DIPEA (452.9 mg, 3.50 mmol) in dry DMF (14 mL) according to general procedure B (reaction time 6 h). Purification by flash column chromatography (SiO_2_, hexane–EtOAc–MeOH 67:31.5:1.5) gave 462 mg of a light yellow solid (66% yield). ^1^H-NMR (300 MHz, CDCl_3_) δ 8.67 (s, 1H), 8.60 (s, 1H), 8.06 (d, *J* = 8.4 Hz, 2H), 7.62 (br s, 1H), 7.49 (dd, *J* = 8.1, 1.3 Hz, 1H), 7.25 (d, 2H, overlap with CHCl_3_ signal), 6.14–5.03 (m, 1H), 4.45–4.32 (m, 1H), 4.20–3.72 (m, 2H), 3.47–2.98 (m, 2H), 2.42–2.02 (m, 4H), 1.92–1.78 (m, 1H), 1.75–1.32 (m, 11H); ESI-MS: (*m*/*z*) 599.8 [M + H]^+^, 621.8 [M + Na]^+^, 597.9 [M − H]^−^; HPLC method A: t_r_ = 10.767 min.

##### (3) Detailed Procedures for the Preparation of Intermediates **6a,b** and **6d**–**l**

*tert*-Butyl 3-(methyl(9*H*-pyrimido[4,5-*b*]indol-4-yl)amino)piperidine-1-carboxylate (**6a**)

The title compound was prepared by a two-step procedure. In the first step, **4a** (100.0 mg, 0.28 mmol), 1-Boc-3-(methylamino)piperidine (71.9 mg, 0.34 mmol), and DIPEA (108.4 mg, 0.84 mmol) were reacted in dry DMF (3 mL) according to general procedure B (reaction time 16 h) to afford 145 mg of crude *tert*-butyl 3-(methyl(9-tosyl-9*H*-pyrimido[4,5-*b*]indol-4-yl)amino)piperidine-1-carboxylate (97% crude yield), used in the second step without further purification. ESI-MS: (*m*/*z*) 558.1 [M + Na]^+^, 533.9 [M − H]^−^; HPLC method B: 10.875 min. The crude material obtained from the first step was reacted with Na*t*BuO (182.1 mg, 1.89 mmol) in dry THF (5 mL) according to general procedure D (reaction time 1 h). Purification by flash column chromatography (SiO_2_, DCM–EtOH 95:5) gave 60 mg of the title compound (58% yield). ^1^H-NMR (300 MHz, DMSO-*d_6_*) δ 12.10 (s, 1H), 8.42 (s, 1H), 7.76 (br s, 1H), 7.50 (d, *J* = 7.7 Hz, 1H), 7.43–7.35 (m, 1H), 7.25 (t, *J* = 7.4 Hz, 1H), 4.30–3.72 (m, 3H), 3.20–3.04 (m, 4H), 2.79–2.60 (m, 1H), 2.09–1.71 (m, 3H), 1.51–0.99 (m, 10H); HPLC method B: t_r_ = 8.335 min.

*tert*-Butyl 3-((7-fluoro-9*H*-pyrimido[4,5-*b*]indol-4-yl)(methyl)amino)piperidine-1-carboxylate (**6b**)

The title compound was prepared by a two-step procedure. In the first step, **4b** (420.0 mg, 1.12 mmol), 1-Boc-3-(methylamino)piperidine (287.4 mg, 1.34 mmol), and DIPEA (433.0 mg, 3.35 mmol) were reacted in dry DMF (15 mL) according to general procedure B (reaction time 16 h) to afford 549 mg of crude *tert*-butyl 3-((7-fluoro-9-tosyl-9*H*-pyrimido[4,5-*b*]indol-4-yl)(methyl)amino)piperidine-1-carboxylate as a yellow solid (89% crude yield), used in the second step without further purification. Purification of a small portion for analytical purposes was performed by flash column chromatography (SiO_2_, petroleum ether–EtOAc gradient elution from 2:1 to 1:1). ^1^H-NMR (400 MHz, CDCl_3_) δ 8.63 (s, 1H), 8.26 (dd, *J* = 10.2, 2.4 Hz, 1H), 8.10 (d, *J* = 8.4 Hz, 2H), 7.65 (s, 1H), 7.26 (d, *J* = 8.2 Hz, 2H, overlap with CHCl_3_ signal), 7.17 (td, *J* = 8.7, 2.4 Hz, 1H), 4.49–3.89 (m, 3H), 3.13 (s, 3H), 3.11–3.02 (m, 1H), 2.76–2.65 (m, 1H), 1.99–1.70 (m, 3H), 1.67–1.29 (m, 10H); ESI-MS: (*m*/*z*) 554.7 [M + H]^+^, 576.7 [M + Na]^+^, 552.7 [M − H]^−^; HPLC method A: t_r_ = 10.806 min.

The crude material obtained from the first step was reacted with K*t*BuO (780.0 mg, 6.95 mmol) in HPLC grade THF (28 mL) according to general procedure D. Purification by flash column chromatography (SiO_2_, DCM–MeOH gradient elution from 97.5:2.5 to 93:7) gave 191 mg of a beige solid (48% yield). ^1^H-NMR (400 MHz, CDCl_3_) δ 11.48 (s, 1H), 8.54 (s, 1H), 7.79–7.69 (m, 1H), 7.24 (dd, *J* = 8.9, 2.3 Hz, 1H), 7.05 (td, *J* = 9.1, 2.4 Hz, 1H), 4.59–3.98 (m, 3H), 3.27 (s, 3H), 3.12–3.02 (m, 1H), 2.77–2.60 (m, 1H), 2.08–1.77 (m, 3H), 1.69–1.54 (m, 1H), 1.43 (s, 9H); ESI-MS: (*m*/*z*) 422.5 [M + Na]^+^, 398.5 [M − H]^−^; HPLC method A: t_r_ = 9.140 min.

*tert*-Butyl 3-((7-bromo-9*H*-pyrimido[4,5-*b*]indol-4-yl)(methyl)amino)piperidine-1-carboxylate (**6d**)

The title compound was prepared by a two-step procedure. In the first step, **4d** (752.0 mg, 1.73 mmol), 1-Boc-3-(methylamino)piperidine (479.4 mg, 2.23 mmol), and DIPEA (667.4 mg, 5.17 mmol) were reacted in dry DMF (22 mL) according to general procedure B (reaction time 6 h) to afford 1.2 g of crude *tert*-butyl 3-((7-bromo-9-tosyl-9*H*-pyrimido[4,5-*b*]indol-4-yl)(methyl)amino)piperidine-1-carboxylate as a yellow solid (>100% crude yield), used in the second step without further purification. A small portion was purified for analytical purposes by flash column purification (SiO_2_; DCM–EtOH 97.5:2.5). ^1^H-NMR (300 MHz, CDCl_3_) δ 8.75–8.55 (m, 2H), 8.17–8.03 (m, 2H), 7.61–7.46 (m, 2H), 7.31–7.21 (m, 2H, overlap with CHCl_3_ signal), 4.46–3.93 (m, 3H), 3.20–2.95 (m, 4H), 2.75–2.61 (m, 1H), 2.37 (s, 3H), 2.00–1.71 (m, 3H), 1.62–1.32 (m, 10H); ^13^C NMR (50 MHz, CDCl_3_) δ 160.5, 157.2, 154.8, 154.3, 145.8, 136.3, 135.4, 129.8, 128.2, 127.3, 123.7, 120.7, 120.5, 117.5, 101.2, 80.0, 55.6, 46.7, 44.0 (br), 33.7 (br), 28.5, 28.0, 24.8, 21.8; ESI-MS: (*m*/*z*) 636.1 [M + Na]^+^, 612.2 [M − H]^−^; HPLC method *A*: t_r_ = 11.296 min. The crude material obtained from the first step was reacted with K*t*BuO (1.4 g, 12.05 mmol) in dry THF (50 mL) according to general procedure D (reaction time 1 h). Purification by flash column chromatography (SiO_2_, DCM:MeOH gradient elution from 97.5:2.5 to 93:7) gave 488 mg of a light brown solid (62% yield). ^1^H-NMR (300 MHz, CDCl_3_) δ 11.46 (br s, 1H), 8.56 (s, 1H), 7.71–7.62 (m, 2H), 7.40 (dd, *J* = 8.6, 1.8 Hz, 1H), 4.52–4.01 (m, 3H), 3.27 (s, 3H), 3.13–3.01 (m, 1H), 2.77–2.62 (m, 1H), 2.09–1.77 (m, 3H), 1.72–1.56 (m, 1H), 1.43 (s, 9H); ESI-MS: (*m*/*z*) 481.9 [M + Na]^+^, 458.0 [M − H]^−^; HPLC method A: t_r_ = 9.546 min.

*tert*-Butyl 3-((7-iodo-9*H*-pyrimido[4,5-*b*]indol-4-yl)(methyl)amino)piperidine-1-carboxylate (**6e**)

The title compound was prepared by a two-step procedure. In the first step, **4e** (590.0 mg, 1.22 mmol), 1-Boc-3-(methylamino)piperidine (339.8 mg, 1.59 mmol), and DIPEA (473.0 mg, 3.66 mmol) were reacted in dry DMF (16 mL) according to general procedure B. Dissident from the general procedure, the mixture was stirred at 60 °C for 2 h; 779 mg of crude *tert*-butyl 3-((7-iodo-9-tosyl-9*H*-pyrimido[4,5-*b*]indol-4-yl)(methyl)amino)piperidine-1-carboxylate as a beige solid (97% crude yield) was used in the second step without further purification. Purification of a small portion for analytical purposes was performed by flash column chromatography (SiO_2_, petroleum ether–EtOAc gradient elution from 3:2 to 1:1). ^1^H-NMR (300 MHz, CDCl_3_) δ 8.86 (d, *J* = 1.4 Hz, 1H), 8.62 (s, 1H), 8.09 (d, *J* = 8.4 Hz, 2H), 7.74 (dd, *J* = 8.4, 1.4 Hz, 1H), 7.41 (d, *J* = 7.9 Hz, 1H), 7.27 (d, *J* = 8.1 Hz, 2H; overlap with CHCl_3_ signal), 4.47–3.90 (m, 3H), 3.11 (s, 3H), 3.08–2.98 (m, 1H), 2.77–2.60 (m, 1H), 2.37 (s, 3H), 2.01–1.71 (m, 3H), 1.64–1.29 (m, 10H); ^13^C NMR (50 MHz, CDCl_3_) δ 160.5, 157.0, 154.8, 154.4, 145.8, 136.4, 135.4, 133.2, 129.8, 128.2, 124.0, 123.1, 121.3, 101.2, 91.3, 80.0, 55.6 (br), 46.7, 44.1 (br), 33.7 (br), 28.5, 28.1, 24.8, 21.8; ESI-MS: (*m*/*z*) 684.7 [M + Na]^+^, 660.8 [M − H]^−^; HPLC method A: t_r_ = 10.993 min.

The crude material obtained from the first step was reacted with K*t*BuO (807.4 mg, 7.2 mmol) in dry THF (30 mL) according to general procedure D (reaction time 1 h). Purification twice by flash column chromatography (SiO_2_, DCM–MeOH 95:5 and SiO_2_, DCM–MeOH gradient elution from 97.5:2.5 to 93:7) gave 292 mg of a beige solid (56% yield). ^1^H-NMR (300 MHz, CDCl_3_) δ 11.30 (s, 1H), 8.52 (s, 1H), 7.92 (d, *J* = 1.1 Hz, 1H), 7.61 (dd, *J* = 8.5, 1.4 Hz, 1H), 7.53 (d, *J* = 8.5 Hz, 1H), 4.55–4.04 (m, 3H), 3.29 (s, 3H), 3.11–2.98 (m, 1H), 2.78–2.61 (m, 1H), 2.10–1.76 (m, 3H), 1.74–1.30 (m, 10H); ESI-MS: (*m*/*z*) 530.6 [M + Na]^+^, 506.6 [M − H]^−^; HPLC method A: t_r_ = 9.541 min.

*tert*-Butyl 3-((7-methoxy-9*H*-pyrimido[4,5-*b*]indol-4-yl)(methyl)amino)piperidine-1-carboxylate (**6f**)

**4f** (250.0 mg, 0.64 mmol), 1-Boc-3-(methylamino)piperidine (165.8, 0.77 mmol), and DIPEA (249.5 mg, 1.93 mmol) were stirred in dry DMF (10 mL) at 70 °C overnight. After cooling down to rt, Na*t*BuO (433.6 mg, 4.51 mmol) was added and stirring continued at rt for 1 h. Saturated NH_4_Cl solution (150 mL) was added. The resulting precipitate was filtered off, washed with water, and dried over P_2_O_5_ in vacuo. Purification by flash column chromatography (SiO_2_, DCM–EtOH 97:3) gave 172 mg (64% yield). ^1^H-NMR (300 MHz, DMSO-*d_6_*) δ 12.00 (s, 1H), 8.37 (s, 1H), 7.64 (br s, 1H), 6.98 (d, *J* = 2.3 Hz, 1H), 6.85 (dd, *J* = 8.7, 1.6 Hz, 1H), 4.24–3.80 (m, 6H), 3.19–3.03 (m, 4H), 2.79–2.60 (m, 1H), 2.05–1.70 (m, 3H), 1.47–1.02 (m, 10H); ESI-MS: (*m*/*z*) 412.0 [M + H]^+^, 433.9 [M + Na]^+^, 410.1 [M − H]^−^; HPLC method B: t_r_ = 8.342 min.

*tert*-Butyl 3-(methyl(7-(trifluoromethyl)-9*H*-pyrimido[4,5-*b*]indol-4-yl)amino)piperidine-1-carboxylate (**6g**)

**4g** (600.0 mg, 1.41 mmol), 1-Boc-3-(methylamino)piperidine (362.4 mg, 1.69 mmol), and DIPEA (546.7 mg, 4.23 mmol) were stirred in dry DMF (20 mL) at 70 °C overnight. After cooling down to rt, Na*t*BuO (947.9 mg, 9.86 mmol) was added and stirring continued at rt for 1 h. Saturated NH_4_Cl solution (200 mL) was added. The resulting precipitate was filtered off, washed with water, and dried over P_2_O_5_ in vacuo. Purification by flash column chromatography (SiO_2_, DCM–EtOH 97:3) gave 280 mg (44% yield). ^1^H-NMR (300 MHz, DMSO-*d_6_*) δ 12.48 (s, 1H), 8.47 (s, 1H), 7.97 (br s, 1H), 7.75 (s, 1H), 7.54 (d, *J* = 8.3 Hz, 1H), 4.39–3.83 (m, 3H), 3.21 (s, 3H), 3.18–3.06 (m, 1H), 2.81–2.61 (m, 1H), 2.12–1.71 (m, 3H), 1.53–1.01 (m, 10H); HPLC method B: t_r_ = 10.213 min.

*tert*-butyl 3-((6-chloro-9*H*-pyrimido[4,5-*b*]indol-4-yl)(methyl)amino)piperidine-1-carboxylate (**6h**)

The title compound was prepared by a two-step procedure. In the first step, **4h** (450.0 mg, 1.15 mmol), 1-Boc-3-(methylamino)piperidine (368.8 mg, 1.72 mmol), and DIPEA (444.7 mg, 3.44 mmol) were reacted in dry DMF (17 mL) according to general procedure B (reaction time 14 h) to afford 682 mg of crude *tert*-butyl 3-((6-chloro-9-tosyl-9*H*-pyrimido[4,5-*b*]indol-4-yl)(methyl)amino)piperidine-1-carboxylate as a beige solid (>100% crude yield), used in the second step without further purification. Purification of a small portion for analytical purposes was performed by flash column chromatography (SiO_2_, petroleum ether–EtOAc gradient elution from 65:35 to 1:1). ^1^H-NMR (300 MHz, CDCl_3_) δ 8.61 (s, 1H), 8.43 (d, *J* = 9.0 Hz, 1H), 8.05 (d, *J* = 8.4 Hz, 2H), 7.58 (d, *J* = 2.0 Hz, 1H), 7.46 (dd, *J* = 9.0, 2.1 Hz, 1H), 7.24 (d, *J* = 9.2 Hz, 2H; overlapping with CHCl_3_ signal), 4.30–3.94 (m, 3H), 3.13 (s, 3H), 3.07–2.94 (m, 1H), 2.75–2.61 (m, 1H), 2.35 (s, 3H), 2.16–2.04 (m, 1H), 1.98–1.78 (m, 2H), 1.74–1.55 (m, 1H), 1.38 (s, 9H); ESI-MS: (*m*/*z*) 592.0 [M + Na]^+^, 568.1 [M − H]^−^; HPLC method A: t_r_ = 10.903 min.

The crude material obtained from the first step was reacted with K*t*BuO (774.5 mg, 6.91 mmol) in HPLC grade THF (29 mL) according to general procedure D (reaction time 2.5 h). Purification by flash column chromatography (SiO_2_, DCM–MeOH gradient elution from 97.5:2.5 to 93:7) gave 252 mg of a yellow solid (61% yield). ^1^H-NMR (300 MHz, CDCl_3_) δ 11.46 (br s, 1H), 8.54 (s, 1H), 7.73 (d, *J* = 1.6 Hz, 1H), 7.45 (d, *J* = 8.5 Hz, 1H), 7.38 (dd, *J* = 8.6, 1.8 Hz, 1H), 4.54–4.40 (m, 1H), 4.38–4.01 (m, 2H), 3.30 (s, 3H), 3.10–2.97 (m, 1H), 2.77–2.62 (m, 1H), 2.22–2.11 (m, 1H), 2.03–1.84 (m, 2H), 1.81–1.63 (m, 1H), 1.40 (s, 9H); ESI-MS: (*m*/*z*) 416.1 [M + H]^+^, 438.1 [M + Na]^+^, 414.1 [M − H]^−^; HPLC method A: t_r_ = 9.385 min.

*tert*-Butyl 3-((6-bromo-9*H*-pyrimido[4,5-*b*]indol-4-yl)(methyl)amino)piperidine-1-carboxylate (**6i**)

The title compound was prepared by a two-step procedure. In the first step, **4i** (650 mg, 1.49 mmol), 1-Boc-3-(methylamino)piperidine (415.1 mg, 1.94 mmol), and DIPEA (576.5 mg, 4.46 mmol) were reacted in dry DMF (20 mL) according to procedure B (reaction time 5 h) to yield 871 mg of crude *tert*-butyl 3-((6-bromo-9-tosyl-9*H*-pyrimido[4,5-*b*]indol-4-yl)(methyl)amino)piperidine-1-carboxylate as a dark yellow solid (95% crude yield), used in the second step without further purification. Purification of a small portion for analytical purposes was performed by flash column chromatography (SiO_2_, petroleum ether–EtOAc gradient elution from 2:1 to 1:1). ^1^H-NMR (300 MHz, CDCl_3_) δ 8.61 (s, 1H), 8.39 (d, *J* = 9.0 Hz, 1H), 8.05 (d, *J* = 8.3 Hz, 2H), 7.73 (d, *J* = 1.9 Hz, 1H), 7.60 (dd, *J* = 9.0, 1.9 Hz, 1H), 7.25 (d, *J* = 8.4 Hz, 2H, overlap with CHCl_3_ signal), 4.30–3.96 (m, 3H), 3.13 (s, 3H), 3.06–2.93 (m, 1H), 2.75–2.60 (m, 1H), 2.36 (s, 3H), 2.16–2.07 (m, 1H), 1.98–1.80 (m, 2H), 1.75–1.58 (m, 1H), 1.37 (s, 9H); ^13^C NMR (50 MHz, CDCl_3_) δ 160.6, 157.5, 155.0, 154.6, 145.7, 135.4, 134.4, 129.8, 129.4, 128.0, 125.3, 123.7, 117.3, 115.8, 100.5, 79.9, 55.2, 46.4 (br), 44.0 (br), 34.3, 28.4, 28.1, 24.8, 21.8; ESI-MS: (*m*/*z*) 614.2 [M + H]^+^, 636.1 [M + Na]^+^, 612.2 [M − H]^−^; HPLC method A: t_r_ = 11.194 min.

The crude material obtained from the first step was reacted with K*t*BuO (976.5 mg, 8.70 mmol) in dry THF (36 mL) according to general procedure D (reaction time 1 h). Purification by flash column chromatography (SiO_2_, DCM–MeOH gradient elution from 97.5:2.5 to 93:7) gave 364 mg of a beige solid (64% yield). ^1^H-NMR (300 MHz, CDCl_3_) δ 11.26 (br s, 1H), 8.53 (s, 1H), 7.87 (d, *J* = 0.8 Hz, 1H), 7.52 (dd, *J* = 8.5, 1.6 Hz, 1H), 7.40 (d, *J* = 8.5 Hz, 1H), 4.54–4.40 (m, 1H), 4.35–4.03 (m, 2H), 3.30 (s, 3H), 3.09–2.96 (m, 1H), 2.77–2.63 (m, 1H), 2.24–2.13 (m, 1H), 2.04–1.67 (m, 3H), 1.40 (s, 9H); ^13^C NMR (50 MHz, CDCl_3_) δ 160.2, 157.1, 155.1, 152.8, 135.5, 127.8, 125.4, 121.9, 113.7, 112.8, 98.0, 79.9, 54.8, 46.6 (br), 44.1 (br), 33.8, 28.5, 28.3, 24.9; ESI-MS: (*m*/*z*) 482.3 [M + Na]^+^, 458.3 [M − H]^−^; HPLC method A: t_r_ = 9.550 min.

*tert*-Butyl 3-((6-methoxy-9*H*-pyrimido[4,5-*b*]indol-4-yl)(methyl)amino)piperidine-1-carboxylate (**6j**)

The title compound was prepared by a two-step procedure. In the first step, **4j** (730.0 mg, 1.88 mmol), 1-Boc-3-(methylamino)piperidine (524.4 mg, 2.45 mmol), and DIPEA (729.8 mg, 5.65 mmol) were reacted in dry DMF (22 mL) according to general procedure B (reaction time 6.5 h) to afford 975 mg of crude *tert*-butyl 3-((6-methoxy-9-tosyl-9*H*-pyrimido[4,5-*b*]indol-4-yl)(methyl)amino)piperidine-1-carboxylate as a beige solid (92% crude yield), used in the next step without further purification. Purification of a small portion for analytical purposes was performed by flash column chromatography (SiO_2_). ^1^H-NMR (400 MHz, CDCl_3_) δ 8.63 (s, 1H), 8.40 (d, *J* = 9.0 Hz, 1H), 8.03 (d, *J* = 7.8 Hz, 2H), 7.22 (d, *J* = 7.9 Hz, 2H), 7.16–7.07 (m, 2H), 4.24–3.98 (m, 3H), 3.89 (s, 3H), 3.14 (s, 3H), 3.08–2.99 (m, 1H), 2.75–2.64 (m, 1H), 2.34 (s, 3H), 2.10–2.02 (m, 1H), 1.95–1.76 (m, 2H), 1.69–1.57 (m, 1H), 1.45–1.22 (m, 9H); ESI-MS: (*m*/*z*) 588.5 [M + Na]^+^, 564.6 [M − H]^−^; HPLC method A: t_r_ = 10.013 min.

The crude material obtained from the first step was reacted with K*t*BuO (1180.2 mg, 10.52 mmol) in dry THF (45 mL) according to general procedure D (reaction time 30 min). Purification by flash column chromatography (SiO_2_, DCM–MeOH gradient elution from 97.5:2.5 to 93:7) gave 369 mg of a beige solid (60% yield). ^1^H-NMR (400 MHz, DMSO-*d_6_*) δ 11.95 (s, 1H), 8.40 (s, 1H), 7.41 (d, *J* = 8.7 Hz, 1H), 7.19 (s, 1H), 7.06 (dd, *J* = 8.8, 2.4 Hz, 1H), 4.25–4.15 (m, 1H), 4.06–3.57 (m, 5H), 3.15 (s, 3H), 3.11–3.01 (m, 1H), 2.80–2.61 (m, 1H), 2.20–1.89 (m, 2H), 1.87–1.77 (m, 1H), 1.55–1.43 (m, 1H), 1.39–0.91 (m, 9H); ESI-MS: (*m*/*z*) 412.4 [M + H]^+^, 434.5 [M + Na]^+^, 410.4 [M − H]^−^; HPLC method A: t_r_ = 8.549 min.

*tert*-Butyl 3-((5-chloro-9*H*-pyrimido[4,5-*b*]indol-4-yl)(methyl)amino)piperidine-1-carboxylate (**6k**)

The title compound was prepared by a two-step procedure. In the first step, **4k** (675.0 mg, 1.72 mmol), 1-Boc-3-(methylamino)piperidine (516.3 mg, 2.41 mmol), and DIPEA (667.3 mg, 5.16 mmol) were reacted in dry DMF (25 mL) according to general procedure B. Purification of a small portion for analytical purposes was performed by flash column chromatography (SiO_2_; DCM–MeOH gradient elution from 96.5:3.5 to 92.5:7.5). ^1^H-NMR (300 MHz, CDCl_3_) δ 8.51 (s, 1H), 8.38 (d, *J* = 7.8 Hz, 1H), 8.08 (d, *J* = 6.6 Hz, 2H), 7.45–7.30 (m, 2H), 7.25 (d, 2H, overlap with CHCl_3_ signal), 4.60–3.88 (m, 3H), 3.25–2.55 (m, 5H), 2.36 (s, 3H), 2.19–1.62 (m, 4H), 1.42 (s, 9H); ESI-MS: (*m*/*z*) 592.1 [M + Na]^+^, 568.1 [M − H]^−^; HPLC method A: 10.604 min. The crude material obtained from the first step was reacted with K*t*BuO (1.2 g, 10.71 mmol) in HPLC grade THF (45 mL) according to general procedure D (reaction time 2 h). Purification by flash column chromatography (SiO_2_, DCM–MeOH gradient elution from 96.5:3.5 to 92.5:7.5) gave 379 mg of a beige solid (53% yield over two steps). ^1^H-NMR (400 MHz, DMSO-*d_6_*) δ 12.35 (s, 1H), 8.41 (s, 1H), 7.44 (d, *J* = 7.5 Hz, 1H), 7.38 (t, *J* = 7.8 Hz, 1H), 7.28 (d, *J* = 7.5 Hz, 1H), 4.35–3.65 (m, 3H), 3.09–2.87 (m, 4H), 2.84–2.63 (m, 1H), 2.18–1.66 (m, 3H), 1.50–1.09 (m, 10H); ESI-MS: (*m*/*z*) 438.1 [M + Na]^+^, 414.1 [M − H]^−^; HPLC method A: t_r_ = 9.317 min.

*tert*-Butyl 3-((7-chloro-2-methyl-9*H*-pyrimido[4,5-*b*]indol-4-yl)(methyl)amino)piperidine-1-carboxylate (**6l**)

The title compound was prepared by a two-step procedure. In the first step, **4l** (535.0 mg, 1.32 mmol), 1-Boc-3-(methylamino)piperidine (423.3 mg, 1.98 mmol), and DIPEA (510.6 mg, 3.95 mmol) were reacted in dry DMF (20 mL) according to general procedure B (reaction time 6.5 h) to afford 750 mg of crude *tert*-butyl-3-((7-chloro-2-methyl-9-tosyl-9*H*-pyrimido[4,5-*b*]indol-4-yl)(methyl)amino)piperidine-1-carboxylate as a beige solid (98% crude yield), used in the next step without further purification. Purification of a small portion for analytical purposes was performed by flash column chromatography (SiO_2_, petroleum ether–EtOAc gradient elution from 7:3 to 3:7). ^1^H-NMR (300 MHz, CDCl_3_) δ 8.52 (d, *J* = 1.8 Hz, 1H), 8.12 (d, *J* = 8.3 Hz, 2H), 7.55 (d, *J* = 8.2 Hz, 1H), 7.35 (dd, *J* = 8.5, 1.9 Hz, 1H), 7.26 (d, *J* = 8.1 Hz, 2H, overlap with CHCl_3_ signal), 4.45–3.91 (m, 3H), 3.15–2.96 (m, 4H), 2.75–2.59 (m, 4H), 2.37 (s, 3H), 1.98–1.70 (m, 3H), 1.64–1.31 (m, 10H); ESI-MS: (*m*/*z*) 606.5 [M + Na]^+^, 582.5 [M − H]^−^; HPLC method A: t_r_ = 12.593 min.

The crude material obtained from the first step was reacted with K*t*BuO (874.0 mg, 7.79 mmol) in dry THF (32 mL) according to general procedure D (reaction time 2.5 h). Purification by flash column chromatography (SiO_2_, DCM–MeOH gradient elution from 97.5:2.5 to 93:7) gave 358 mg of a light yellow solid (75% yield). ^1^H-NMR (400 MHz, DMSO-*d_6_*) δ 12.03 (s, 1H), 7.78–7.60 (m, 1H), 7.45 (d, *J* = 1.8 Hz, 1H), 7.26–7.13 (m, 1H), 4.30–3.78 (m, 3H), 3.12 (s, 3H), 3.09–3.01 (m, 1H), 2.80–2.57 (m, 1H), 2.50 (s, 3H, overlap with DMSO-*d_5_* signal), 2.08–1.68 (m, 3H), 1.54–0.99 (m, 10H); ^13^C NMR (101 MHz, DMSO-*d_6_*) δ 162.5, 159.6, 158.4, 153.9, 137.4, 128.8, 123.3, 120.2, 118.6, 110.8, 95.2, 78.7, 54.5, 46.0 (br), 43.3 (br), 32.5, 27.8, 27.4, 25.8, 24.6; ESI-MS: (*m*/*z*) 430.5 [M + H]^+^, 452.5 [M + Na]^+^; 428.6 [M − H]^−^; HPLC method A: t_r_ = 10.332 min.

##### (4) Detailed Procedures for the Preparation of Enantiopure Intermediates ***(R)*-6c,d** and ***(S)*-6c,d**

*tert*-Butyl (*R*)-3-((7-chloro-9*H*-pyrimido[4,5-*b*]indol-4-yl)(methyl)amino)piperidine-1-carboxylate (***(R)*-6c**)

The title compound was prepared by a two-step procedure. In the first step, ***(R)*-5c** (350.0 mg, 0.63 mmol), methyl iodide (134.0 mg, 0.94 mmol), and NaH (37.7 mg of a 60% dispersion in mineral oil, 0.94 mmol) were reacted in dry DMF (15 mL) according to general procedure C (reaction time of 3 h) to afford 346 mg of crude *tert*-butyl *(R)*-3-((7-chloro-9-tosyl-9*H*-pyrimido[4,5-*b*]indol-4-yl)(methyl)amino)piperidine-1-carboxylate as an off-white solid (96% crude yield), used in the next step without further purification. Purification for analytical purposes was performed by flash column chromatography (SiO_2_, DCM–MeOH gradient elution from 98:2 to 97.5:2.5). ^1^H-NMR (200 MHz, CDCl_3_) δ 8.61 (s, 1H), 8.52 (d, *J* = 1.6 Hz, 1H), 8.09 (d, *J* = 8.2 Hz, 2H), 7.60 (d, *J* = 8.6 Hz, 1H), 7.38 (dd, *J* = 8.5, 1.6 Hz, 1H), 7.26 (d, *J* = 8.1 Hz, 2H, overlap with CHCl_3_ signal), 4.56–3.88 (m, 3H), 3.18–2.94 (m, 4H), 2.79–2.57 (m, 1H), 2.35 (s, 3H), 2.07–1.68 (m, 3H), 1.66–1.51 (m, 1H), 1.40 (s, 9H).; ^13^C NMR (50 MHz, CDCl_3_) δ 160.2, 157.3, 154.8, 154.0, 145.8, 136.2, 135.4, 132.8, 129.8, 128.2, 124.6, 123.4, 120.2, 114.7, 101.2, 80.0, 55.7, 46.7, 44.0 (br), 33.8, 28.5, 28.1, 24.8, 21.8; ESI-MS: (*m*/*z*) 591.8 [M + Na]^+^, 567.7 [M − H]^−^; HPLC method A: t_r_ = 10.548 min.

The crude material obtained from the first step was reacted with K*t*BuO (476.7 mg, 4.25 mmol) in dry THF (19 mL) according to general procedure D (reaction time 1 h). Purification by flash column chromatography (DCM–MeOH gradient elution from 96.5:3.5 to 93:7) gave 183 mg of an off-white solid (73% yield). ^1^H-NMR (200 MHz, CDCl_3_) δ 11.91 (br s, 1H), 8.53 (s, 1H), 7.65 (d, *J* = 8.6 Hz, 1H), 7.46 (s, 1H), 7.21 (d, *J* = 8.3 Hz, 1H, overlap with CHCl_3_ signal), 4.75–3.89 (m, 3H), 3.24 (s, 3H), 3.15–2.95 (m, 1H), 2.82–2.53 (m, 1H), 2.21–1.59 (m, 4H), 1.51 (s, 9H); ^13^C NMR (50 MHz, CDCl_3_) δ 160.1, 156.8, 154.9, 152.1, 137.5, 131.1, 123.6, 121.5, 118.7, 111.7, 98.6, 80.0, 55.1, 46.8 (br), 44.0 (br), 33.4, 28.5, 28.2, 24.9; ESI-MS: (*m*/*z*) 416.0 [M + H]^+^, 437.9 [M + Na]^+^, 413.8 [M − H]^−^; HPLC method A: t_r_ = 9.052 min.

*tert*-Butyl *(R)*-3-((7-bromo-9*H*-pyrimido[4,5-*b*]indol-4-yl)(methyl)amino)piperidine-1-carboxylate (***(R)*-6d**)

The title compound was prepared by a two-step procedure. In the first step, ***(R)*-5d** (400.0 mg, 0.67 mmol), methyl iodide (141.8 mg, 1.0 mmol), and NaH (40.0 mg of a 60% dispersion in mineral oil, 1.0 mmol) were reacted in dry DMF (14 mL) according to general procedure C (reaction time 2.5 h) to afford 382 mg of crude *tert*-butyl *(R)*-3-((7-bromo-9-tosyl-9*H*-pyrimido[4,5-*b*]indol-4-yl)(methyl)amino)piperidine-1-carboxylate as an off-white solid (93% crude yield), used in the next without further purification. Purification of a small portion for analytical purposes was performed by flash column chromatography (SiO_2_, hexane–EtOAc 3:2). ^1^H-NMR (300 MHz, CDCl_3_) δ 8.68 (s, 1H), 8.62 (s, 1H), 8.10 (d, *J* = 8.4 Hz, 2H), 7.60–7.48 (m, 2H), 7.27 (d, *J* = 8.0 Hz, 2H, overlap with CHCl_3_ signal), 4.45–3.97 (m, 3H), 3.11 (s, 3H), 3.09–2.98 (m, 1H), 2.75–2.61 (m, 1H), 2.37 (s, 3H), 1.95–1.72 (m, 3H), 1.59–1.31 (m, 10H); ^13^C NMR (75 MHz, CDCl_3_) δ 160.41, 157.10, 154.69, 154.23, 145.69, 136.14, 135.26, 129.70, 128.02, 127.23, 123.66, 120.65, 120.32, 117.28, 101.06, 79.80, 55.48, 46.56, 43.99 (br), 33.71 (br), 28.36, 27.93, 24.75, 21.68. ESI-MS: (*m*/*z*) 635.9 [M + Na]^+^, 612.1 [M − H]^−^; HPLC method A: t_r_ = 11.342 min.

The crude material obtained from the first step was reacted with K*t*BuO (488.0 mg, 4.35 mmol) in dry THF (20 mL) according to general procedure D (reaction time 45 min). Purification by flash column chromatography (SiO_2_, DCM–MeOH 96:4) gave 175 mg of a white solid (61% yield). ^1^H-NMR (300 MHz, CDCl_3_) δ 11.59 (br s, 1H), 8.58 (s, 1H), 7.72–7.62 (m, 2H), 7.40 (dd, *J* = 8.6, 1.8 Hz, 1H), 4.48–4.02 (m, 3H), 3.26 (s, 3H), 3.14–3.00 (m, 1H), 2.77–2.61 (m, 1H), 2.11–1.75 (m, 3H), 1.70–1.54 (m, 1H), 1.43 (s, 9H); ESI-MS: (*m*/*z*) 482.0 [M + Na]^+^, 458.2 [M − H]^−^; HPLC method A: t_r_ = 9.693 min.

*tert*-Butyl *(S)*-3-((7-chloro-9*H*-pyrimido[4,5-*b*]indol-4-yl)(methyl)amino)piperidine-1-carboxylate (***(S)*-6c**)

The title compound was prepared by a two-step procedure. In the first step, ***(S)*-5c** (465.0 mg, 0.84 mmol), methyl iodide (178.0 mg, 1.25 mmol), and NaH (50.2 mg of a 60% dispersion in mineral oil, 1.25 mmol) were reacted in dry DMF (15 mL) according to general procedure C (reaction time 3.5 h) to afford 440 mg of crude *tert*-Butyl *(S)*-3-((7-chloro-9-tosyl-9*H*-pyrimido[4,5-*b*]indol-4-yl)(methyl)amino)piperidine-1-carboxylate as a yellow solid (92% crude yield), used in the next step without further purification. ESI-MS: (*m*/*z*) 591.8 [M + Na]^+^, 567.7 [M − H]^−^; HPLC method A: t_r_ = 11.053 min.

The crude material obtained from the first step was reacted with K*t*BuO (606.2 mg, 5.40 mmol) in dry THF (24 mL) according to general procedure D (reaction time 1 h). Purification by flash column chromatography (SiO_2_, DCM–MeOH gradient elution from 96.5:3.5 to 92.5:7.5) gave 203 mg of a white solid (63% yield). ^1^H-NMR (300 MHz, CDCl_3_) δ 11.71 (br s, 1H), 8.57 (s, 1H), 7.71 (d, *J* = 8.6 Hz, 1H), 7.53 (d, *J* = 1.7 Hz, 1H), 7.26 (dd, *J* = 8.6, 1.9 Hz, 1H, overlap with CHCl_3_ signal), 4.59–3.97 (m, 3H), 3.27 (s, 3H), 3.16–2.99 (m, 1H), 2.83–2.58 (m, 1H), 2.11–1.54 (m, 4H), 1.43 (s, 9H); ESI-MS: (*m*/*z*) 437.9 [M + Na]^+^, 413.8 [M − H]^−^; HPLC method A: t_r_ = 9.451 min.

*tert*-Butyl *(S)*-3-((7-bromo-9*H*-pyrimido[4,5-*b*]indol-4-yl)(methyl)amino)piperidine-1-carboxylate (***(S)*-6d**)

The title compound was prepared by a two-step procedure. In the first step, ***(S)*-5d** (585.0 mg, 0.97 mmol), NaH (58.4 mg of a 60% dispersion in mineral oil, 1.46 mmol), and methyl iodide (207.4 mg, 1.46 mmol) were reacted in dry DMF (10 mL) according to general procedure C (reaction time 3 h) to afford 578 mg of crude *tert*-butyl *(S)*-3-((7-bromo-9-tosyl-9*H*-pyrimido[4,5-*b*]indol-4-yl)(methyl)amino)piperidine-1-carboxylate as a light yellow solid (96% crude yield), used in the next step without further purification.

The crude material obtained from the first step was reacted with K*t*BuO (639.1 mg, 5.70 mmol) in dry THF (25 mL) according to general procedure D (reaction time 2 h). Purification by flash column chromatography (SiO_2_, DCM–MeOH 96:4) gave 214 mg of a white solid (57% yield). ^1^H-NMR (400 MHz, CDCl_3_) δ 12.09 (s, 1H), 8.56 (s, 1H), 7.66 (d, *J* = 1.6 Hz, 1H), 7.64 (d, *J* = 8.6 Hz, 1H), 7.38 (dd, *J* = 8.6, 1.8 Hz, 1H), 4.61–3.96 (m, 1H), 3.26 (s, 1H), 3.11–3.01 (m, 1H), 2.78–2.61 (m, 1H), 2.09–1.75 (m, 1H), 1.72–1.55 (m, 1H), 1.44 (s, 1H).

##### (5) Detailed Procedures for the Preparation of Intermediates **7a**–**l**

*N*-methyl-*N*-(piperidin-3-yl)-9*H*-pyrimido[4,5-*b*]indol-4-amine (**7a**)

The title compound was prepared from **6a** (60.0 mg, 0.16 mmol) in dry DCM (1 mL) and TFA (0.2 mL) according to general procedure E (reaction time 1.5 h); 36 mg of a light brown solid was yielded (81% crude yield) and used in the next step without further purification. ^1^H-NMR (400 MHz, MeOD) δ 8.36 (s, 1H), 7.84 (d, *J* = 8.0 Hz, 1H), 7.52 (d, *J* = 8.0 Hz, 1H), 7.41 (td, *J* = 7.7, 1.0 Hz, 1H), 7.34–7.27 (m, 1H), 3.30–3.23 (m, 4H), 3.14–3.07 (m, 1H), 3.07–3.00 (m, 1H), 2.72–2.63 (m, 1H), 2.20–1.94 (m, 3H), 1.83–1.70 (m, 1H); ESI-MS: (*m*/*z*) 282.3 [M + H]^+^, 280.3 [M − H]^−^; HPLC method A: t_r_ = 2.232 min.

7-Fluoro-*N*-methyl-*N*-(piperidin-3-yl)-9*H*-pyrimido[4,5-*b*]indol-4-amine (**7b**)

The title compound was prepared from **6b** (170.0 mg, 0.43 mmol) in dry DCM (5 mL) and TFA (1 mL) according to general procedure E (reaction time 1.5 h); 119 mg of a beige solid was yielded (93% crude yield) and used in the next step without further purification. ^1^H-NMR (200 MHz, MeOD) δ 8.33 (s, 1H), 7.78 (dd, *J* = 8.8, 5.2 Hz, 1H), 7.22 (dd, *J* = 9.3, 2.4 Hz, 1H), 7.06 (td, *J* = 9.3, 2.5 Hz, 1H), 4.54–4.37 (m, 1H), 3.24 (s, 3H), 3.17–2.83 (m, 3H), 2.62–2.45 (m, 1H), 2.16–1.55 (m, 4H); ESI-MS: (*m*/*z*) 300.3 [M + H]^+^, 298.3 [M − H]^−^; HPLC method A: t_r_ = 3.036 min.

7-Chloro-*N*-methyl-*N*-(piperidin-3-yl)-9*H*-pyrimido[4,5-*b*]indol-4-amine (**7c**)

The title compound was prepared as described previously [5].

7-Bromo-*N*-methyl-*N*-(piperidin-3-yl)-9*H*-pyrimido[4,5-*b*]indol-4-amine (**7d**)

**6d** (425.0 mg, 0.92 mmol) was stirred in dry DCM (9 mL) and TFA (1.5 mL) at rt for 1 h. The mixture was concentrated under reduced pressure. Residual TFA was neutralized by addition of saturated NaHCO_3_ solution, resulting in a precipitate which was filtered off and washed with saturated NaHCO_3_ solution and demineralized water and was then dried over P_2_O_5_ in vacuo; 285 mg of an off-white solid was yielded (86% crude yield) and used in the next step without further purification. ^1^H-NMR (300 MHz, MeOD) δ 8.35 (s, 1H), 7.70 (d, *J* = 8.6 Hz, 1H), 7.65 (d, *J* = 1.7 Hz, 1H), 7.40 (dd, *J* = 8.6, 1.9 Hz, 1H), 4.55–4.37 (m, 1H), 3.24 (s, 3H), 3.15–3.06 (m, 1H), 3.02–2.95 (m, 1H), 2.95–2.85 (m, 1H), 2.61–2.48 (m, 1H), 2.15–1.84 (m, 3H), 1.78–1.60 (m, 1H); ^13^C NMR (75 MHz, DMSO-*d_6_*) δ 159.6, 157.3, 153.8, 137.7, 124.1, 123.1, 118.9, 117.2, 113.7, 97.1, 55.9, 48.7, 45.5, 32.7, 28.3, 26.5; ESI-MS: (*m*/*z*) 360.1 [M + H]^+^, 358.1 [M − H]^−^; HPLC method A: t_r_ = 4.033 min.

7-Iodo-*N*-methyl-*N*-(piperidin-3-yl)-9*H*-pyrimido[4,5-*b*]indol-4-amine (**7e**)

**6e** (240.0 mg, 0.473 mmol) was suspended in dry DCM (7 mL), and TFA was added (1.5 mL). The mixture was stirred at rt for 30 min and then concentrated under reduced pressure. Residual TFA was neutralized by addition of saturated NaHCO_3_ solution (30 mL), which resulted in a precipitate. The precipitate was filtered off, washed with saturated NaHCO_3_ solution and demineralized water, and dried over P_2_O_5_ in vacuo; 199 mg of a beige solid was yielded (>100% crude yield) and used in the next step without further purification. ^1^H-NMR (300 MHz, MeOD) δ 8.35 (s, 1H), 7.86 (s, 1H), 7.65–7.52 (m, 2H), 4.55–4.40 (m, 1H), 3.24 (s, 3H), 3.14–3.05 (m, 1H), 3.02–2.84 (m, 2H), 2.60–2.47 (m, 1H), 2.15–1.84 (m, 3H), 1.77–1.59 (m, 1H); ESI-MS: (*m*/*z*) 408.3 [M + H]^+^, 406.3 [M − H]^−^; HPLC method A: t_r_ = 4.563 min.

7-methoxy-*N*-methyl-*N*-(piperidin-3-yl)-9*H*-pyrimido[4,5-*b*]indol-4-amine (**7f**)

TFA (0.4 mL) was added to a solution of **6f** (126.0 mg, 0.31 mmol) in dry DCM (10 mL). The mixture was stirred at rt for 1.5 h and then concentrated under reduced pressure. Saturated NaHCO_3_ solution was added to the residue, and the mixture was extracted with EtOAc (3 × 20 mL). Combined organic layers were dried over Na_2_SO_4_ and evaporated to dryness to afford 90 mg (95% crude yield). ^1^H-NMR (300 MHz, DMSO-*d_6_*) δ 11.95 (s, 1H), 8.33 (s, 1H), 7.68 (d, *J* = 8.8 Hz, 1H), 6.97 (d, *J* = 2.4 Hz, 1H), 6.88 (dd, *J* = 8.8, 2.4 Hz, 1H), 4.40–4.26 (m, 1H), 3.84 (s, 3H), 3.13 (s, 3H), 3.06–2.80 (m, 3H), 2.51–2.41 (m, 1H, overlap with DMSO-*d_5_* signal), 2.00–1.71 (m, 3H), 1.60–1.39 (m, 1H); ESI-MS: (*m*/*z*) 312.0 [M + H]^+^, 310.1 [M − H]^−^; HPLC method B: t_r_ = 2.684 min.

*N*-Methyl-*N*-(piperidin-3-yl)-7-(trifluoromethyl)-9*H*-pyrimido[4,5-*b*]indol-4-amine (**7g**)

TFA (1 mL) was added to a solution of **6g** (237.0 mg, 0.53 mmol) in dry DCM (10 mL). The mixture was stirred at rt for 2 h and then concentrated under reduced pressure. Saturated NaHCO_3_ solution was added to the residue, and the mixture was then extracted with EtOAc (3 × 20 mL). Combined organic layers were dried over Na_2_SO_4_ and evaporated to dryness; 194 mg was yielded (100% crude yield) and used in the next step without further purification. ^1^H-NMR (300 MHz, DMSO-*d_6_*) δ 12.30 (br s, 1H), 8.44 (s, 1H), 8.01 (d, *J* = 8.5 Hz, 1H), 7.74 (s, 1H), 7.56 (dd, *J* = 8.5, 1.2 Hz, 1H), 4.54–4.40 (m, 1H), 3.22 (s, 3H), 3.12–3.05 (m, 1H), 3.01–2.86 (m, 2H), 2.58–2.49 (m, 1H, overlap with DMSO-*d_5_* signal), 2.01–1.75 (m, 3H), 1.66–1.49 (m, 1H); HPLC method B: t_r_ = 4.956 min.

6-Chloro-*N*-methyl-*N*-(piperidin-3-yl)-9*H*-pyrimido[4,5-*b*]indol-4-amine (**7h**)

The title compound was prepared from **6h** (215.0 mg, 0.52 mmol) in dry DCM (5.6 mL) and TFA (1.1 mL) according to general procedure E (reaction time 1 h); 163 mg of a yellow solid (100% crude yield) was yielded, used in the next step without further purification. ^1^H-NMR (300 MHz, MeOD) δ 8.35 (s, 1H), 7.76 (d, *J* = 1.8 Hz, 1H), 7.48 (d, *J* = 8.6 Hz, 1H), 7.38 (dd, *J* = 8.6, 2.0 Hz, 1H), 4.54–4.39 (m, 1H), 3.25 (s, 3H), 3.11–2.95 (m, 2H), 2.95–2.85 (m, 1H), 2.63–2.49 (m, 1H), 2.23–1.88 (m, 3H), 1.83–1.65 (m, 1H); ^13^C NMR (101 MHz, DMSO-*d_6_*) δ 159.6, 157.5, 153.9, 135.1, 124.6, 124.3, 121.6, 120.9, 112.5, 96.8, 55.8, 48.8, 45.5, 32.5, 28.4, 26.3; ESI-MS: (*m*/*z*) 316.1 [M + H]^+^, 314.0 [M − H]^−^; HPLC method A: t_r_ = 4.102 min.

6-Bromo-*N*-methyl-*N*-(piperidin-3-yl)-9*H*-pyrimido[4,5-*b*]indol-4-amine (**7i**)

The title compound was prepared from **6i** in dry DCM (6.3 mL) and TFA (1 mL) according to general procedure E (reaction time 1 h). Purification by flash column chromatography (SiO_2_, DCM– (2N NH_3_ in MeOH) 9:1) gave 218 mg of a beige solid (93% yield). ^1^H-NMR (300 MHz, MeOD) δ 8.35 (s, 1H), 7.91 (d, *J* = 1.6 Hz, 1H), 7.52 (dd, *J* = 8.6, 1.8 Hz, 1H), 7.43 (d, *J* = 8.6 Hz, 1H), 4.51–4.40 (m, 1H), 3.24 (s, 3H), 3.08–2.95 (m, 2H), 2.94–2.85 (m, 1H), 2.61–2.48 (m, 1H), 2.22–1.89 (m, 3H), 1.84–1.66 (m, 1H); ^13^C NMR (75 MHz, DMSO-*d_6_*) δ 159.6, 157.4, 154.0, 135.4, 127.1, 124.6, 121.6, 113.1, 112.5, 96.7, 56.0, 48.9, 45.5, 32.5, 28.6, 26.5; ESI-MS: (*m*/*z*) 360.1 [M + H]^+^, 358.1 [M − H]^−^; HPLC method A: t_r_ = 4.058 min.

6-Methoxy-*N*-methyl-*N*-(piperidin-3-yl)-9*H*-pyrimido[4,5-*b*]indol-4-amine (**7j**)

The title compound was prepared from **6j** (325.0 mg, 0.78 mmol) in dry DCM (10 mL) and TFA (2 mL) according to general procedure E (reaction time 2 h); 207 mg of a beige solid (84% yield) was yielded. ^1^H-NMR (200 MHz, MeOD) δ 8.32 (s, 1H), 7.43 (d, *J* = 8.8 Hz, 1H), 7.29 (d, *J* = 2.4 Hz, 1H), 7.07 (dd, *J* = 8.9, 2.3 Hz, 1H), 4.48–4.29 (m, 1H), 3.89 (s, 3H), 3.22 (s, 3H), 3.13–2.81 (m, 3H), 2.60–2.44 (m, 1H), 2.23–1.84 (m, 3H), 1.82–1.58 (m, 1H); ESI-MS: (*m*/*z*) 312.2 [M + H]^+^, 310.2 [M − H]^−^; HPLC method A: t_r_ = 2.084 min.

5-Chloro-*N*-methyl-*N*-(piperidin-3-yl)-9*H*-pyrimido[4,5-*b*]indol-4-amine (**7k**)

The title compound was prepared from **6k** (340.0 mg, 0.82 mmol) in dry DCM (9 mL) and TFA (1.8 mL) according to general procedure E (reaction time 2 h); 244 mg of a beige solid (95% crude yield) was yielded, used in the next step without further purification. ^1^H-NMR (300 MHz, MeOD) δ 8.31 (s, 1H), 7.43 (dd, *J* = 7.9, 1.2 Hz, 1H), 7.35 (t, *J* = 7.8 Hz, 1H), 7.26 (dd, *J* = 7.7, 1.2 Hz, 1H), 4.52–4.33 (m, 1H), 3.19–2.90 (m, 5H), 2.89–2.68 (m, 1H), 2.57–2.41 (m, 1H), 2.20–1.97 (m, 1H), 1.96–1.74 (m, 2H), 1.73–1.56 (m, 1H); ESI-MS: (*m*/*z*) 316.1 [M + H]^+^, 314.1 [M − H]^−^; HPLC method A: t_r_ = 3.814 min.

7-Chloro-*N*,2-dimethyl-*N*-(piperidin-3-yl)-9*H*-pyrimido[4,5-*b*]indol-4-amine (**7l**)

The title compound was prepared from **6l** (310.0 mg, 0.72 mmol) in TFA (2 mL) and dry DCM (10 mL) according to general procedure E (reaction time 2.5 h); 239 mg of a yellow solid (100% crude yield) was yielded, used in the next step without further purification. ^1^H-NMR (300 MHz, MeOD) δ 7.70 (d, *J* = 8.6 Hz, 1H), 7.46 (d, *J* = 1.9 Hz, 1H), 7.22 (dd, *J* = 8.6, 2.0 Hz, 1H), 4.57–4.44 (m, 1H), 3.23 (s, 3H), 3.15–3.06 (m, 1H), 3.02–2.93 (m, 1H), 2.93–2.84 (m, 1H), 2.59–2.47 (m, 4H), 2.13–1.84 (m, 3H), 1.77–1.59 (m, 1H); ^13^C NMR (101 MHz, DMSO-*d_6_*) δ 162.5, 159.6, 158.4, 137.3, 128.6, 123.3, 120.2, 118.8, 110.7, 94.9, 55.8, 48.8, 45.5, 32.4, 28.3, 26.4, 25.9; ESI-MS: (*m*/*z*) 330.1 [M + H]^+^, 328.1 [M − H]^−^; HPLC method A: t_r_ = 4.602 min.

##### (6) Detailed Procedures for the Preparation of Enantiopure Intermediates ***(R)*-7c,d** and ***(S)*-7c,d**

*(R)*-7-Chloro-*N*-methyl-*N*-(piperidin-3-yl)-9*H*-pyrimido[4,5-*b*]indol-4-amine (***(R)*-7c**)

The title compound was prepared from ***(R)*-6c** (160.0 mg, 0.39 mmol) in dry DCM (5 mL) and TFA (1 mL) according to general procedure E (reaction time 45 min); 120 mg of a yellow solid (99% crude yield) was yielded, used in the next step without further purification. ^1^H-NMR (400 MHz, MeOD) δ 8.35 (s, 1H), 7.78 (d, *J* = 8.6 Hz, 1H), 7.50 (d, *J* = 1.9 Hz, 1H), 7.28 (dd, *J* = 8.6, 2.0 Hz, 1H), 4.56–4.47 (m, 1H), 3.26 (s, 3H), 3.20–3.13 (m, 1H), 3.06–3.00 (m, 1H), 2.99–2.92 (m, 1H), 2.64–2.55 (m, 1H), 2.16–1.90 (m, 3H), 1.78–1.65 (m, 1H); ESI-MS: (*m*/*z*) 316.0 [M + H]^+^, 338.0 [M + Na]^+^, 313.9 [M − H]^−^; HPLC method A: t_r_ = 3.411 min.

*(R)*-7-Bromo-*N*-methyl-*N*-(piperidin-3-yl)-9*H*-pyrimido[4,5-*b*]indol-4-amine (***(R)*-7d**)

The title compound was prepared from ***(R)*-6d** (175.0 mg, 0.38 mmol) in dry DCM (5 mL) and TFA (1 mL) according to general procedure E (reaction time 2.5 h); 135 mg of a beige solid (99% crude yield) was yielded, used in the next step without further purification. ^1^H-NMR (300 MHz, MeOD) δ 8.35 (s, 1H), 7.70 (d, *J* = 8.6 Hz, 1H), 7.65 (d, *J* = 1.8 Hz, 1H), 7.40 (dd, *J* = 8.6, 1.9 Hz, 1H), 4.54–4.41 (m, 1H), 3.24 (s, 3H), 3.15–3.06 (m, 1H), 3.02–2.95 (m, 1H), 2.94–2.84 (m, 1H), 2.61–2.48 (m, 1H), 2.15–1.85 (m, 3H), 1.77–1.60 (m, 1H); ESI-MS: (*m*/*z*) 360.0 [M + H]^+^, 358.1 [M − H]^−^; HPLC method A: t_r_ = 3.985 min.

*(S)*-7-Chloro-*N*-methyl-*N*-(piperidin-3-yl)-9*H*-pyrimido[4,5-*b*]indol-4-amine (***(S)*-7c**)

The title compound was prepared from ***(S)*-6c** (180.0 mg, 0.43 mmol) in dry DCM (5 mL) and TFA (1 mL) according to general procedure E (reaction time 1.5 h); 142 mg of a beige solid (>100% crude yield) was yielded, used in the next step without further purification. ^1^H-NMR (300 MHz, MeOD) δ 8.34 (s, 1H), 7.76 (d, *J* = 8.6 Hz, 1H), 7.49 (d, *J* = 1.9 Hz, 1H), 7.27 (dd, *J* = 8.6, 2.0 Hz, 1H), 4.58–4.41 (m, 1H), 3.25 (s, 3H), 3.19–3.09 (m, 1H), 3.05–2.97 (m, 1H), 2.97–2.86 (m, 1H), 2.64–2.50 (m, 1H), 2.19–1.83 (m, 3H), 1.80–1.59 (m, 1H); ESI-MS: (*m*/*z*) 316.0 [M + H]^+^, 338.0 [M + Na]^+^, 313.9 [M − H]^−^; HPLC method A: t_r_ = 3.897 min.

*(S)*-7-Bromo-*N*-methyl-*N*-(piperidin-3-yl)-9*H*-pyrimido[4,5-*b*]indol-4-amine (***(S)*-7d**)

The title compound was prepared from ***(S)*-6d** (190.0 mg, 0.41 mmol) in dry DCM (5 mL) and TFA (1 mL) according to general procedure E (reaction time 1 h); 141 mg of a beige solid (95% crude yield) was yielded, used in the next step without further purification. ^1^H-NMR (400 MHz, MeOD) δ 8.35 (s, 1H), 7.69 (d, *J* = 8.6 Hz, 1H), 7.65 (d, *J* = 1.8 Hz, 1H), 7.40 (dd, *J* = 8.6, 1.9 Hz, 1H), 4.53–4.43 (m, 1H), 3.24 (s, 3H), 3.14–3.08 (m, 1H), 3.02–2.95 (m, 1H), 2.95–2.87 (m, 1H), 2.59–2.50 (m, 1H), 2.12–1.86 (m, 3H), 1.75–1.62 (m, 1H); ESI-MS: (*m*/*z*) 360.2 [M + H]^+^, 358.2 [M − H]^−^; HPLC method A: t_r_ = 4.025 min.

##### (7) Detailed Procedures for the Preparation of Final Compounds **8**–**41**

(3-((7-Chloro-9*H*-pyrimido[4,5-*b*]indol-4-yl)(methyl)amino)piperidin-1-yl)(furan-3-yl)methanone (**8**)

The title compound was prepared from **7c** (64.0 mg, 0.20 mmol), 3-furoic acid (27.3 mg, 0.24 mmol), PyBOP (130.8 mg, 0.24 mmol), and DIPEA (78.6 mg, 0.61 mmol) in dry DCM (total amount 10 mL) according to general procedure F (reaction time 1.5 h). Purification by flash column chromatography (SiO_2_, DCM–MeOH gradient elution from 96.5:3.5 to 93.5:6.5) gave 50 mg of a white solid (60% yield). ^1^H-NMR (400 MHz, DMSO-*d_6_*) δ 12.25 (s, 1H), 8.38 (s, 1H), 8.26–7.97 (m, 1H), 7.92–7.77 (m, 1H), 7.73 (t, *J* = 1.7 Hz, 1H), 7.48 (d, *J* = 2.0 Hz, 1H), 7.24 (dd, *J* = 8.6, 2.1 Hz, 1H), 6.85–6.58 (m, 1H), 4.81–3.79 (m, 3H), 3.30–2.56 (m, 5H), 2.15–1.94 (m, 2H), 1.94–1.74 (m, 1H), 1.63–1.47 (m, 1H); ESI-MS: (m/z) 410.0 [M + H]^+^, 432.0 [M + Na]^+^, 407.9 [M − H]^−^; HPLC method A: t_r_ = 7.624 min.

Ethyl 3-(3-((7-chloro-9*H*-pyrimido[4,5-*b*]indol-4-yl)(methyl)amino)piperidin-1-yl)-3-oxopropanoate (**9**)

The title compound was prepared from **7c** (70.0 mg, 0.22 mmol), ethyl potassium malonate (45.3 mg, 0.27 mmol), PyBOP (138.4 mg, 0.27 mmol), and DIPEA (86.0 mg, 0.67 mmol) in dry DCM (total amount 10 mL) according to general procedure F. Additional ethyl potassium malonate (5.7 mg, 0.03 mmol) and PyBOP (17.3 mg, 0.03 mmol) were added after a reaction time of 2.5 h, and then stirring continued for 1 h. Purification twice by flash column chromatography (SiO_2_, DCM–MeOH gradient elution from 97.5:2.5 to 93.5:6.5 and SiO_2_, DCM–EtOH gradient elution from 95:5 to 93:7) gave 50 mg of a white solid (53% yield). ^1^H-NMR shows a 3:2 mixture of amide bond rotamers. ^1^H-NMR (400 MHz, DMSO-*d_6_*) δ 12.32–12.14 (m, 1H), 8.46–8.34 (m, 1H), 7.86–7.77 (m, 1H), 7.52–7.44 (m, 1H), 7.32–7.16 (m, 1H), 4.61–4.50 (m, 0.6H), 4.42–4.34 (m, 0.4H), 4.33–4.16 (m, 1H), 4.10–3.95 (m, 2H), 3.95–3.90 (m, 0.4H), 3.79–3.68 (m, 0.6H), 3.64–3.47 (m, 2H), 3.33–3.28 (m, 0.4H, overlap with water signal), 3.28–3.15 (m, 3H), 3.08–2.95 (m, 1.2H), 2.63–2.54 (m, 0.4H), 2.10–1.74 (m, 3H), 1.58–1.40 (m, 1H), 1.17–1.08 (m, 3H); ^13^C NMR (101 MHz, DMSO-*d_6_*) δ 167.7, 167.6, 164.5, 164.4, 159.43, 159.40, 157.51, 157.48, 153.7, 153.6, 137.44, 137.40, 129.3, 124.03, 123.97, 120.5, 120.4, 118.4, 118.3, 110.83, 110.81, 97.4, 97.2, 60.5, 60.4, 54.7, 54.5, 47.6, 45.7, 44.0, 41.5, 40.9, 40.8, 33.9, 32.6, 27.2, 27.1, 24.8, 24.3, 13.89, 13.86; ESI-MS: (*m*/*z*) 430.0 [M + H]^+^, 451.9 [M + Na]^+^, 427.8 [M − H]^−^; HPLC method A: t_r_ = 7.274 min.

(3-((7-Chloro-9*H*-pyrimido[4,5-*b*]indol-4-yl)(methyl)amino)piperidin-1-yl)(4-(dimethylamino)phenyl)methanone (**10**)

The title compound was prepared from **7c** (60.0 mg, 0.19 mmol), 4-(dimethylamino) benzoic acid (37.7 mg, 0.22 mmol), PyBOP (118.6 mg, 0.22 mmol), and DIPEA (73.7 mg, 0.57 mmol) in dry DCM (total amount 7 mL) according to general procedure F (reaction time 40 min). Purification by flash column chromatography (SiO_2_, DCM–MeOH gradient elution from 95.5:4.5 to 93.5:6.5) gave 70 mg of an off-white solid (80% yield). ^1^H-NMR (400 MHz, DMSO-*d_6_*) δ 12.25 (s, 1H), 8.39 (s, 1H), 7.80 (d, *J* = 8.0 Hz, 1H), 7.50 (d, *J* = 1.9 Hz, 1H), 7.25 (dd, *J* = 8.6, 2.0 Hz, 1H), 7.17 (d, *J* = 7.8 Hz, 2H), 6.60 (d, *J* = 7.4 Hz, 2H), 4.50–3.85 (m, 3H), 3.28–3.13 (m, 4H), 3.04–2.78 (m, 7H), 2.12–1.99 (m, 2H), 1.93–1.76 (m, 1H), 1.65–1.46 (m, 1H); ESI-MS: (*m*/*z*) 463.9 [M + H]^+^, 485.9 [M + Na]^+^, 461.8 [M − H]^−^; HPLC method A: t_r_ = 8.928 min.

1-(3-((7-Chloro-9*H*-pyrimido[4,5-*b*]indol-4-yl)(methyl)amino)piperidin-1-yl)butan-1-one (**11**)

The title compound was prepared from **7c** (60.0 mg, 0.19 mmol), butyric acid (20.9 mg, 0.24 mmol), TBTU (76.3 mg, 0.24 mmol), and DIPEA (73.7 mg, 0.57 mmol) in dry DCM (total amount 10 mL) according to general procedure F (reaction time 1 h). Purification by flash column chromatography (SiO_2_, DCM–MeOH 94.5:5.5) gave 54 mg of a white solid (74% yield). ^1^H-NMR shows a 5:4 mixture of amide bond rotamers. ^1^H-NMR (400 MHz, CDCl_3_) δ 12.42 (br s, 1H), 8.58–8.42 (m, 1H), 7.74–7.59 (m, 1H), 7.51–7.38 (m, 1H), 7.25–7.16 (m, 1H), 5.05–4.86 (m, 0.45H), 4.81–4.65 (m, 0.55H), 4.53–4.29 (m, 1H), 4.28–4.16 (m, 0.55H), 3.97–3.81 (m, 0.45H), 3.37–3.19 (m, 3H), 3.19–3.10 (m, 0.55H), 3.05–2.89 (m, 0.9H), 2.57–2.30 (m, 2.55H), 2.22–1.85 (m, 3H), 1.82–1.53 (m, 3H), 0.98 (t, *J* = 7.3 Hz, 3H); ^13^C NMR (101 MHz, CDCl_3_) δ 172.4, 171.9, 160.1, 157.7, 157.3, 152.8, 152.6, 137.6, 137.5, 131.2, 131.1, 123.8, 123.7, 121.5, 121.4, 118.7, 111.7, 111.5, 98.8, 98.5, 55.3, 55.2, 47.8, 46.0, 45.0, 42.2, 35.7, 34.4, 33.4, 28.8, 28.0, 25.6, 25.0, 19.0, 14.3, 14.2; ESI-MS: (*m*/*z*) 408.3 [M + Na]^+^, 384.3 [M − H]^−^; HPLC method A: t_r_ = 8.285 min.

1-(3-((7-Chloro-9*H*-pyrimido[4,5-*b*]indol-4-yl)(methyl)amino)piperidin-1-yl)-3-methylbutan-1-one (**12**)

The title compound was prepared from **7c** (60.0 mg, 0.19 mmol), isovaleric acid (22.3 mg, 0.22 mmol), TBTU (76.3 mg, 0.24 mmol), and DIPEA (73.7 mg, 0.57 mmol) in dry DCM (total amount 10 mL) according to general procedure F (reaction time 1 h). Purification by flash column chromatography (SiO_2_, DCM–MeOH 95:5) gave 61 mg of a white solid (80% yield). ^1^H-NMR shows a 5:4 mixture of amide bond rotamers. ^1^H-NMR (400 MHz, CDCl_3_) δ 12.30 (br s, 1H), 8.56–8.47 (m, 1H), 7.73–7.63 (m, 1H), 7.50–7.41 (m, 1H), 7.25–7.19 (m, 1H), 5.04–4.93 (m, 0.45H), 4.80–4.70 (m, 0.55H), 4.52–4.29 (m, 1H), 4.28–4.20 (m, 0.55H), 3.97–3.85 (m, 0.45H), 3.34–3.20 (m, 3H), 3.18–3.10 (m, 0.55H), 3.05–2.91 (m, 0.9H), 2.55–2.46 (m, 0.55H), 2.45–2.24 (m, 2H), 2.23–1.86 (m, 4H), 1.75–1.54 (m, 1H), 1.01–0.93 (m, 6H); ^13^C NMR (101 MHz, CDCl_3_) δ 171.9, 171.4, 160.1, 157.7, 157.3, 152.8, 152.6, 137.6, 137.5, 131.22, 131.15, 123.8, 123.7, 121.6, 121.4, 118.7, 111.7, 111.5, 98.9, 98.6, 55.4, 55.3, 48.0, 46.2, 45.1, 42.5, 42.3, 34.4, 33.4, 28.8, 28.0, 26.0, 25.9, 25.7, 25.0, 22.93, 22.89; ESI-MS: (*m*/*z*) 422.3 [M + Na]^+^, 398.3 [M − H]^−^; HPLC method A: t_r_ = 8.646 min.

1-(3-((7-Chloro-9*H*-pyrimido[4,5-*b*]indol-4-yl)(methyl)amino)piperidin-1-yl)-3-(dimethylamino)propan-1-one (**13**)

The title compound was prepared from **7c** (75.0 mg, 0.24 mmol), 3-(dimethylamino) propionic acid hydrochloride (47.4 mg, 0.31 mmol), PyBOP (160.7 mg, 0.31 mmol), and TEA (72.1 mg, 0.71 mmol) in dry DCM (total amount 10 mL) according to general procedure F (reaction time 1.5 h). During the extractive work-up the organic layer was not washed with saturated NH_4_Cl solution due to the basic amino function of the introduced substituent. Purification twice by flash column chromatography (SiO_2_, DCM–(2N NH_3_ in MeOH) gradient elution from 95:5 to 9:1 and SiO_2_, DCM:(2N NH_3_ in MeOH) gradient elution from 92.5:7.5 to 9:1) gave 60 mg of a white solid (61% yield). ^1^H-NMR shows a 3:2 mixture of amide bond rotamers. ^1^H-NMR (400 MHz, CDCl_3_) δ 13.05–12.30 (m, 1H), 8.52–8.41 (m, 1H), 7.65–7.56 (m, 1H), 7.44–7.36 (m, 1H), 7.21–7.11 (m, 1H), 4.96–4.83 (m, 0.4H), 4.76–4.62 (m, 0.6H), 4.47–4.20 (m, 1.6H), 3.95–3.84 (m, 0.4H), 3.32–3.17 (m, 3H), 3.17–3.08 (m, 0.6H), 3.03–2.95 (m, 0.4H), 2.93–2.86 (m, 0.4H), 2.85–2.57 (m, 4H), 2.55–2.45 (m, 0.6H), 2.32 (s, 6H), 2.18–1.83 (m, 3H), 1.73–1.52 (m, 1H); ^13^C NMR (101 MHz, CDCl_3_) δ 170.7, 170.2, 160.1, 160.0, 157.8, 157.7, 153.0, 137.7, 137.6, 131.0, 130.9, 123.7, 123.6, 121.22, 121.15, 118.7, 118.6, 111.6, 111.4, 98.8, 98.5, 55.3, 55.2, 54.9, 47.6, 46.0, 45.4, 45.3, 44.9, 42.3, 34.4, 33.4, 31.8, 31.5, 28.7, 27.9, 25.5, 24.8; ESI-MS: (*m*/*z*) 415.4 [M + H]^+^, 413.2 [M − H]^−^; HPLC *method A*: t_r_ = 4.833 min.

1-(3-((7-Chloro-9*H*-pyrimido[4,5-*b*]indol-4-yl)(methyl)amino)piperidin-1-yl)ethan-1-one (**14**)

The title compound was prepared from **7c** (70.0 mg, 0.22 mmol), acetic acid (20.0 mg, 0.33 mmol), PyBOP (144.4 mg, 0.28 mmol), and DIPEA (86.1 mg, 0.67 mmol) in dry DCM (total amount 12 mL) according to general procedure F (reaction time 1.5 h). Purification twice by flash column chromatography (SiO_2_, DCM–MeOH gradient elution from 96:4 to 93.5:6.5 and SiO_2_, EtOAc/MeOH 9:1) gave 44 mg of an off-white solid (55% yield). ^1^H-NMR shows a 5:4 mixture of amide bond rotamers. ^1^H-NMR (300 MHz, DMSO-*d_6_*) δ 12.30–12.14 (m, 1H), 8.41 (s, 1H), 7.87–7.78 (m, 1H), 7.51–7.44 (m, 1H), 7.30–7.17 (m, 1H), 4.57–4.47 (m, 0.55H), 4.43–4.12 (m, 1.45H), 4.07–3.95 (m, 0.45H), 3.84–3.72 (m, 0.55H), 3.33–3.16 (m, 3.45H), 3.07–2.85 (m, 1.1H), 2.57–2.44 (m, 0.45H, overlap with DMSO-*d_5_* signal), 2.10–1.86 (m, 5H), 1.86–1.73 (m, 1H), 1.58–1.35 (m, 1H); ^13^C NMR (101 MHz, DMSO-*d_6_*) δ 168.3, 168.2, 159.5, 159.4, 157.50, 157.47, 153.7, 153.6, 137.4, 129.2, 124.0, 123.9, 120.4, 120.3, 118.4, 118.3, 110.8, 97.4, 97.1, 54.7, 54.5, 47.6, 45.7, 43.6, 41.0, 33.8, 32.6, 27.3, 27.1, 25.1, 24.4, 21.3; ESI-MS: (*m*/*z*) 380.4 [M + Na]^+^, 356.5 [M − H]^−^; HPLC method A: t_r_ = 6.993 min.

1-(3-((7-Chloro-9*H*-pyrimido[4,5-*b*]indol-4-yl)(methyl)amino)piperidin-1-yl)-2-cyclopropylethan-1-one (**15**)

The title compound was prepared from **7c** (50.0 mg, 0.16 mmol), 2-cyclopropylacetic acid (19.8 mg, 0.20 mmol), TBTU (63.6 mg, 0.20 mmol), and DIPEA (61.4 mg, 0.48 mmol) in dry DCM (total amount 10 mL) according to general procedure F (reaction time 1.5 h). Purification by flash column chromatography (SiO_2_, DCM–MeOH gradient elution from 96:4 to 93.5:6.5) gave 52 mg of a beige solid (83% yield). ^1^H-NMR shows a 5:4 mixture of amide bond rotamers. ^1^H-NMR (300 MHz, CDCl_3_) δ 12.20 (br s, 1H), 8.72–8.27 (m, 1H), 7.79–7.59 (m, 1H), 7.56–7.39 (m, 1H), 7.29–7.17 (m, 1H, overlap with CHCl_3_ signal), 5.07–4.88 (m, 0.45H), 4.81–4.66 (m, 0.55H), 4.58–4.30 (m, 1H), 4.27–4.12 (m, 0.55H), 3.94–3.79 (m, 0.45H), 3.38–2.91 (m, 4.55H), 2.60–2.29 (m, 2.45H), 2.25–1.83 (m, 3H), 1.81–1.52 (m, 1H), 1.18–0.96 (m, 1H), 0.65–0.40 (m, 2H), 0.34–0.08 (m, 2H); ^13^C NMR (101 MHz, CDCl_3_) δ 172.0, 171.5, 160.0, 157.6, 157.0, 152.7, 152.4, 137.6, 131.2, 123.9, 123.6, 121.6, 121.4, 118.7, 111.7, 98.8, 55.3, 55.2, 47.9, 46.2, 45.1, 42.2, 38.9, 38.8, 34.4, 33.5, 28.8, 28.0, 25.6, 24.9, 7.5, 4.70, 4.66; ESI-MS: (*m*/*z*) 398.3 [M + H]^+^, 420.3 [M + Na]^+^, 396.3 [M − H]^−^; HPLC method A: t_r_ = 8.031 min.

1-(3-((7-Chloro-9*H*-pyrimido[4,5-*b*]indol-4-yl)(methyl)amino)piperidin-1-yl)propan-1-one (**16**)

The title compound was prepared from **7c** (60.0 mg, 0.17 mmol), propionic acid (16.9 mg, 0.23 mmol), PyBOP (118.6 mg, 0.23 mmol). and DIPEA (73.7 mg, 0.57 mmol) in dry DCM (total amount 10 mL) according to general procedure F (reaction time 40 min). Purification by flash column chromatography (SiO_2_, DCM–MeOH gradient elution from 96:4 to 93.5:6.5) gave 38 mg of a white solid (54% yield). ^1^H-NMR shows a 5:4 mixture of amide bond rotamers. ^1^H-NMR (400 MHz, CDCl_3_) δ 12.58 (br s, 1H), 8.59–8.37 (m, 1H), 7.74–7.58 (m, 1H), 7.52–7.40 (m, 1H), 7.24–7.15 (m, 1H), 5.02–4.88 (m, 0.45H), 4.81–4.67 (m, 0.55H), 4.52–4.30 (m, 1H), 4.29–4.18 (m, 0.55H), 3.95–3.81 (m, 0.45H), 3.37–3.19 (m, 3H), 3.19–3.07 (m, 0.55H), 3.07–2.85 (m, 1H), 2.65–2.34 (m, 2.45H), 2.21–1.83 (m, 3H), 1.76–1.54 (m, 1H), 1.26–1.12 (m, 3H); ^13^C NMR (101 MHz, CDCl_3_) δ 173.2, 172.7, 159.92, 159.88, 157.2, 156.5, 152.4, 151.8, 137.6, 137.5, 131.1, 123.8, 123.6, 121.5, 121.3, 118.6, 111.72, 111.68, 98.5, 98.4, 55.2, 55.1, 47.5, 45.8, 44.9, 42.2, 34.5, 33.5, 28.7, 28.0, 26.9, 26.8, 25.5, 24.9, 9.8; ESI-MS: (*m*/*z*) 393.9 [M + Na]^+^, 369.8 [M − H]^−^; HPLC method A: t_r_ = 7.599 min.

1-(3-((7-Chloro-9*H*-pyrimido[4,5-*b*]indol-4-yl)(methyl)amino)piperidin-1-yl)-3,3,3-trifluoropropan-1-one (**17**)

The title compound was prepared from **7c** (65.0 mg, 0.21 mmol), trifluoropropionic acid (33.0 mg, 0.26 mmol), TBTU (82.6 mg, 0.26 mmol), and DIPEA (79.8 mg, 0.62 mmol) in dry DCM (total amount 10 mL) according to general procedure F (reaction time 2 h). Purification by flash column chromatography (SiO_2_, DCM–MeOH 95:5) gave 44 mg of a light yellow solid (50% yield). ^1^H-NMR shows a 3:2 mixture of amide bond rotamers. ^1^H-NMR (300 MHz, DMSO-*d_6_*) δ 12.23 (s, 1H), 8.44–8.33 (m, 1H), 7.89–7.76 (m, 1H), 7.54–7.44 (m, 1H), 7.30–7.16 (m, 1H), 4.65–4.52 (m, 0.6H), 4.43–4.16 (m, 1.4H), 4.05–3.95 (m, 0.4H), 3.84–3.62 (m, 2.6H), 3.34–3.17 (m, 3.4H), 3.08–2.93 (m, 1.2H), 2.68–2.54 (m, 0.4H), 2.11–1.72 (m, 3H), 1.62–1.40 (m, 1H); ESI-MS: (*m*/*z*) 447.8 [M + Na]^+^, 423.8 [M − H]^−^; HPLC method A: t_r_ = 8.124 min.

1-(3-((7-chloro-9*H*-pyrimido[4,5-*b*]indol-4-yl)(methyl)amino)piperidin-1-yl)-2,2-dimethylpropan-1-one (**38**)

**7c** (60.0 mg, 0.19 mmol), pivalic acid (24.3 mg, 0.24 mmol), TBTU (70.3 mg, 0.24 mmol), and DIPEA (73.7 mg, 0.57 mmol) were stirred in dry DCM (10 mL) at rt and under N_2_ atmosphere, but no conversion was observed. Therefore, the stirring mixture was cooled down to 0 °C, and pivaloyl chloride (237 µL of a freshly prepared 1-M solution in dry DCM, 0.24 mmol) was added. The cooling was removed, and the mixture was stirred for 45 min under N_2_ atmosphere. Saturated NH_4_Cl solution (20 mL) was added, and the mixture was diluted with DCM. Phases were separated, and the organic layer was washed with saturated NH_4_Cl solution (20 mL) and saturated NaHCO_3_ solution (2 × 20 mL), then dried over Na_2_SO_4_, and concentrated under reduced pressure. Purification of the residue by flash column chromatography (DCM–MeOH 95:5) gave 36 mg of a white solid (47% yield). ^1^H-NMR (300 MHz, CDCl_3_) δ 12.39 (br s, 1H), 8.49 (s, 1H), 7.67 (d, *J* = 8.5 Hz, 1H), 7.46 (s, 1H), 7.21 (d, *J* = 8.3 Hz, 1H), 4.85–4.68 (m, 1H), 4.59–4.37 (m, 2H), 3.29 (s, 3H), 3.12–3.00 (m, 1H), 2.82–2.67 (m, 1H), 2.17–1.85 (m, 3H), 1.75–1.57 (m, 1H), 1.31 (s, 9H); ^13^C NMR (101 MHz, CDCl_3_) δ 177.0, 160.0, 157.4, 152.5, 137.6, 131.1, 123.7, 121.3, 118.7, 111.6, 98.6, 55.2, 47.8, 45.7, 39.0, 33.9, 28.6, 28.5, 25.2; ESI-MS: (*m*/*z*) 400.5 [M + H]^+^, 422.5 [M + Na]^+^, 398.5 [M − H]^−^; HPLC method A: t_r_ = 8.748 min.

(3-((7-chloro-9*H*-pyrimido[4,5-*b*]indol-4-yl)(methyl)amino)piperidin-1-yl)(cyclopropyl)methanone (**39**)

Cyclopropanecarbonyl chloride (190 µL of a freshly prepared 1M solution in dry THF, 0.19 mmol) was slowly added to an ice-cooled stirring solution of **7c** (50.0 mg, 0.16 mmol) and TEA (32.0 mg, 0.32 mmol) in dry THF (10 mL) under N_2_ atmosphere. The mixture was left to warm to rt and stirred under N_2_ atmosphere. Additional cyclopropanecarbonyl chloride solution was added after 1 h (79 µL, 0.08 mmol) and 2 h (158 µL, 0.16 mmol) after cooling down the mixture each time; however, full consumption of the starting material was not achieved. The mixture was evaporated to dryness. Purification of the residue by flash column chromatography (DCM–MeOH 95:5) gave 46 mg of a white solid (76% yield). ^1^H-NMR shows a 5:4 mixture of amide bond rotamers. ^1^H-NMR (400 MHz, DMSO-*d_6_*) δ 12.23 (s, 1H), 8.41 (s, 1H), 7.86–7.76 (m, 1H), 7.48 (s, 1H), 7.32–7.14 (m, 1H), 4.63–4.48 (m, 0.55H), 4.44–4.14 (m, 2.45H), 3.30–3.14 (m, 3H), 3.12–2.92 (m, 1H), 2.64–2.54 (m, 0.45H), 2.12–1.74 (m, 4H), 1.56–1.38 (m, 1H), 0.78–0.55 (m, 4H), missing 0.45H below water signal; ^13^C NMR (101 MHz, CDCl_3_) δ 172.5, 160.1, 157.2 (br), 152.5 (br), 137.6, 131.1, 123.7, 121.4, 118.7, 111.6, 98.7, 55.3, 47.9 (br), 45.9 (br), 42.9 (br), 34.2 (br), 28.7 (br), 24.9 (br), 11.4, 7.6, 7.4; ESI-MS: (*m*/*z*) 384.2 [M + H]^+^, 406.2 [M + Na]^+^, 382.2 [M − H]^−^; HPLC method A: t_r_ = 7.837 min.

1-(3-((7-Chloro-9*H*-pyrimido[4,5-*b*]indol-4-yl)(methyl)amino)piperidin-1-yl)prop-2-en-1-one (**40**)

Acryloyl chloride (228 µL of a freshly prepared 1M solution in dry THF, 0.23 mmol) was slowly added to a stirring solution of **7c** (60.0 mg, 0.19 mmol) and TEA (38.5 mg, 0.38 mmol) in dry THF (10 mL) under N_2_ atmosphere and ice/MeOH cooling. The mixture was left to warm to rt and stirred until reaction control indicated sufficient conversion. Extractive work-up followed by flash column chromatography (DCM–MeOH 95:5) gave 50 mg of a white solid (71% yield). ^1^H-NMR shows a 5:4 mixture of amide bond rotamers. ^1^H-NMR (400 MHz, DMSO-*d_6_*) δ 12.23 (s, 1H), 8.41 (s, 1H), 7.88–7.75 (m, 1H), 7.48 (d, *J* = 1.8 Hz, 1H), 7.22 (d, *J* = 8.3 Hz, 1H), 6.94–6.74 (m, 1H), 6.15–6.03 (m, 1H), 5.74–5.62 (m, 1H), 4.65–4.54 (m, 0.55H), 4.50–4.38 (m, 0.45H), 4.34–4.19 (m, 1.45H), 4.10–3.98 (m, 0.55H), 3.29–3.16 (m, 3H), 3.10–2.97 (m, 1.1H), 2.70–2.58 (m, 0.45H), 2.10–1.78 (m, 3H), 1.53–1.37 (m, 1H), missing 0.45H below water signal; ^13^C NMR (101 MHz, DMSO-*d_6_*) δ 164.49, 159.40, 157.45, 153.58, 137.40, 129.23, 128.45, 126.98, 123.91, 120.33, 118.34, 110.81, 97.33, 55.07, 54.43, 47.22, 45.09, 44.16, 41.65, 33.63, 32.67, 27.22, 25.37, 24.31; ESI-MS: (*m*/*z*) 370.1 [M + H]^+^, 392.2 [M + Na]^+^, 368.1 [M − H]^−^; HPLC method A: t_r_ = 7.384 min.

2-(3-(3-((7-Chloro-9*H*-pyrimido[4,5-*b*]indol-4-yl)(methyl)amino)piperidin-1-yl)oxetan-3-yl)acetonitrile (**41**)

**7c** (60.0 mg, 0.19 mmol) and 2-(oxetan-3-ylidene)acetonitrile (36.1 mg, 0.38 mmol) were stirred in EtOH at 70 °C for 6 d. The mixture was concentrated under reduced pressure. Purification of the residue by flash column chromatography (SiO_2_, DCM–MeOH gradient elution from 96:4 to 93.5:6.5) gave 59 mg of a beige solid (76% yield). ^1^H-NMR (400 MHz, DMSO-*d6*) δ 12.21 (s, 1H), 8.41 (s, 1H), 7.79 (d, *J* = 8.6 Hz, 1H), 7.48 (d, *J* = 1.5 Hz, 1H), 7.27 (dd, *J* = 8.6, 1.6 Hz, 1H), 4.52–4.31 (m, 5H), 3.17 (s, 3H), 3.01 (s, 2H), 2.82–2.73 (m, 1H), 2.61–2.54 (m, 1H), 2.49–2.44 (m, 1H, overlap with DMSO-*d5* signal), 2.18–2.06 (m, 1H), 1.93–1.69 (m, 3H), 1.60–1.47 (m, 1H); ^13^C NMR (50 MHz, DMSO-*d6*) δ 159.5, 157.5, 153.8, 137.4, 129.2, 123.8, 120.3, 119.3, 118.5, 110.9, 97.1, 78.1, 61.0, 55.1, 48.0, 45.0, 33.1, 27.3, 24.7, 17.4; ESI-MS: (*m*/*z*) 411.0 [M + H]^+^, 433.0 [M + Na]^+^, 409.0 [M − H]^−^; HPLC method A: t_r_ = 7.888 min.

3-(3-(methyl(9*H*-pyrimido[4,5-*b*]indol-4-yl)amino)piperidin-1-yl)-3-oxopropanenitrile (**18**)

Cyanoacetic acid (36.7 mg, 0.31 mmol) and PyBOP (179.8 mg, 0.34 mmol) were stirred in dry DCM (3 mL) at rt for 20 min. A solution of **7a** (80.4 mg, 0.29 mmol) and DIPEA (43.9 mg, 0.34 mmol) in dry DCM (2 mL) was drop-added. The mixture was stirred at rt for 2 h and then concentrated under reduced pressure. Saturated NaHCO_3_ solution was added to the residue, and the mixture was extracted with EtOAc (3 × 10 mL). Combined organic layers were dried over Na_2_SO_4_ and concentrated under reduced pressure. Purification of the residue by flash column chromatography (SiO_2_, DCM–EtOH gradient elution from 98:2 to 9:1) gave 55 mg (55% yield). ^1^H-NMR shows a 3:2 mixture of amide bond rotamers: (300 MHz, DMSO-*d_6_*) δ 12.19–11.99 (m, 1H), 8.47–8.36 (m, 1H), 7.90–7.78 (m, 1H), 7.54–7.45 (m, 1H), 7.44–7.34 (m, 1H), 7.31–7.19 (m, 1H), 4.55–4.43 (m, 0.6H), 4.39–4.21 (m, 1.4H), 4.18–3.97 (m, 2H), 3.94–3.84 (m, 0.4H), 3.69–3.56 (m, 0.6H), 3.31–3.14 (m, 3.4H), 3.10–2.93 (m, 1.2H), 2.70–2.56 (m, 0.4H), 2.10–1.74 (m, 3H), 1.66–1.42 (m, 1H); ^13^C NMR (101 MHz, DMSO-*d_6_*) δ 161.6, 161.4, 159.6, 159.5, 157.0, 153.3, 136.7, 124.8, 122.7, 122.6, 120.4, 120.3, 119.32, 119.28, 116.1, 116.0, 111.2, 97.9, 97.7, 54.6, 54.2, 47.4, 45.5, 44.2, 42.1, 34.0, 32.7, 27.1, 26.9, 24.8, 24.7, 24.1; ESI-MS: (*m*/*z*) 370.9 [M + Na]^+^, 346.9 [M − H]^−^; HPLC method B: t_r_ = 3.316 min.

3-(3-((7-Fluoro-9*H*-pyrimido[4,5-*b*]indol-4-yl)(methyl)amino)piperidin-1-yl)-3-oxopropanenitrile (**19**)

**7b** (80.0 mg, 0.27 mmol) and DIPEA (51.7 mg, 0.40 mmol) were stirred in dry DCM (3 mL). A suspension of cyanoacetic acid (25.0 mg, 0.29 mmol) and PyBOP (166.9 mg, 0.32 mmol) in dry DCM (3 mL) was drop-added. The mixture was stirred at rt for 2 h and then concentrated under reduced pressure. Saturated NaHCO_3_ solution (10 mL) was added to the residue, and the mixture was extracted with EtOAc (3 × 10 mL). Combined organic layers were dried over Na_2_SO_4_ and concentrated under reduced pressure. Purification of the residue by flash column chromatography (SiO_2_, DCM–EtOH gradient elution from 98:2 to 90:10 (twice)) gave 28 mg (29% yield); ^1^H-NMR shows a 5:4 mixture of amide bond rotamers. ^1^H-NMR (300 MHz, DMSO-*d_6_*) δ 12.28–12.15 (m, 1H), 8.45–8.37 (m, 1H), 7.90–7.77 (m, 1H), 7.30–7.21 (m, 1H), 7.16–7.01 (m, 1H), 4.53–4.44 (m, 0.55H), 4.40–4.17 (m, 1.45H), 4.15–3.98 (m, 2H), 3.92–3.84 (m, 0.45H), 3.66–3.58 (m, 0.55H), 3.30–3.16 (m, 3H), 3.09–2.96 (m, 1.1H), 2.69–2.57 (m, 0.45H), 2.09–1.89 (m, 2H), 1.88–1.75 (m, 1H), 1.67–1.41 (m, 1H), missing 0.45H below water signal; ESI-MS: (*m*/*z*) 389.2 [M + Na]^+^, 365.1 [M − H]^−^; HPLC method B: t_r_ = 4.514 min.

3-(3-((7-bromo-9*H*-pyrimido[4,5-*b*]indol-4-yl)(methyl)amino)piperidin-1-yl)-3-oxopropanenitrile (**20**)

Cyanoacetic acid (31.8 mg, 0.37 mmol) and PyBOP (213.3 mg, 0.4 mmol) were stirred in dry DCM (5 mL) at rt. A suspension of **7d** (120.0 mg, 0.33 mmol) and DIPEA (66.0 mg, 0.5 mmol) in dry DCM (5 mL) was added. The mixture was stirred at rt for 2 h and then concentrated under reduced pressure. Saturated NaHCO_3_ solution was added to the residue, and the mixture was extracted with EtOAc (3 × 10 mL). Combined organic layers were dried over Na_2_SO_4_ and concentrated under reduced pressure. Purification of the residue by flash column chromatography (SiO_2_, DCM–EtOH gradient elution from 98:2 to 9:1) gave 72 mg (51% yield). ^1^H-NMR shows a 5:4 mixture of amide bond rotamers. ^1^H-NMR (300 MHz, DMSO-*d_6_*) δ 12.29–12.17 (m, 1H), 8.47–8.38 (m, 1H), 7.82–7.73 (m, 1H), 7.65–7.59 (m, 1H), 7.43–7.33 (m, 1H), 4.54–4.45 (m, 0.55H), 4.40–4.18 (m, 1.45H), 4.14–3.98 (m, 2H), 3.92–3.82 (m, 0.45H), 3.68–3.57 (m, 0.55H), 3.29–3.17 (m, 3H), 3.09–2.95 (m, 1.1H), 2.70–2.57 (m, 0.45H), 2.08–1.74 (m, 3H), 1.67–1.41 (m, 1H), missing 0.45H below water signal; ESI-MS: (*m*/*z*) 448.9 [M + Na]^+^, 424.8 [M − H]^−^; HPLC method B: t_r_ = 6.305 min.

3-(3-((7-Iodo-9*H*-pyrimido[4,5-*b*]indol-4-yl)(methyl)amino)piperidin-1-yl)-3-oxopropanenitrile (**21**)

The title compound was prepared from **7e** (75 mg, 0.18 mmol), cyanoacetic acid (19.6 mg, 0.23 mmol), TBTU (73.9 mg, 0.23 mmol), and DIPEA (71.4 mg, 0.55 mmol) in dry DCM (total amount 12 mL) according to general procedure F (reaction time 2 h). Purification by flash column chromatography (SiO_2_, DCM–MeOH 96:4) gave 61 mg of a white solid (70% yield); ^1^H-NMR shows a 3:2 mixture of amide bond rotamers. ^1^H-NMR (400 MHz, DMSO-*d_6_*) δ 12.24–12.12 (m, 1H), 8.47–8.38 (m, 1H), 7.79 (s, 1H), 7.68–7.59 (m, 1H), 7.58–7.47 (m, 1H), 4.55–4.42 (m, 0.6H), 4.40–4.19 (m, 1.4H), 4.19–3.98 (m, 2H), 3.92–3.82 (m, 0.4H), 3.69–3.56 (m, 0.6H), 3.32–3.13 (m, 3H), 3.09–2.95 (m, 1.2H), 2.68–2.57 (m, 0.4H), 2.09–1.89 (m, 2H), 1.89–1.75 (m, 1H), 1.67–1.44 (m, 1H), missing 0.4H below water signal; ^13^C NMR (101 MHz, DMSO-*d_6_*) δ 161.6, 161.5, 159.5, 159.4, 156.9, 153.8, 138.0, 128.8, 128.7, 124.6, 124.5, 119.6, 119.02, 118.96, 116.1, 116.0, 97.4, 97.2, 89.5, 89.4, 54.6, 54.2, 47.3, 45.5, 44.1, 42.0, 34.0, 32.7, 27.0, 26.9, 24.8, 24.6, 24.1. ESI-MS: (*m*/*z*) 497.2 [M + Na]^+^, 473.2 [M − H]^−^; HPLC method A: t_r_ = 7.258 min.

3-(3-((7-methoxy-9*H*-pyrimido[4,5-*b*]indol-4-yl)(methyl)amino)piperidin-1-yl)-3-oxopropanenitrile (**22**)

**7f** (120.0 mg, 0.39 mmol) and DIPEA (99.6 mg, 0.77 mmol) were stirred in dry DCM (5 mL) at rt. A suspension of cyanoacetic acid (36.1 mg, 0.42 mmol) and PyBOP (240.7 mg, 0.46 mmol) in dry DCM (5 mL) was drop-added. The mixture was stirred at rt for 2 h and then concentrated under reduced pressure. Saturated NaHCO_3_ solution (10 mL) was added to the residue, and the mixture was extracted with EtOAc (3 × 10 mL). Combined organic layers were dried over Na_2_SO_4_ and concentrated under reduced pressure. Purification of the residue by flash column chromatography (SiO_2_, DCM–EtOH gradient elution from 98:2 to 9:1 (twice)) gave 62 mg (43% yield). ^1^H-NMR shows a 3:2 mixture of amide bond rotamers. ^1^H-NMR (300 MHz, DMSO-*d_6_*) δ 12.05–11.88 (m, 1H), 8.43–8.28 (m, 1H), 7.79–7.61 (m, 1H), 7.02–6.92 (m, 1H), 6.92–6.79 (m, 1H), 4.52–4.40 (m, 0.6H), 4.38–4.16 (m, 1.4H), 4.15–3.97 (m, 2H), 3.93–3.77 (m, 3.4H), 3.68–3.56 (m, 0.6H), 3.27–3.12 (m, 3H), 3.08–2.93 (m, 1.2H), 2.69–2.58 (m, 0.4H), 2.10–1.74 (m, 3H), 1.65–1.41 (m, 1H), missing 0.4H below water signal; ESI-MS: (*m*/*z*) 379.0 [M + H]^+^, 400.9 [M + Na]^+^, 378.0 [M − H]^−^; HPLC method B: t_r_ = 3.635 min.

3-(3-(Methyl(7-(trifluoromethyl)-9*H*-pyrimido[4,5-*b*]indol-4-yl)amino)piperidin-1-yl)-3-oxopropanenitrile (**23**)

**7g** (100.0 mg, 0.29 mmol) and DIPEA (54.3 mg, 0.42 mmol) were stirred in dry DCM (5 mL) at rt. A suspension of cyanoacetic acid (26.8 mg, 0.31 mmol) and PyBOP (178.7 mg, 0.36 mmol) in dry DCM (5 mL) was drop-added. The mixture was stirred at rt for 2 h and then concentrated under reduced pressure. Saturated NaHCO_3_ solution (10 mL) was added to the residue, and the mixture was extracted with EtOAc (3 × 10 mL). Combined organic layers were dried over Na_2_SO_4_ and concentrated under reduced pressure. Purification of the residue twice by flash column chromatography (SiO_2_, DCM–EtOH gradient elution from 97:3 to 4:1 and SiO_2_, DCM–(2N NH_3_ in MeOH) gradient elution from 99:1 to 92:8) gave 28 mg (23% yield); ^1^H-NMR shows a 3:2 mixture of amide bond rotamers. ^1^H-NMR (300 MHz, DMSO-*d_6_*) δ 12.45 (s, 1H), 8.50–8.43 (m, 1H), 8.09–7.99 (m, 1H), 7.74 (s, 1H), 7.59–7.48 (m, 1H), 4.58–4.49 (m, 0.6H), 4.41–4.27 (m, 1.4H), 4.16–4.00 (m, 2H), 3.93–3.84 (m, 0.4H), 3.71–3.60 (m, 0.6H), 3.33–3.21 (m, 3.4H), 3.10–2.95 (m, 1.2H), 2.69–2.58 (m, 0.4H), 2.11–1.76 (m, 3H), 1.70–1.45 (m, 1H); ESI-MS: (*m*/*z*) 438.9 [M + Na]^+^, 415.0 [M − H]^−^; HPLC method B: t_r_ = 6.640 min.

3-(3-((7-Chloro-2-methyl-9*H*-pyrimido[4,5-*b*]indol-4-yl)(methyl)amino)piperidin-1-yl)-3-oxopropanenitrile (**24**)

The title compound was prepared from **7l** (65.0 mg, 0.20 mmol), cyanoacetic acid (21.0 mg, 0.25 mmol), TBTU (79.1 mg, 0.25 mmol), and DIPEA (76.4 mg, 0.59 mmol) in dry DCM (total amount 10 mL) according to general procedure F (reaction time 2 h). Purification by flash column chromatography (SiO_2_, DCM–MeOH gradient elution from 95.5:4.5 to 93:7) gave 48 mg of a grey-yellow solid (61% yield). ^1^H-NMR shows a 1:1 mixture of amide bond rotamers. ^1^H-NMR (300 MHz, DMSO-*d_6_*) δ 12.02 (s, 1H), 7.84–7.71 (m, 1H), 7.45 (d, *J* = 2.0 Hz, 1H), 7.28–7.15 (m, 1H), 4.57–4.48 (m, 0.5H), 4.39–4.30 (m, 0.5H), 4.29–3.97 (m, 3H), 3.95–3.86 (m, 0.5H), 3.67–3.58 (m, 0.5H), 3.34–3.27 (m, 0.5H), 3.26–3.14 (m, 3H), 3.08–2.92 (m, 1H), 2.70–2.58 (m, 0.5H), 2.53–2.47 (m, 3H, overlap with DMSO-*d_5_* signal), 2.13–1.72 (m, 3H), 1.67–1.43 (m, 1H); ^13^C NMR (101 MHz, DMSO-*d_6_*) δ 162.6, 162.5, 161.7, 161.5, 159.5, 159.4, 158.5, 158.4, 137.5, 137.4, 128.8, 123.7, 123.5, 120.3, 118.6, 118.5, 116.2, 116.0, 110.8, 95.2, 95.0, 54.5, 54.3, 47.2, 45.6, 44.1, 42.3, 34.2, 32.7, 27.4, 27.0, 25.8, 25.7, 25.0, 24.9, 24.7, 24.3; ESI-MS: (*m*/*z*) 396.9 [M + H]^+^, 418.9 [M + Na]^+^, 394.9 [M − H]^−^; HPLC method A: t_r_ = 7.427 min.

1-(3-(methyl(9*H*-pyrimido[4,5-*b*]indol-4-yl)amino)piperidin-1-yl)propan-1-one (**25**)

The title compound was prepared from **7a** (60.0 mg, 0.21 mmol), propionic acid (19.8 mg, 0.27 mmol), TBTU (85.6 mg, 0.27 mmol), and DIPEA (82.7 mg, 0.64 mmol) in dry DCM (total amount 10 mL) according to general procedure F (reaction time 1 h). Purification by flash column chromatography (SiO_2_; DCM–MeOH 94:6) gave 45 mg of a beige solid (63% yield). ^1^H-NMR shows a 5:4 mixture of amide bond rotamers. ^1^H-NMR (400 MHz, DMSO-*d_6_*) δ 12.08 (s, 1H), 8.43–8.36 (m, 1H), 7.88–7.78 (m, 1H), 7.52–7.45 (m, 1H), 7.43–7.35 (m, 1H), 7.30–7.17 (m, 1H), 4.62–4.49 (m, 0.55H), 4.45–4.37 (m, 0.45H), 4.35–4.27 (m, 0.45H), 4.26–4.16 (m, 0.55H), 4.08–4.00 (m, 0.45H), 3.88–3.75 (m, 0.55H), 3.29–3.15 (m, 3.45H), 3.02–2.87 (m, 1.1H), 2.56–2.46 (m, 0.45H, overlap with DMSO-*d_6_* signal), 2.46–2.26 (m, 2H), 2.07–1.87 (m, 2H), 1.85–1.75 (m, 1H), 1.52–1.37 (m, 1H), 1.02–0.92 (m, 3H); ESI-MS: (*m*/*z*) 338.7 [M + H]^+^, 360.7 [M + Na]^+^, 336.7 [M − H]^−^; HPLC method A: t_r_ = 6.107 min.

1-(3-((7-Fluoro-9*H*-pyrimido[4,5-*b*]indol-4-yl)(methyl)amino)piperidin-1-yl)propan-1-one (**26**)

The title compound was prepared from **7b** (50.0 mg, 0.17 mmol), propionic acid (15.5 mg, 0.21 mmol), TBTU (67.5 mg, 0.21 mmol), and DIPEA (64.8 mg, 0.50 mmol) in dry DCM (total amount 15 mL) according to general procedure F (reaction time 45 min). Purification by flash column chromatography (SiO_2_; DCM–MeOH 95:5) gave 50 mg of a white solid (84% yield). ^1^H-NMR (400 MHz, DMSO-*d_6_*) δ 12.28–12.14 (m, 1H), 8.44–8.34 (m, 1H), 7.86–7.76 (m, 1H), 7.30–7.20 (m, 1H), 7.13–6.99 (m, 1H), 4.60–4.51 (m, 0.55H), 4.45–4.36 (m, 0.45H), 4.32–4.23 (m, 0.45H), 4.21–4.11 (m, 0.55H), 4.08–3.98 (m, 0.45H), 3.87–3.74 (m, 0.55H), 3.28–3.14 (m, 3.45H), 3.00–2.85 (m, 1.1H), 2.54–2.46 (m, 0.45H, overlap with DMSO-*d_5_* signal), 2.44–2.26 (m, 2H), 2.05–1.86 (m, 2H), 1.83–1.75 (m, 1H), 1.52–1.37 (m, 1H), 1.03–0.92 (m, 3H); ^13^C NMR (101 MHz, DMSO-*d_6_*) δ 171.54, 171.37, 160.25 (d, *J* = 240.1 Hz), 159.33, 159.21, 157.72, 153.12, 153.03, 137.47 (d, *J* = 12.5 Hz), 124.14–123.87 (m), 116.17, 108.07 (d, *J* = 23.5 Hz), 97.80 (d, *J* = 26.1 Hz), 54.80, 54.55, 46.74, 44.79, 43.87, 41.22, 33.75, 32.55, 27.52, 27.24, 25.65, 25.15, 24.46, 9.43. ESI-MS: (*m*/*z*) 378.3 [M + Na]^+^, 354.4 [M − H]^−^; HPLC method B: t_r_ = 7.075 min.

1-(3-((7-Chloro-2-methyl-9*H*-pyrimido[4,5-*b*]indol-4-yl)(methyl)amino)piperidin-1-yl)propan-1-one (**27**)

The title compound was prepared from **7l** (28.0 mg, 0.09 mmol), propionic acid (7.9 mg, 0.11 mmol), TBTU (34.1 mg, 0.11 mmol), and DIPEA (32.9 mg, 0.26 mmol) in dry DCM (total amount 5 mL) according to general procedure F (reaction time 2 h). Purification by flash column chromatography (SiO_2_, DCM–MeOH gradient elution from 96:4 to 94:6) gave 20 mg of a beige solid (61% yield). ^1^H-NMR shows a 3:2 mixture of amide bond rotamers. ^1^H-NMR (400 MHz, DMSO-*d_6_*) δ 12.02 (s, 1H), 7.85–7.71 (m, 1H), 7.48–7.40 (m, 1H), 7.29–7.13 (m, 1H), 4.64–4.53 (m, 0.4H), 4.49–4.37 (m, 0.6H), 4.34–4.23 (m, 0.6H), 4.20–4.06 (m, 1H), 3.89–3.77 (m, 0.4H), 3.29–3.14 (m, 3.6H), 3.02–2.86 (m, 0.8H), 2.63–2.54 (m, 0.6H), 2.47–2.29 (m, 2H), 2.10–1.75 (m, 3H), 1.54–1.39 (m, 1H), 1.08–0.92 (m, 3H); ESI-MS: (*m*/*z*) 408.2 [M + Na]^+^, 384.2 [M − H]^−^; HPLC method A: t_r_ = 7.761 min.

1-(3-((7-Bromo-9*H*-pyrimido[4,5-*b*]indol-4-yl)(methyl)amino)piperidin-1-yl)propan-1-one (**28**)

The title compound was prepared from **7d** (115.0 mg, 0.32 mmol), propionic acid (29.6 mg, 0.40 mmol), TBTU (128.1 mg, 0.40 mmol), and DIPEA (123.8 mg, 0.96 mmol) in dry DCM (total amount 15 mL) according to general procedure F (reaction time 30 min). Purification by flash column chromatography (SiO_2_, DCM–MeOH 94:6) gave 48 mg of an off-white solid (36% yield). ^1^H-NMR shows a 5:4 mixture of amide bond rotamers. ^1^H-NMR (400 MHz, CDCl_3_) δ 12.75–12.17 (m, 1H), 8.58–8.47 (m, 1H), 7.67–7.53 (m, 2H), 7.40–7.30 (m, 1H), 5.01–4.90 (m, 0.45H), 4.80–4.68 (m, 0.55H), 4.54–4.40 (m, 0.55H), 4.38–4.29 (m, 0.45H), 4.28–4.20 (m, 0.55H), 3.95–3.82 (m, 0.45H), 3.36–3.20 (m, 3H), 3.19–3.09 (m, 0.55H), 3.05–2.88 (m, 0.9H), 2.67–2.36 (m, 2.55H), 2.21–1.86 (m, 3H), 1.76–1.56 (m, 1H), 1.28–1.13 (m, 3H); ^13^C NMR (101 MHz, CDCl_3_) δ 173.2, 172.6, 160.1, 160.0, 157.54, 157.46, 153.0, 137.9, 124.0, 123.92, 123.88, 119.1, 119.0, 118.73, 118.68, 114.6, 114.4, 98.4, 55.2, 55.0, 47.5, 45.8, 45.0, 42.2, 34.4, 33.4, 28.8, 28.0, 26.9, 26.8, 25.6, 24.9, 9.8; ESI-MS: (*m*/*z*) 438.0 [M + Na]^+^, 414.0 [M − H]^−^; HPLC method A: t_r_ = 8.212 min.

1-(3-((7-Iodo-9*H*-pyrimido[4,5-*b*]indol-4-yl)(methyl)amino)piperidin-1-yl)propan-1-one (**29**)

The title compound was prepared from **7e** (43.0 mg, 0.11 mmol), propionic acid (9.8 mg, 0.13 mmol), TBTU (42.4 mg, 0.13 mmol), and DIPEA (40.9 mg, 0.32 mmol) in dry DCM (total amount 8 mL) according to general procedure F (reaction time 2 h). Purification by flash column chromatography (SiO_2_, DCM–MeOH 95:5) gave 25 mg of an off-white solid (51% yield). ^1^H-NMR shows a 1:1 mixture of amide bond rotamers. ^1^H-NMR (400 MHz, DMSO-*d_6_*) δ 12.20–12.11 (m, 1H), 8.43–8.37 (m, 1H), 7.82–7.76 (m, 1H), 7.67–7.60 (m, 1H), 7.56–7.47 (m, 1H), 4.59–4.52 (m, 0.5H), 4.44–4.37 (m, 0.5H), 4.35–4.26 (m, 0.5H), 4.23–4.14 (m, 0.5H), 4.07–3.98 (m, 0.5H), 3.85–3.79 (m, 0.5H), 3.26–3.15 (m, 3.5H), 3.01–2.87 (m, 1H), 2.54–2.46 (m, 0.5H, overlap with DMSO-*d_5_* signal), 2.45–2.25 (m, 2H), 2.05–1.75 (m, 3H), 1.53–1.37 (m, 1H), 1.02–0.93 (m, 3H); ESI-MS: (*m*/*z*) 464.9 [M + H]^+^, 486.9 [M + Na]^+^, 462.8 [M − H]^−^; HPLC method A: t_r_ = 8.443 min.

3-(3-((6-Chloro-9*H*-pyrimido[4,5-*b*]indol-4-yl)(methyl)amino)piperidin-1-yl)-3-oxopropanenitrile (**30**)

Cyanoacetic acid (16.8 mg, 0.20 mmol) and TBTU (63.5 mg, 0.20 mmol) were stirred in dry DCM (5 mL) at rt and under N_2_ atmosphere for 15 min. A suspension of **7h** (50.0 mg, 0.16 mmol) and DIPEA (61.4 mg, 0.48 mmol) in dry DCM (5 mL) was added to the activated acid, and the mixture was stirred at rt and under N_2_ atmosphere for 2.5 h. A precipitate formed. The mixture was diluted with DCM, and MeOH was added to dissolve the precipitate. The solution was washed with saturated NaHCO_3_ solution (2 × 20 mL) and saturated NH_4_Cl solution (2 × 20 mL). The organic layer was dried over Na_2_SO_4_ and concentrated under reduced pressure. Purification of the residue by flash column chromatography (SiO_2_, DCM–MeOH gradient elution from 95:5 to 92:8) gave 34 mg of a white solid (56% yield); ^1^H-NMR shows a 5:4 mixture of amide rotamers. ^1^H-NMR (400 MHz, DMSO-*d_6_*) δ 12.32–12.18 (m, 1H), 8.46–8.36 (m, 1H), 7.83–7.73 (m, 1H), 7.53–7.45 (m, 1H), 7.44–7.36 (m, 1H), 4.51–4.42 (m, 0.55H), 4.40–4.25 (m, 1.45H), 4.17–3.99 (m, 2H), 3.93–3.85 (m, 0.45H), 3.69–3.60 (m, 0.55H), 3.29–3.19 (m, 3H), 3.07–2.91 (m, 1.1H), 2.68–2.57 (m, 0.45H), 2.10–1.94 (m, 2H), 1.89–1.79 (m, 1H), 1.71–1.42 (m, 1H), missing 0.45H below water signal; ^13^C NMR (101 MHz, DMSO-*d_6_*) δ 161.6, 161.5, 159.5, 157.5, 153.8, 135.20, 135.18, 124.7, 124.6, 121.8, 121.7, 120.72, 120.71, 116.02, 115.97, 112.6, 97.0, 96.9, 54.6, 53.8, 47.4, 45.4, 43.9, 42.0, 34.1, 33.1, 27.0, 26.9, 24.9, 24.6, 24.1; ESI-MS: (*m*/*z*) 405.4 [M + Na]^+^, 381.4 [M − H]^−^; HPLC method A: t_r_ = 6.666 min.

3-(3-((6-Bromo-9*H*-pyrimido[4,5-*b*]indol-4-yl)(methyl)amino)piperidin-1-yl)-3-oxopropanenitrile (**31**)

A mixture of cyanoacetic acid (21.3 mg, 0.25 mmol) and EDCI·HCl (47.9 mg, 0.25 mmol) was stirred in dry DCM (6 mL) at rt and under N_2_ atmosphere for 20 min. A suspension of **7i** (60.0 mg, 0.17 mmol) and DIPEA (64.6 mg, 0.51 mmol) in dry DCM (4 mL) was added, and the mixture was stirred at rt and under N_2_ atmosphere. Due to slow conversion, reactants were added repeatedly: EDCI·HCl (47.9 mg, 0.25 mmol) after 3 h of stirring, cyanoacetic acid (21.3 mg, 0.25 mmol) after 5 h of stirring, and again EDCI·HCl (63.9 mg, 0.33 mmol) after 20 h of stirring. Sufficient conversion was achieved after a reaction time of 2 days. The mixture was diluted with DCM, washed with saturated NH_4_Cl solution and saturated NaHCO_3_ solution, dried over Na_2_SO_4_, and concentrated under reduced pressure. Purification of the residue by flash column chromatography (SiO_2_, DCM–MeOH 95:5) gave 16 mg of an off-white solid (22% yield). ^1^H-NMR shows a 5:4 mixture of amide bond rotamers. ^1^H-NMR (400 MHz, DMSO-*d_6_*) δ 12.34–12.22 (m, 1H), 8.44 (s, 0.45H), 8.40 (s, 0.55H), 7.96–7.87 (m, 1H), 7.57–7.50 (m, 1H), 7.48–7.41 (m, 1H), 4.49–4.41 (m, 0.55H), 4.40–4.24 (m, 1.45H), 4.15–3.99 (m, 2H), 3.92–3.84 (m, 0.45H), 3.70–3.60 (m, 0.55H), 3.29–3.19 (m, 3H), 3.07–2.92 (m, 1.1H), 2.69–2.57 (m, 0.45H), 2.11–1.95 (m, 2H), 1.90–1.80 (m, 1H), 1.72–1.43 (m, 1H), missing 0.45H below water signal; ^13^C NMR (101 MHz, DMSO-*d_6_*) δ 161.6, 161.5, 159.5, 159.4, 157.4, 153.9, 135.5, 127.3, 127.2, 124.7, 124.6, 121.4, 121.3, 116.1, 116.0, 113.1, 112.5, 112.4, 96.8, 96.8, 54.7, 53.9, 47.4, 45.4, 43.9, 42.0, 34.0, 33.1, 27.0, 26.9, 24.9, 24.6, 24.1. ESI-MS: (m/z) 449.4 [M + Na]^+^, 425.5 [M − H]^−^; HPLC method A: t_r_ = 6.749 min.

3-(3-((6-Methoxy-9*H*-pyrimido[4,5-*b*]indol-4-yl)(methyl)amino)piperidin-1-yl)-3-oxopropanenitrile (**32**)

The title compound was prepared from **7j** (60.0 mg, 0.19 mmol), cyanoacetic acid (20.5 mg, 0.24 mmol), TBTU (77.3 mg, 0.24 mmol), and DIPEA (74.7 mg, 0.58 mmol) in dry DCM (total amount 10 mL) according to general procedure F (reaction time 0.5 h). Purification by flash column chromatography (SiO_2_; DCM–MeOH 95:5) gave 38 mg of a beige solid (52% yield). ^1^H-NMR shows a 5:4 mixture of amide bond rotamers. ^1^H-NMR (400 MHz, DMSO-*d_6_*) δ 12.00–11.87 (m, 1H), 8.43–8.36 (m, 1H), 7.44–7.37 (m, 1H), 7.34–7.20 (m, 1H), 7.09–7.01 (m, 1H), 4.40–3.97 (m, 4H), 3.92–3.80 (m, 3.45H), 3.65–3.57 (m, 0.55H), 3.30–3.17 (m, 3H), 3.06–2.92 (m, 1.1H), 2.69–2.57 (m, 0.4H), 2.15–1.98 (m, 2H), 1.90–1.79 (m, 1H), 1.71–1.44 (m, 1H), missing 0.45H below water signal; ^13^C NMR (101 MHz, DMSO-*d_6_*) δ 161.6, 161.4, 159.9, 159.6, 157.3, 157.2, 154.0, 153.9, 153.1, 131.4, 131.3, 119.9, 119.7, 116.00, 115.96, 113.6, 112.9, 111.9, 111.8, 106.8, 106.0, 98.3, 97.9, 55.6, 55.5, 54.7, 54.3, 47.4, 45.4, 44.0, 42.0, 34.0, 32.7, 27.2, 27.1, 24.9, 24.8, 24.6, 24.1; ESI-MS: (*m*/*z*) 379.1 [M + H]^+^, 401.2 [M + Na]^+^, 377.2 [M − H]^−^; HPLC method A: t_r_ = 4.053 min.

3-(3-((5-Chloro-9*H*-pyrimido[4,5-*b*]indol-4-yl)(methyl)amino)piperidin-1-yl)-3-oxopropanenitrile (**33**)

The title compound was prepared from **7k** (55.0 mg, 0.17 mmol), cyanoacetic acid (18.5 mg, 0.22 mmol), TBTU (69.9 mg, 0.22 mmol), and DIPEA (67.5 mg, 0.52 mmol) in dry DCM (total amount 10 mL) according to general procedure F (reaction time 1 h). A precipitate formed during the reaction and was dissolved by adding DCM and MeOH prior to the extractive work-up. Repeated purification by flash column chromatography (SiO_2_, DCM–MeOH 94:6; SiO_2_, DCM–MeOH gradient elution from 96:4 to 92:8; SiO_2_, DCM–MeOH gradient elution from 96:4 to 92:8; and SiO_2_, DCM–EtOH 95:5) gave 35 mg of a beige solid (52% yield). ^1^H-NMR shows a 1:1 mixture of amide bond rotamers. ^1^H-NMR (300 MHz, DMSO-*d_6_*) δ 12.35 (s, 1H), 8.50–8.32 (m, 1H), 7.50–7.34 (m, 2H), 7.33–7.25 (m, 1H), 4.42–3.95 (m, 4H), 3.92–3.80 (m, 0.5H), 3.66–3.55 (m, 0.5H), 3.09–2.89 (m, 4H), 2.71–2.58 (m, 0.5H), 2.07–1.73 (m, 3H), 1.71–1.42 (m, 1H), missing 0.5H below water signal; ESI-MS: (*m*/*z*) 405.4 [M + Na]^+^, 381.3 [M − H]^−^; HPLC method A: t_r_ = 6.396 min.

1-(3-((6-Chloro-9*H*-pyrimido[4,5-*b*]indol-4-yl)(methyl)amino)piperidin-1-yl)propan-1-one (**34**)

The title compound was prepared from **7h** (50.0 mg, 0.16 mmol), propionic acid (14.7 mg, 0.20 mmol), TBTU (63.5 mg, 0.20 mmol), and DIPEA (61.4 mg, 0.48 mmol) in dry DCM (10 mL) according to general procedure F (reaction time 45 min). Purification by flash column chromatography (SiO_2_, DCM–MeOH gradient elution from 96.5:3.5 to 92.5: 7.5) gave 42 mg of an off-white solid (71% yield); ^1^H-NMR shows a 1:1 mixture of amide bond rotamers. ^1^H-NMR (400 MHz, DMSO-*d_6_*) δ 12.25 (s, 1H), 8.39 (s, 1H), 7.84–7.69 (m, 1H), 7.52–7.45 (m, 1H), 7.43–7.37 (m, 1H), 4.55–4.46 (m, 0.5H), 4.46–4.38 (m, 0.5H), 4.35–4.19 (m, 1H), 4.12–4.00 (m, 0.5H), 3.89–3.77 (m, 0.5H), 3.31–3.18 (m, 3.5H), 3.02–2.92 (m, 0.5H), 2.92–2.83 (m, 0.5H), 2.55–2.26 (m, 2.5H, overlap with DMSO-*d_5_* signal), 2.08–1.93 (m, 2H), 1.90–1.75 (m, 1H), 1.60–1.35 (m, 1H), 1.04–0.91 (m, 3H); ^13^C NMR (101 MHz, DMSO-*d_6_*) δ 171.5, 171.4, 159.5, 157.5, 153.8, 153.8, 135.2, 124.7, 124.6, 124.5, 121.8, 121.6, 120.7, 112.6, 96.9, 96.8, 54.8, 54.1, 46.8, 44.7, 43.6, 41.1, 33.8, 32.9, 27.4, 27.2, 25.6, 25.0, 24.4, 9.3; ESI-MS: (*m*/*z*) 394.4 [M + Na]^+^, 370.4 [M − H]^−^; HPLC method A: t_r_ = 7.789 min.

1-(3-((6-Bromo-9*H*-pyrimido[4,5-*b*]indol-4-yl)(methyl)amino)piperidin-1-yl)propan-1-one (**35**)

Propionic acid (16.7 mg, 0.23 mmol) and TBTU (72.4 mg, 0.23 mmol) were stirred in dry DCM (4 mL) at rt and under N_2_ atmosphere for 15 min. A suspension of **7i** (65.0 mg, 0.18 mmol) in dry DCM (8 mL) was added to the activated acid, followed by addition of DIPEA (70.0 mg, 0.54 mmol). The mixture was stirred at rt and under N_2_ atmosphere for 50 min; then diluted with DCM; and washed with saturated NaHCO_3_ solution (2 × 25 mL), saturated NH_4_Cl solution (2 × 25 mL), and saturated NaCl solution (25 mL). The organic layer was dried over Na_2_SO_4_ and concentrated under reduced pressure. Purification of the residue by flash column chromatography (DCM–MeOH 95:5) gave 24 mg of an off-white solid (32% yield); ^1^H-NMR shows a 1:1 mixture of amide bond rotamers. ^1^H-NMR (400 MHz, DMSO-*d_6_*) δ 12.26 (s, 1H), 8.39 (s, 1H), 7.96–7.81 (m, 1H), 7.56–7.48 (m, 1H), 7.48–7.40 (m, 1H), 4.50–4.36 (m, 1H), 4.34–4.16 (m, 1H), 4.08–4.01 (m, 0.5H), 3.87–3.77 (m, 0.5H), 3.31–3.18 (m, 3.5H), 3.02–2.92 (m, 0.5H), 2.92–2.82 (m, 0.5H), 2.54–2.26 (m, 2.5H, overlap with DMSO-*d_5_* signal), 2.09–1.96 (m, 2H), 1.92–1.76 (m, 1H), 1.60–1.36 (m, 1H), 1.02–0.90 (m, 3H); ^13^C NMR (101 MHz, DMSO-*d_6_*) δ 171.6, 171.5, 159.5, 157.4, 153.9, 153.8, 135.5, 127.3, 124.7, 124.5, 121.4, 113.2, 112.5, 96.8, 96.7, 54.9, 54.1, 46.9, 44.7, 43.7, 41.2, 33.8, 32.9, 27.4, 27.3, 25.7, 25.1, 24.4, 9.4; ESI-MS: (*m*/*z*) 438.0 [M + Na]^+^, 414.0 [M − H]^−^; HPLC method A: t_r_ = 8.360 min.

1-(3-((6-Methoxy-9*H*-pyrimido[4,5-*b*]indol-4-yl)(methyl)amino)piperidin-1-yl)propan-1-one (**36**)

The title compound was prepared from **7j** (60.0 mg, 0.19 mmol), cyanoacetic acid (17.9 mg, 0.24 mmol), TBTU (77.3 mg, 0.24 mmol), and DIPEA (74.7 mg, 0.58 mmol) in dry DCM (total amount 10 mL) according to general procedure F (reaction time 2 h). Purification by flash column chromatography (SiO_2_; DCM–MeOH 95:5) gave 43 mg of a beige solid (61% yield). ^1^H-NMR (300 MHz, DMSO-*d_6_*) δ 12.03–11.84 (m, 1H), 8.45–8.31 (m, 1H), 7.46–7.36 (m, 1H), 7.33–7.19 (m, 1H), 7.12–6.99 (m, 1H), 4.50–4.34 (m, 1H), 4.33–4.09 (m, 1H), 4.06–3.96 (m, 0.45H), 3.90–3.74 (m, 3.55H), 3.30–3.14 (m, 3.45H), 3.03–2.81 (m, 1.1H), 2.57–2.44 (m, 0.45H, overlap with DMSO-*d_5_* signal), 2.41–2.23 (m, 2H), 2.12–1.77 (m, 3H), 1.62–1.37 (m, 1H), 1.01–0.86 (m, 3H); ^13^C NMR (101 MHz, DMSO-*d_6_*) δ 171.42, 171.38, 159.9, 159.6, 157.3, 157.2, 154.0, 153.9, 153.1, 131.3, 119.8, 119.8, 113.5, 113.0, 111.9, 111.8, 106.7, 105.9, 98.3, 97.8, 55.6, 55.5, 54.9, 54.5, 46.8, 44.6, 43.6, 41.2, 33.6, 32.6, 27.6, 27.3, 25.6, 25.1, 24.4, 9.3; ESI-MS: (*m*/*z*) 390.2 [M + Na]^+^, 366.2 [M − H]^−^; HPLC method A: t_r_ = 5.463 min.

1-(3-((5-Chloro-9*H*-pyrimido[4,5-*b*]indol-4-yl)(methyl)amino)piperidin-1-yl)propan-1-one (**37**)

The title compound was prepared from **7k** (55.0 mg, 0.17 mmol), propionic acid (16.2 mg, 0.22 mmol), TBTU (69.9 mg, 0.22 mmol), and DIPEA (67.5 mg, 0.52 mmol) in dry DCM (total amount 10 mL) according to general procedure F (reaction time 2 h). Purification by flash column chromatography (SiO_2_, DCM–MeOH 94:6) gave 46 mg of a light beige solid (71% yield). NMR shows a 5:4 mixture of amide rotamers. ^1^H-NMR (300 MHz, Pyr-*d_5_*) δ 14.06–13.63 (m, 1H), 8.88–8.80 (m, 1H), 7.70–7.61 (m, 1H), 7.50–7.36 (m, 2H), 4.87–4.77 (m, 0.55H), 4.69–4.55 (m, 0.45H), 4.55–4.42 (m, 0.55H), 4.27–4.15 (m, 0.55H), 3.73–3.58 (m, 0.45H), 3.23–2.95 (m, 4H), 2.95–2.78 (m, 0.45H), 2.72–2.22 (m, 2.55H), 2.17–1.96 (m, 1H), 1.95–1.42 (m, 3H), 1.31–1.11 (m, 3H), missing 0.45H below water signal; ESI-MS: (*m*/*z*) 372.3 [M + H]^+^, 394.4 [M + Na]^+^, 370.3 [M − H]^−^; HPLC method A: t_r_ = 7.646 min.

##### (8) Detailed Procedures for the Preparation of Enantiopure Final Compounds ***(R)*-2**, ***(R)*-20** and ***(R)*-28**

*(R)*-3-(3-((7-Chloro-9*H*-pyrimido[4,5-*b*]indol-4-yl)(methyl)amino)piperidin-1-yl)-3-oxopropanenitrile (***(R)*-2**)

The title compound was prepared from ***(R)*-7c** (50.0 mg, 0.16 mmol), cyanoacetic acid (16.2 mg, 0.19 mmol), PyBOP (98.9 mg, 0.19 mmol), and DIPEA (61.4 mg, 0.48 mmol) in dry DCM (total amount 10 mL) according to general procedure F (reaction time 2 h). Purification twice by flash column chromatography (SiO_2_, DCM–MeOH gradient elution from 1:0 to 92:8 and SiO_2_, DCM–EtOH gradient elution from 96.5:3.5 to 92:8) gave 33 mg of a white solid (54% yield). ^1^H-NMR shows a 3:2 mixture of amide bond rotamers. ^1^H-NMR (400 MHz, DMSO-*d_6_*) δ 12.33–12.17 (m, 1H), 8.48–8.36 (m, 1H), 7.90–7.77 (m, 1H), 7.53–7.45 (m, 1H), 7.31–7.18 (m, 1H), 4.55–4.46 (m, 0.6H), 4.39–4.20 (m, 1.4H), 4.18–4.00 (m, 2H), 3.94–3.83 (m, 0.4H), 3.69–3.58 (m, 0.6H), 3.29–3.17 (m, 3H), 3.09–2.97 (m, 1.2H), 2.70–2.58 (m, 0.4H), 2.12–1.74 (m, 3H), 1.68–1.43 (m, 1H), missing 0.4H below water signal; ^13^C NMR (101 MHz, DMSO-*d_6_*) δ 161.6, 161.5, 159.4, 159.3, 157.5, 153.7, 137.4, 129.3, 129.2, 124.1, 124.0, 120.4, 118.4, 118.3, 116.14, 116.08, 110.8, 97.3, 97.2, 54.6, 54.2, 47.3, 45.5, 44.1, 42.0, 34.1, 32.8, 27.0, 26.9, 24.9, 24.7, 24.2; ESI-MS: (*m*/*z*) 404.9 [M + Na]^+^, 380.8 [M − H]^−^; HPLC method A: t_r_ = 6.644 min.

*(R)*-3-(3-((7-Bromo-9*H*-pyrimido[4,5-*b*]indol-4-yl)(methyl)amino)piperidin-1-yl)-3-oxopropanenitrile (***(R)*-20**)

The title compound was prepared from ***(R)*-7d** (70.0 mg, 0.19 mmol), cyanoacetic acid (20.7 mg, 0.24 mmol), TBTU (78.0 mg, 0.24 mmol), and DIPEA (75.3 mg, 0.58 mmol) in dry DCM (total amount 12 mL) according to general procedure F (reaction time 30 min). Purification by flash column chromatography (SiO_2_, DCM–MeOH 94.5:5.5) gave 63 mg of a white solid (76% yield); ^1^H-NMR shows a 3:2 mixture of amide bond rotamers. ^1^H-NMR (300 MHz, DMSO-*d_6_*) δ 12.30–12.14 (m, 1H), 8.49–8.37 (m, 1H), 7.82–7.72 (m, 1H), 7.66–7.58 (m, 1H), 7.44–7.31 (m, 1H), 4.55–4.44 (m, 0.6H), 4.40–4.19 (m, 1.4H), 4.16–3.98 (m, 2H), 3.93–3.82 (m, 0.4H), 3.68–3.57 (m, 0.6H), 3.28–3.14 (m, 3.4H), 3.09–2.94 (m, 1.2H), 2.70–2.56 (m, 0.4H), 2.09–1.74 (m, 3H), 1.67–1.41 (m, 1H); ^13^C NMR (101 MHz, DMSO-*d_6_*) δ 161.6, 161.4, 159.44, 159.36, 157.3, 153.7, 137.7, 124.4, 124.2, 123.1, 123.0, 118.7, 118.6, 117.38, 117.35, 116.1, 116.0, 113.7, 97.3, 97.2, 54.6, 54.2, 47.3, 45.5, 44.1, 42.0, 34.0, 32.8, 27.0, 26.9, 24.8, 24.6, 24.1; ESI-MS: (*m*/*z*) 449.4 [M + Na]^+^, 425.5 [M − H]^−^; HPLC method A: t_r_ = 6.672 min.

*(R)*-1-(3-((7-bromo-9*H*-pyrimido[4,5-*b*]indol-4-yl)(methyl)amino)piperidin-1-yl)propan-1-one (***(R)*-28**)

The title compound was prepared from ***(R)*-7d** (45.0 mg, 0.13 mmol), propionic acid (11.6 mg, 0.16 mmol), TBTU (50.1 mg, 0.16 mmol), and DIPEA (48.4 mg, 0.38 mmol) in dry DCM (total amount 10 mL) according to general procedure F (reaction time 40 min). Purification by flash column chromatography (SiO_2_, DCM–MeOH gradient elution from 96:4 to 92:8) gave 28 mg (54% yield). ^1^H-NMR shows a 5:4 mixture of amide bond rotamers. ^1^H-NMR (400 MHz, CDCl_3_) δ 12.89–12.09 (m, 1H), 8.52 (s, 1H), 7.73–7.55 (m, 2H), 7.43–7.29 (m, 1H), 5.01–4.88 (m, 0.45H), 4.80–4.67 (m, 0.55H), 4.55–4.41 (m, 0.55H), 4.39–4.29 (m, 0.45H), 4.28–4.18 (m, 0.55H), 3.94–3.80 (m, 0.45H), 3.35–3.19 (m, 3H), 3.18–3.09 (m, 0.55H), 3.04–2.87 (m, 0.9H), 2.64–2.35 (m, 2.55H), 2.20–1.84 (m, 3H), 1.75–1.54 (m, 1H), 1.26–1.17 (m, 3H); ^13^C NMR (101 MHz, CDCl_3_) δ 173.2, 172.6, 160.1, 157.6, 157.4, 153.0, 137.84, 137.76, 124.1, 124.03, 123.96, 119.2, 119.1, 118.84, 118.79, 114.6, 114.4, 98.8, 98.5, 55.2, 55.1, 47.5, 45.8, 45.0, 42.3, 34.5, 33.4, 29.8, 28.8, 28.0, 26.9, 25.6, 24.9, 9.8; ESI-MS: (*m*/*z*) 416.0 [M + H]^+^, 438.0 [M + Na]^+^, 414.0 [M − H]^−^; HPLC method A: t_r_ = 8.046 min.

##### (9) Detailed Procedures for the Preparation of Enantiopure Final Compounds ***(S)*-2** and ***(S)*-20**

*(S)*-3-(3-((7-Chloro-9*H*-pyrimido[4,5-*b*]indol-4-yl)(methyl)amino)piperidin-1-yl)-3-oxopropanenitrile (***(S)*-2**)

The title compound was prepared from ***(S)*-7c** (64.0 mg, 0.20 mmol), cyanoacetic acid (20.7 mg, 0.24 mmol), PyBOP (126.6 mg, 0.24 mmol), and DIPEA (78.6 mg, 0.61 mmol) in dry DCM (total amount 10 mL) according to general procedure F (reaction time 3 h). Purification by flash column chromatography (SiO_2_, DCM–MeOH gradient elution from 95.5:4.5 to 92:8) gave 54 mg of a white solid (70% yield). ^1^H-NMR shows a 3:2 mixture of amide bond rotamers: (300 MHz, DMSO-*d_6_*) δ 12.31–12.16 (m, 1H), 8.48–8.37 (m, 1H), 7.89–7.77 (m, 1H), 7.53–7.43 (m, 1H), 7.32–7.18 (m, 1H), 4.58–4.44 (m, 0.6H), 4.41–4.19 (m, 1.4H), 4.16–3.98 (m, 2H), 3.93–3.81 (m, 0.4H), 3.71–3.56 (m, 0.6H), 3.29–3.17 (m, 3H), 3.10–2.94 (m, 1.2H), 2.70–2.56 (m, 0.4H), 2.10–1.73 (m, 3H), 1.68–1.42 (m, 1H), missing 0.4H below water signal; ESI-MS: (*m*/*z*) 405.1 [M + Na]^+^, 380.9 [M − H]^−^; HPLC method A: t_r_ = 6.679 min.

*(S)*-3-(3-((7-Bromo-9*H*-pyrimido[4,5-*b*]indol-4-yl)(methyl)amino)piperidin-1-yl)-3-oxopropanenitrile (***(S)*-20**)

The title compound was prepared from ***(S)*-7d** (65.0 mg, 0.18 mmol), cyanoacetic acid (19.2 mg, 0.23 mmol), TBTU (72.4 mg, 0.23 mmol), and DIPEA (70.0 mg, 0.54 mmol) in dry DCM (total amount 12 mL) according to general procedure F (reaction time 30 min). Purification by flash column chromatography (SiO_2_, DCM–MeOH 94.5:5.5) gave 48 mg of an off-white solid (62% yield). ^1^H-NMR shows a 3:2 mixture of amide bond rotamers. ^1^H-NMR (300 MHz, DMSO-*d_6_*) δ 12.32–12.12 (m, 1H), 8.49–8.37 (m, 1H), 7.83–7.72 (m, 1H), 7.67–7.57 (m, 1H), 7.44–7.30 (m, 1H), 4.56–4.44 (m, 0.6H), 4.41–4.19 (m, 1.4H), 4.17–3.99 (m, 2H), 3.93–3.81 (m, 0.4H), 3.69–3.58 (m, 0.6H), 3.32–3.14 (m, 3.4H), 3.11–2.95 (m, 1.2H), 2.70–2.56 (m, 0.4H), 2.10–1.73 (m, 3H), 1.67–1.41 (m, 1H); ESI-MS: (*m*/*z*) 449.3 [M + Na]^+^, 425.3 [M − H]^−^; HPLC method A: t_r_ = 6.649 min.

##### (10) Detailed Procedures for the Preparation of Intermediates **43** and **44·HCl**

*tert*-Butyl (1-(2-cyanoacetyl)piperidin-3-yl)carbamate (**43**)

*tert*-Butyl piperidin-3-yl-carbamate (**42**) (1.0 g, 4.99 mmol) and cyanoacetic acid (470.0 mg, 5.49 mmol) were stirred in dry DCM (15 mL) at 0°C and under N_2_ atmosphere. A solution of DCC (1.1 g, 5.49 mmol) in dry DCM (11 mL) was drop-added. The mixture was stirred at rt overnight and then filtered rinsing the residue with fresh DCM. The filtrate was concentrated under reduced pressure. Purification of the residue by flash column chromatography (SiO_2_, DCM–EtOAc 7:3) gave 942 mg (71% yield); ^1^H-NMR shows a 3:2 mixture of amide bond rotamers. ^1^H-NMR (300 MHz, DMSO-*d6*) δ 7.08–6.49 (m, 1H), 4.10–3.88 (m, 2.6H), 3.81–3.67 (m, 0.4H), 3.53–3.19 (m, 2H, overlap with water signal), 3.06–2.91 (m, 1.4H), 2.69–2.58 (m, 0.6H), 1.85–1.36 (m, 13H).

1-(2-Cyanoacetyl)piperidin-3-amine hydrochloride (**44·HCl**)

4N HCl in dioxane (2.1 mL) was added to a solution of **43** (200.0 mg, 0.75 mmol) in dry THF (2 mL). The mixture was stirred at rt overnight. The resulting precipitate was filtered off, washed with Et_2_O, and dried under reduced pressure. The yield was 130 mg (96% crude yield), used in the next step without further purification. ^1^H-NMR shows a 3:2 mixture of amide bond rotamers. ^1^H-NMR (400 MHz, DMSO-*d_6_*) δ 8.61–8.25 (m, 3H), 4.26–3.95 (m, 2.4H), 3.70–3.64 (m, 0.6H), 3.55–3.50 (m, 0.4H), 3.45–3.36 (m, 1H), 3.31–3.04 (m, 2.6H), 2.03–1.89 (m, 1H), 1.77–1.62 (m, 2H), 1.54–1.36 (m, 1H); ^13^C NMR (101 MHz, DMSO-*d_6_*) δ 162.1, 161.7, 116.2, 116.1, 47.7, 46.1, 46.0, 45.3, 43.9, 41.6, 27.4, 27.1, 25.3, 25.2, 25.1, 22.0, 21.1.

##### (11) Detailed Procedures for the Preparation of Final Compounds **45**–**50**

3-(3-((9*H*-Pyrimido[4,5-*b*]indol-4-yl)amino)piperidin-1-yl)-3-oxopropanenitrile (**45**)

The title compound was prepared from **4a** (100.0 mg, 0.27 mmol), **44·HCl** (113.8 mg, 0.56 mmol), DIPEA (181.0 mg, 1.40 mmol), and Na*t*BuO (188.0 mg, 1.96 mmol) in dry DMF (5 mL) according to general procedure G. Purification by flash column chromatography (SiO_2_, DCM–EtOH gradient elution from 97:3 to 85:15) gave 34 mg (36% yield). ^1^H-NMR shows a 5:4 mixture of amide bond rotamers. ^1^H-NMR (300 MHz, DMSO-*d6*) δ 11.98–11.84 (m, 1H), 8.42–8.34 (m, 1H), 8.33–8.26 (m, 2H), 7.50–7.43 (m, 1H), 7.42–7.33 (m, 1H), 7.29–7.20 (m, 1H), 6.86–6.63 (m, 1H), 4.51–4.23 (m, 2H), 4.16–3.98 (m, 2H), 3.93–3.83 (m, 0.45H), 3.69–3.57 (m, 0.55H), 3.21–3.11 (m, 0.45H), 3.08–2.97 (m, 0.55H), 2.94–2.84 (m, 0.55H), 2.73–2.60 (m, 0.45H), 2.13–1.97 (m, 1H), 1.92–1.72 (m, 2H), 1.69–1.42 (m, 1H); ESI-MS: (*m*/*z*) 335.1 [M + H]^+^, 357.0 [M + Na]^+^, 332.9 [M − H]^−^; HPLC method B: t_r_ = 2.649 min.

3-(3-((7-Fluoro-9*H*-pyrimido[4,5-*b*]indol-4-yl)amino)piperidin-1-yl)-3-oxopropanenitrile (**46**)

The title compound was prepared from **4b** (100.0 mg, 0.27 mmol), **44·HCl** (81.3 mg, 0.40 mmol), DIPEA (171.9 mg, 1.33 mmol), and Na*t*BuO (179.0 mg, 1.86 mmol) in dry DMF (5 mL) according to general procedure G. The precipitate formed upon addition of saturated NH_4_Cl solution was not extracted with EtOAc but instead filtered off, washed with water, and dried over P_2_O_5_ in vacuo. Purification by flash column chromatography (SiO_2_, DCM–EtOH gradient elution from 94:6 to 9:1) gave 29 mg (31% yield). ^1^H-NMR shows a 5:4 mixture of amide bond rotamers. ^1^H-NMR (300 MHz, DMSO-*d6*) δ 12.14–11.96 (m, 1H), 8.41–8.27 (m, 2H), 7.26–7.18 (m, 1H), 7.17–7.06 (m, 1H), 6.92–6.73 (m, 1H), 4.49–4.23 (m, 2H), 4.16–3.97 (m, 2H), 3.93–3.83 (m, 0.45H), 3.70–3.59 (m, 0.55H), 3.20–3.08 (m, 0.45H), 3.08–2.95 (m, 0.55H), 2.90–2.80 (m, 0.55H), 2.72–2.61 (m, 0.45H), 2.13–1.95 (m, 1H), 1.88–1.74 (m, 2H), 1.68–1.42 (m, 1H); ^13^C NMR (101 MHz, DMSO-*d6*) δ 161.59, 161.56, 161.5, 161.4, 159.1, 159.1, 156.1, 155.6, 155.6, 154.4, 154.4, 137.1, 137.0, 122.9, 122.83, 122.80, 122.7, 116.2, 116.1, 116.02, 115.99, 107.9, 107.6, 97.9, 97.6, 95.7, 95.5, 50.0, 47.5, 46.4, 46.3, 45.8, 42.2, 29.83, 29.75, 24.94, 24.86, 24.3, 23.7; ESI-MS: (m/z) 375.3 [M + Na]^+^, 351.1 [M − H]^−^; HPLC method B: t_r_ = 3.525 min.

3-(3-((7-Chloro-9*H*-pyrimido[4,5-*b*]indol-4-yl)amino)piperidin-1-yl)-3-oxopropanenitrile (**47**)

The title compound was prepared by a two-step procedure.

In the first step **4c** (200.0 mg, 0.51 mmol), **44·HCl** (135.0 mg, 0.66 mmol), and DIPEA (197.7 mg, 1.53 mmol) were reacted in dry DMF (3.5 mL) at 70 °C for 19 h. Additional **44·HCl** (26.0 mg, 0.128 mmol) was added, and stirring at 70 °C continued for 6 h. After cooling down to rt, the mixture was poured into ice-cold water and saturated NH_4_Cl solution was added (30 mL). The resulting precipitate was filtered off, washed with water, and dried over P_2_O_5_ in vacuo. Purification by flash column chromatography (SiO_2_, DCM–MeOH 96.5:3.5) gave 104 mg of 3-(3-((7-Chloro-9-tosyl-9*H*-pyrimido[4,5-*b*]indol-4-yl)amino)piperidin-1-yl)-3-oxopropanenitrile as a pale yellow solid (39% yield). ^1^H-NMR shows a 5:4 mixture of amide bond rotamers. ^1^H-NMR (300 MHz, DMSO-*d6*) δ 8.55–8.44 (m, 1H), 8.42–8.30 (m, 2H), 8.00 (d, *J* = 8.4 Hz, 2H), 7.64–7.52 (m, 1H), 7.39 (d, *J* = 8.3 Hz, 2H), 7.22–7.05 (m, 1H), 4.44–4.19 (m, 2H), 4.15–3.92 (m, 2H), 3.84–3.74 (m, 0.45H), 3.67–3.57 (m, 0.55H), 3.16–3.06 (m, 0.45H), 3.05–2.92 (m, 0.55H), 2.87–2.75 (m, 0.55H), 2.70–2.55 (m, 0.45H), 2.32 (s, 3H), 2.07–1.90 (m, 1H), 1.90–1.68 (m, 2H), 1.68–1.35 (m, 1H); ESI-MS: (*m*/*z*) 544.8 [M + Na]^+^, 520.7 [M − H]^−^; HPLC method A: t_r_ = 8.439 min.

The purified material obtained from the first step (91.0 mg, 0.17 mmol) was reacted with K*t*BuO (136.7 mg, 1.22 mmol) in dry THF (10 mL) according to general procedure D (reaction time 2 h). Purification by flash column chromatography (SiO_2_, DCM–MeOH gradient elution from 95:5 to 92:8) gave 41 mg of a white solid (64% yield). ^1^H-NMR shows a 5:4 mixture of amide bond rotamers. ^1^H-NMR (300 MHz, DMSO-*d6*) δ 12.07 (s, 1H), 8.49–8.26 (m, 2H), 7.54–7.41 (m, 1H), 7.36–7.21 (m, 1H), 6.99–6.78 (m, 1H), 4.51–4.24 (m, 2H), 4.17–3.97 (m, 2H), 3.93–3.82 (m, 045H), 3.72–3.57 (m, 0.55H), 3.20–3.09 (m, 0.45H), 3.08–2.95 (m, 0.55H), 2.90–2.79 (m, 0.55H), 2.74–2.59 (m, 0.45H), 2.15–1.96 (m, 1H), 1.91–1.72 (m, 2H), 1.71–1.38 (m, 1H); ^13^C NMR (101 MHz, DMSO-*d6*) δ 161.6, 161.4, 155.90, 155.85, 155.8, 155.03, 154.99, 137.0, 129.1, 129.0, 122.9, 122.8, 120.0, 118.22, 118.18, 116.2, 116.1, 110.7, 95.6, 95.4, 49.9, 47.5, 46.5, 46.2, 45.8, 42.2, 29.8, 29.7, 24.94, 24.86, 24.3, 23.7; ESI-MS: (*m*/*z*) 391.0 [M + Na]^+^, 366.9 [M − H]^−^; HPLC method A: t_r_ = 6.023 min.

3-(3-((7-Bromo-9*H*-pyrimido[4,5-*b*]indol-4-yl)amino)piperidin-1-yl)-3-oxopropanenitrile (**48**)

**4d** (50.0 mg, 0.11 mmol), **44·HCl** (35.0 mg, 0.17 mmol), and DIPEA (73.7 mg, 0.57 mmol) were stirred in a solvent mixture of dry dioxane (1 mL) and dry DMF (0.1 mL) at 70 °C overnight. Additional **44·HCl** (35.0 mg, 0.17 mmol) and DIPEA (73.7 mg, 0.57 mmol) were added, and stirring at 70 °C continued overnight. The mixture was concentrated under reduced pressure, the residue diluted with dry THF (4 mL). Na*t*BuO (77.0 mg, 0.80 mmol) was added, and the mixture was stirred at rt for 1 h. Saturated NH_4_Cl solution (30 mL) was added, and the mixture was extracted with EtOAc (3 × 20 mL). Combined organic layers were dried over Na_2_SO_4_ and concentrated under reduced pressure. Purification of the residue by flash column chromatography (SiO_2_, DCM–EtOH gradient elution from 97:3 to 4:1) gave 25 mg (53% yield). ^1^H-NMR shows a 5:4 mixture of amide bond rotamers. ^1^H-NMR (400 MHz, DMSO-*d6*) δ 12.11–12.03 (m, 1H), 8.43–8.34 (m, 1H), 8.33–8.26 (m, 1H), 7.60 (s, 1H), 7.45–7.38 (m, 1H), 6.99–6.80 (m, 1H), 4.49–4.41 (m, 0.55H), 4.39–4.26 (m, 1.45H), 4.15–3.99 (m, 2H), 3.90–3.83 (m, 0.45H), 3.68–3.61 (m, 0.55H), 3.19–3.10 (m, 0.45H), 3.05–2.96 (m, 0.55H), 2.87–2.79 (m, 0.55H), 2.70–2.61 (m, 0.45H), 2.12–1.97 (m, 1H), 1.87–1.75 (m, 2H), 1.67–1.40 (m, 1H); ^13^C NMR (101 MHz, DMSO-*d6*) δ 161.5, 161.4, 155.9, 155.8, 155.7, 155.13, 155.09, 137.3, 123.2, 123.1, 122.7, 118.51, 118.47, 117.2, 117.1, 116.2, 116.1, 113.6, 95.6, 95.4, 49.9, 47.5, 46.5, 46.2, 45.8, 42.2, 29.8, 29.7, 24.9, 24.9, 24.3, 23.7; ESI-MS: (*m*/*z*) 434.8 [M + Na]^+^, 410.7 [M − H]^−^; HPLC method B: t_r_ = 5.144 min.

3-(3-((7-Methoxy-9*H*-pyrimido[4,5-*b*]indol-4-yl)amino)piperidin-1-yl)-3-oxopropanenitrile (**49**)

The title compound was prepared from **4f** (496.0 mg, 1.28 mmol), **44·HCl** (392.2 mg, 1.92 mmol), DIPEA (828.5 mg, 6.41 mmol), and Na*t*BuO (863.7 mg, 8.97 mmol) in dry DMF (20 mL) according to general procedure G but was stirred at rt for 3 d after addition of Na*t*BuO. Purification by flash column chromatography (SiO_2_, DCM–EtOH gradient elution from 97:3 to 4:1) gave 70 mg (15% yield). ^1^H-NMR shows a 5:4 mixture of amide bond rotamers. ^1^H-NMR (400 MHz, DMSO-*d6*) δ 11.88–11.77 (m, 1H), 8.37–8.27 (m, 1H), 8.22–8.16 (m, 1H), 6.98–6.94 (m, 1H), 6.90–6.84 (m, 1H), 6.74–6.57 (m, 1H), 4.48–4.24 (m, 2H), 4.15–4.00 (m, 2H), 3.91–3.81 (m, 3.45H), 3.67–3.59 (m, 0.55H), 3.18–3.09 (m, 0.45H), 3.06–2.96 (m, 0.55H), 2.89–2.82 (m, 0.55H), 2.71–2.61 (m, 0.45H), 2.11–1.98 (m, 1H), 1.88–1.74 (m, 2H), 1.66–1.40 (m, 1H); ^13^C NMR (101 MHz, DMSO-*d6*) δ 161.5, 161.4, 157.7, 157.6, 155.4, 155.2, 155.1, 153.5, 153.4, 137.7, 122.4, 122.2, 116.2, 116.1, 112.9, 112.8, 108.8, 96.1, 95.9, 95.0, 55.3, 50.1, 47.5, 46.3, 45.8, 42.2, 29.84, 29.76, 24.9, 24.8, 24.2, 23.7; ESI-MS: (*m*/*z*) 387.0 [M + Na]^+^, 363.1 [M − H]^−^; HPLC method B: t_r_ = 2.768 min.

3-Oxo-3-(3-((7-(trifluoromethyl)-9*H*-pyrimido[4,5-*b*]indol-4-yl)amino)piperidin-1-yl)propanenitrile (**50**)

The title compound was prepared from **4g** (180.0 mg, 0.42 mmol), **44·HCl** (129.1 mg, 0.63 mmol), DIPEA (272.7 mg, 2.11 mmol), and Na*t*BuO (284.4 mg, 2.96 mmol) in dry DMF (10 mL) according to general procedure G. Purification by flash column chromatography (SiO_2_, 1.DCM–EtOH gradient elution from 97:3 to 4:1, 2.DCM–(2N NH_3_ in MeOH) gradient elution from 99:1 to 92:8) gave 12 mg (7% yield). ^1^H-NMR shows a 5:4 mixture of amide bond rotamers. ^1^H-NMR (300 MHz, DMSO-*d6*) δ 12.31 (s, 1H), 8.60–8.51 (m, 1H), 8.50–8.39 (m, 1H), 7.73 (s, 1H), 7.62–7.53 (m, 1H), 7.17–6.98 (m, 1H), 4.52–4.24 (m, 2H), 4.16–3.97 (m, 2H), 3.93–3.83 (m, 1H), 3.71–3.61 (m, 1H), 3.22–3.12 (m, 1H), 3.08–2.96 (m, 1H), 2.92–2.82 (m, 1H), 2.73–2.62 (m, 1H), 2.15–1.97 (m, 1H), 1.92–1.74 (m, 2H), 1.70–1.40 (m, 1H); ESI-MS: (*m*/*z*) 424.9 [M + Na]^+^, 401.0 [M − H]^−^; HPLC method B: t_r_ = 5.880 min.

## Figures and Tables

**Table 2:** Structures and biological activities of compounds **2**, **18**–**24**, and **45**–**50**.

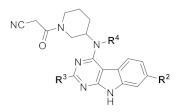				
Cpd.	R^2^	R^3^	R^4^	IC_50_ (µM) Mean ± SEM GSK-3β ^a^
**18**	–H	–H	–CH_3_	1.73 ± 0.03
**45**			–H	2.17 ± 0.02
**19**	–F	–H	–CH_3_	1.79 ± 0.18
**46**			–H	≥10
**2**	–Cl	–H	–CH_3_	1.86 ± 0.11 ^b^
**47**			–H	1.29 ± 0.27
**20**	–Br	–H	–CH_3_	1.42 ± 0.16
**48**			–H	1.94 ± 0.72
**21**	–I	–H	–CH_3_	0.94 ± 0.17
**22**	–OCH_3_	–H	–CH_3_	≥10
**49**			–H	≥10
**23**	–CF_3_	–H	–CH_3_	≥10
**50**			–H	≥10
**24**	–Cl	–CH_3_	–CH_3_	≥10

^a^ IC_50_ values were determined in an ADP Glo™ Kinase assay and are the means of at least two independent experiments; ^b^ data taken from [5].

**Table 3:** Structures and biological activities of compounds **16** and **25**–**29**.

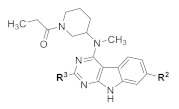			
Cpd.	R^2^	R^3^	IC_50_ (µM) Mean ± SEM GSK-3β ^a^
**25**	–H	–H	4.58 ± 0.74
**26**	–F	–H	4.81 ± 0.61
**16**	–Cl	–H	1.71 ± 0.52
**27**	–Cl	–CH_3_	n.d. ^b^
**28**	–Br	–H	0.71 ± 0.02
**29**	–I	–H	3.75 ± 0.65

^a^ IC50 values were determined in an ADP Glo™ Kinase assay and are the means of at least two independent experiments; ^b^ not determined.

**Table 4:** Structure and biological activities of compounds **16** and **30**–**37** as well as **2**, **20**, and **28** and their enantiopure analogs.

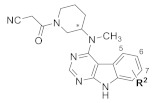			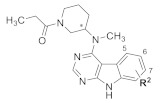		
Cpd.	R^2^	IC_50_ (µM) Mean ± SEM GSK-3β ^a^	Cpd.	R^2^	IC_50_ (µM) Mean ± SEM GSK-3β ^a^
**2**	7-Cl	1.86 ± 0.11 ^b^	**16**	7-Cl	1.71 ± 0.52
***(R)*-2**		0.48 ± 0.04			
***(S)*-2**		≥10			
**20**	7-Br	1.42 ± 0.16	**28**	7-Br	0.71 ± 0.02
***(R)*-20**		0.84 ± 0.07	***(R)*-28**		0.36 ± 0.05
***(S)*-20**		≥10	***(S)*-28**		n.d. ^c^
**30**	6-Cl	2.30 ± 0.07	**34**	6-Cl	1.25 ± 0.03
**31**	6-Br	2.01 ± 0.20	**35**	6-Br	1.27 ± 0.47
**32**	6-OCH_3_	2.54 ± 0.11	**36**	6-OCH_3_	1.18 ± 0.03
**33**	5-Cl	8.20 ± 1.27	**37**	5-Cl	≥10

^a^ IC50 values were determined in an ADP Glo™ Kinase assay and are the means of at least two independent experiments; ^b^ data taken from [5]; ^c^ not determined.

## Supplementary Materials

The following are available online at https://www.mdpi.com/1422-0067/21/21/7823/s1: the ADP Glo™ assay protocol; additional data for inhibitors ***(R)*-2** and ***(R)*-28** including interaction frequencies of 1 µs molecular dynamics simulations, microsomal stability assay, cell toxicity data on five different cell lines, cellular GSK-3α/β inhibition, neuroprotective effects, and MD movies as well as preparation of 4-chloro-9*H*-pyrimido[4,5-*b*]indoles **3a–l**. Figure S1: Observed interactions of ***(R)*-2** and ***(R)*-28** in the MD simulations; Figure S2: Evaluation of the cytotoxic potential of ***(R)*-28** cell lines; Figure S3: Evaluation of the cytotoxic potential of ***(R)*-28** on cancer cell lines; Figure S4: Compound ***(R)*-28** inhibits GSK-3β activity in neuronal SH-SY5Y cells; Figure S5: Neuroprotective effects of ***(R)*-28** against the neurotoxicity induced by H_2_O_2_ and OAβ_1–42_ in neuronal SH-SY5Y cells; Table S1: Chromatographic gradient for separation of metabolism analytes. Scheme S1: Synthetic route to 4-chloro-9*H*-pyrimido[4,5-*b*]indoles **3a**–**l**. The QM output conformations and full raw trajectories are freely available at https://doi.org/10.5281/zenodo.3973296.

## References

[1] Kaidanovich-Beilin O., Woodgett J. (2011). GSK-3: Functional Insights from Cell Biology and Animal Models. Front. Mol. Neurosci..

[2] Sutherland C. (2011). What are the bona fide GSK3 Substrates?. Int. J. Alzheimer’s Dis..

[3] Beurel E., Grieco S.F., Jope R.S. (2015). Glycogen synthase kinase-3 (GSK3): Regulation, actions, and diseases. Pharmacol. Ther..

[4] Lauretti E., Dincer O., Praticò D. (2020). Glycogen synthase kinase-3 signaling in Alzheimer’s disease. Biochim. Biophys. Acta.

[5] Andreev S., Pantsar T., Ansideri F., Kudolo M., Forster M., Schollmeyer D., Laufer S.A., Koch P. (2019). Design, Synthesis and Biological Evaluation of 7-Chloro-9H-pyrimido[4,5-b]indole-based Glycogen Synthase Kinase-3β Inhibitors. Molecules.

[6] Gorrod J.W., Aislaitner G. (1994). The metabolism of alicyclic amines to reactive iminium ion intermediates. Eur. J. Drug Metab. Pharmacokinet..

[7] Meanwell N.A. (2018). Fluorine and Fluorinated Motifs in the Design and Application of Bioisosteres for Drug Design. J. Med. Chem..

[8] Bull J.A., Croft R.A., Davis O.A., Doran R., Morgan K.F. (2016). Oxetanes: Recent Advances in Synthesis, Reactivity, and Medicinal Chemistry. Chem. Rev..

[9] Wuitschik G., Carreira E.M., Wagner B., Fischer H., Parrilla I., Schuler F., Rogers-Evans M., Müller K. (2010). Oxetanes in Drug Discovery: Structural and Synthetic Insights. J. Med. Chem..

[10] Heider F., Ansideri F., Tesch R., Pantsar T., Haun U., Döring E., Kudolo M., Poso A., Albrecht W., Laufer S.A. (2019). Pyridinylimidazoles as dual glycogen synthase kinase 3β/p38α mitogen-activated protein kinase inhibitors. Eur. J. Med. Chem..

[11] Zegzouti H., Zdanovskaia M., Hsiao K., Goueli S.A. (2009). ADP-Glo: A Bioluminescent and Homogeneous ADP Monitoring Assay for Kinases. Assay Drug Dev. Technol..

[12] Pantsar T., Singha P., Nevalainen T.J., Koshevoy I., Leppänen J., Poso A., Niskanen J.M.A., Pasonen-Seppänen S., Savinainen J.R., Laitinen T. (2017). Design, synthesis, and biological evaluation of 2,4-dihydropyrano[2,3-c]pyrazole derivatives as autotaxin inhibitors. Eur. J. Pharm. Sci..

[13] Laufer S.A., Hauser D.R.J., Domeyer D.M., Kinkel K., Liedtke A.J. (2008). Design, Synthesis, and Biological Evaluation of Novel Tri- and Tetrasubstituted Imidazoles as Highly Potent and Specific ATP-Mimetic Inhibitors of p38 MAP Kinase: Focus on Optimized Interactions with the Enzyme’s Surface-Exposed Front Region. J. Med. Chem..

[14] Di Martino R.M.C., Pruccoli L., Bisi A., Gobbi S., Rampa A., Martinez A., Pérez C., Martinez-Gonzalez L., Paglione M., Di Schiavi E. (2020). Novel Curcumin-Diethyl Fumarate Hybrid as a Dualistic GSK-3β Inhibitor/Nrf2 Inducer for the Treatment of Parkinson’s Disease. ACS Chem. Neurosci..

[15] Tarozzi A., Morroni F., Merlicco A., Hrelia S., Angeloni C., Cantelli-Forti G., Hrelia P. (2009). Sulforaphane as an inducer of glutathione prevents oxidative stress-induced cell death in a dopaminergic-like neuroblastoma cell line. J. Neurochem..

[16] Tarozzi A., Bartolini M., Piazzi L., Valgimigli L., Amorati R., Bolondi C., Djemil A., Mancini F., Andrisano V., Rampa A. (2014). From the dual function lead AP2238 to AP2469, a multi-target-directed ligand for the treatment of Alzheimer’s disease. Pharmacol. Res. Perspect..

[17] Pruccoli L., Morroni F., Sita G., Hrelia P., Tarozzi A. (2020). Esculetin as a Bifunctional Antioxidant Prevents and Counteracts the Oxidative Stress and Neuronal Death Induced by Amyloid Protein in SH-SY5Y Cells. Antioxidants.

[18] Showalter H.D.H., Bridges A.J., Zhou H., Sercel A.D., McMichael A., Fry D.W. (1999). Tyrosine Kinase Inhibitors. 16. 6,5,6-Tricyclic Benzothieno[3,2-d]pyrimidines and Pyrimido[5,4-b]- and -[4,5-b]indoles as Potent Inhibitors of the Epidermal Growth Factor Receptor Tyrosine Kinase. J. Med. Chem..

[19] Tichý M., Pohl R., Xu H.Y., Chen Y.-L., Yokokawa F., Shi P.-Y., Hocek M. (2012). Synthesis and antiviral activity of 4,6-disubstituted pyrimido[4,5-b]indole ribonucleosides. Bioorganic Med. Chem..

[20] Tichý M., Pohl R., Tloušt’ová E., Weber J., Bahador G., Lee Y.-J., Hocek M. (2013). Synthesis and biological activity of benzo-fused 7-deazaadenosine analogues. 5- and 6-substituted 4-amino- or 4-alkylpyrimido[4,5-b]indole ribonucleosides. Bioorganic Med. Chem..

[21] Gehringer M., Forster M., Pfaffenrot E., Bauer S.M., Laufer S.A. (2014). Novel Hinge-Binding Motifs for Janus Kinase 3 Inhibitors: A Comprehensive Structure–Activity Relationship Study on Tofacitinib Bioisosteres. ChemMedChem.

[22] Manley D.W., McBurney R.T., Miller P., Walton J.C., Mills A., O’Rourke C. (2014). Titania-Promoted Carboxylic Acid Alkylations of Alkenes and Cascade Addition–Cyclizations. J. Org. Chem..

[23] Gehringer M., Pfaffenrot E., Bauer S., Laufer S.A. (2014). Design and Synthesis of Tricyclic JAK3 Inhibitors with Picomolar Affinities as Novel Molecular Probes. ChemMedChem.

[24] Roos K., Wu C., Damm W., Reboul M., Stevenson J.M., Lu C., Dahlgren M.K., Mondal S., Chen W., Wang L. (2019). OPLS3e: Extending Force Field Coverage for Drug-Like Small Molecules. J. Chem. Theory Comput..

[25] Bochevarov A.D., Harder E., Hughes T.F., Greenwood J.R., Braden D.A., Philipp D.M., Rinaldo D., Halls M.D., Zhang J., Friesner R.A. (2013). Jaguar: A high-performance quantum chemistry software program with strengths in life and materials sciences. Int. J. Quantum Chem..

[26] Heider F., Pantsar T., Kudolo M., Ansideri F., De Simone A., Pruccoli L., Schneider T., Goettert M.I., Tarozzi A., Andrisano V. (2019). Pyridinylimidazoles as GSK3β Inhibitors: The Impact of Tautomerism on Compound Activity via Water Networks. ACS Med. Chem. Lett..

[27] Madhavi Sastry G., Adzhigirey M., Day T., Annabhimoju R., Sherman W. (2013). Protein and ligand preparation: Parameters, protocols, and influence on virtual screening enrichments. J. Comput. Aided Mol. Des..

[28] Sivaprakasam P., Han X., Civiello R.L., Jacutin-Porte S., Kish K., Pokross M., Lewis H.A., Ahmed N., Szapiel N., Newitt J.A. (2015). Discovery of new acylaminopyridines as GSK-3 inhibitors by a structure guided in-depth exploration of chemical space around a pyrrolopyridinone core. Bioorg. Med. Chem. Lett..

[29] Bowers K.J., Chow E., Xu H., Dror R.O., Eastwood M.P., Gregersen B.A., Klepeis J.L., Kolossvary I., Moraes M.A., Sacerdoti F.D. (2006). Scalable Algorithms for Molecular Dynamics Simulations on Commodity Clusters, Proceedings of the 2006 ACM/IEEE Conference on Supercomputing, Tampa, FL, USA, 11–17 November 2006.

[30] Jorgensen W.L., Chandrasekhar J., Madura J.D., Impey R.W., Klein M.L. (1983). Comparison of simple potential functions for simulating liquid water. J. Chem. Phys..

