# Supplementary material for: Discovery and Evaluation of Enantiopure 9H-pyrimido[4,5-b]indoles as Nanomolar GSK-3β Inhibitors with Improved Metabolic Stability
Source: Int J Mol Sci. 2020 Oct 22;21(21):7823. doi: 10.3390/ijms21217823 (PMC7659979; doi:10.3390/ijms21217823)
Supplement: Supplementary file 1 [file ijms-21-07823-s001.zip › ijms-945464- Supplementary Materials corrected.pdf]

International Journal of *Molecular Sciences*

MDPI

Supplementary Materials

Discovery and Evaluation of Enantiopure 9*H*-pyrimido[4,5-*b*]indoles as Nanomolar GSK-3β Inhibitors with Improved Metabolic Stability

Stanislav Andreev [1], Tatu Pantsar [1,2], Ahmed El-Gokha [1,3], Francesco Ansideri [1], Mark Kudolo [1], Débora Bublitz Anton [4], Giulia Sita [5], Jenny Romasco [6], Christian Geibel [7], Michael Lämmerhofer [7], Márcia Ines Goettert [4], Andrea Tarozzi [6], Stefan A. Laufer [1] and Pierre Koch [1,8,*]

[1] Institute of Pharmaceutical Sciences, Department of Medicinal and Pharmaceutical Chemistry, Eberhard Karls University Tübingen, Auf der Morgenstelle 8, 72076 Tübingen, Germany; stanislav.andreev@uni-tuebingen.de (S.A.); tatu.pantsar@uni-tuebingen.de (T.P.); ahmed.abdelaleem@science.menofia.edu.eg (A.E.-G.); francesco.ansideri@uni-tuebingen.de (F.A.); mark.kudolo@uni-tuebingen.de (M.K.); stefan.laufer@uni-tuebingen.de (S.L.)

[2] School of Pharmacy, Faculty of Health Sciences, University of Eastern Finland, P.O. Box 1627, 70211 Kuopio, Finland

[3] Chemistry Department, Faculty of Science, Menoufia University, 32511 Shebin El-Kom, Egypt

[4] Cell Culture Laboratory, Postgraduate Program in Biotechnology, University of Vale do Taquari (Univates), 95914-014 Lajeado, Brazil; debora.anton@univates.br (D.B.A.); marcia.goettert@univates.br (M.I.)

[5] Department of Pharmacy and Biotechnology, Alma Mater Studiorum, University of Bologna, Via Irnerio, 48, 40126 Bologna, Italy; giulia.sita2@unibo.it

[6] Department for Life Quality Studies, Alma Mater Studiorum, University of Bologna, Corso D'Augusto, 237, 47921 Rimini, Italy; jenny.romasco@unibo.it (J.R.); andrea.tarozzi@unibo.it (A.T.)

[7] Institute of Pharmaceutical Sciences, Department of Pharmaceutical (Bio-)Analysis, Eberhard Karls University Tübingen, Auf der Morgenstelle 8, 72076 Tübingen, Germany; christian.geibel@uni-tuebingen.de (C.G.); michael.laemmerhofer@uni-tuebingen.de (M.L.)

[8] Department of Pharmaceutical/Medicinal Chemistry II, Institute of Pharmacy, University of Regensburg, Universitätsstraße 31, 93053 Regensburg, Germany

* Correspondence: pierre.koch@uni-tuebingen.de; Tel.: +49-(941)-943-2847

Received: 10 September 2020; Accepted: 16 October 2020; Published: 22 October 2020

Table of Contents

1. ADP Glo™ Kinase assay 3

2. Interaction Frequencies of ***(R)*-2** and ***(R)*-28** in the 1 μs Molecular Dynamics Simulations 3

3. Microsomal Stability Assay 3

4. Investigation of Cell Toxicity of ***(R)*-28** on Five Different Cell Lines 4

5. Investigation of Cellular GSK-3α/β Inhibition by ***(R)*-28** and Its Neuroprotective Effects 7

6. Preparation of 4-chloro-9*H*-pyrimido[4,5-*b*]indoles **3a–l** 9

1. ADP Glo™ Kinase Assay

The inhibitory activity of the final compounds on GSK-3β was determined by the Promega ADP-Glo™ Kinase assay (Promega Corporation, Madison, WI 53711, USA), which was performed in white, non-treated 384-well plates (Corning). The experiments were carried out as quadruplicates using a concentration of 0.58 ng/µL of recombinant human GSK-3β, 0.2 µg/µL GSK-3 substrate G50-58 (sequence: YRRAAVPPSPSLSRHSSPHQ(pS)EDEEE) and 25 µM ATP in the presence of serial dilutions of the final compounds. In addition, two control experiments with uninhibited kinase and two blank experiments with ATP/substrate solution were performed.

In detail, the kinase was pre-incubated with the final compounds for 10 min at rt. Then substrate/ATP was added to start the reaction, which was run for 1 h at rt. The next steps consisted of addition of ADP-Glo™ reagent (5 µL, then 1 h incubation) and Kinase detection reagent (10 µL, then 30 min incubation). Finally, the luminescence was measured on a FilterMax F5 microplate reader (Molecular Devices) (integration time 500 ms). GraphPad Prism v. 7.03. was used to normalize the raw data to the values of the control and blank experiments and generate absolute IC_{50} values.

2. Interaction Frequencies of *(R)*-2 and *(R)*-28 in the 1 µs Molecular Dynamics Simulations

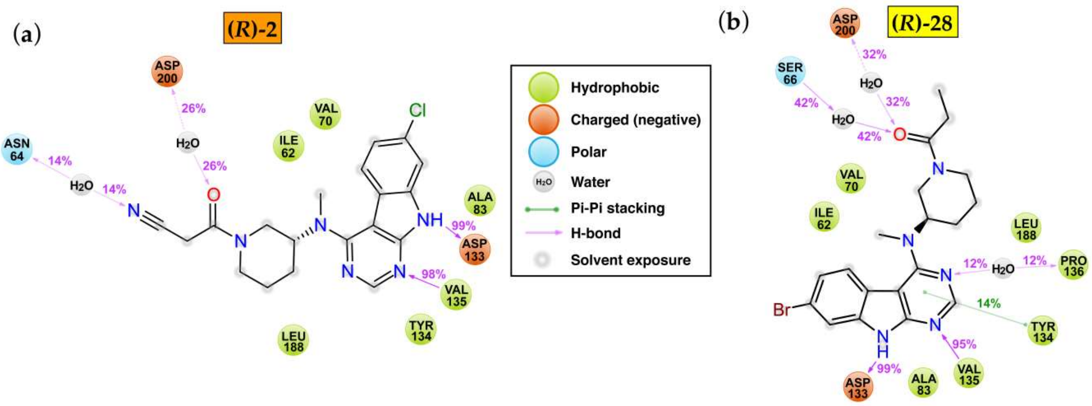

Figure S1. Observed interactions of ***(R)*-2 (a)** and ***(R)*-28 (b)** in the MD simulations. Both ligands demonstrate stable hinge binding (95–99% Asp133 and Val135 interactions). The carbonyl group mediates water-bridged interactions to Asp200 or Ser66. Interactions with >10% frequency are shown.

3. Microsomal Stability Assay

Pooled male and female human liver microsomes (HLMs) (Lot: SLBQ7487V) were purchased from Merck (Schnelldorf, Germany). The compounds (***(R)*-2** and ***(R)*-28**, respectively) (100 µM), 4 mM $MgCl_2 \cdot 6\ H_2O$ in 0.1 M Tris buffer (pH 7.4) and an NADPH-regenerating system (5 mM glucose-6-phosphate, 5 U/mL glucose-6-phosphate dehydrogenase and 1 mM $NADP^+$) were preincubated for 5 min at 37 °C and 750× rpm on a shaker. The HLMs were added to start the reaction. The reaction mixture was then split into triplicates of 50 µL. The reaction was quenched at six time points (0, 10, 20, 30, 60 and 120 min) by addition of 100 µL internal standard (30 µM in MeCN). The samples were vortexed for 30 s and centrifuged (19,800 relative centrifugal force/4 °C/10 min). The obtained supernatant was directly used for LC-MS analysis. A limit of 1% organic solvent was not exceeded.
Positive control: Propranolol
Negative control: Heat inactivated microsomes

The metabolite formation was analyzed with an Alliance 2695 Separations Module (Waters GmbH, Eschborn).
Sample temperature: 4 °C
Column: Phenomenex Kinetex C18 column (100 × 3 mm; 2.6 µm; 100 Å)
Column temperature: 40 °C

Injection volume: 5 µL
Flow rate: 0.6 mL/min
Gradient: see Table below

Table S1. Chromatographic gradient for separation of metabolism analytes.

Time (min)	Solvent A (%) (90% H_2O, 10% MeCN, 0.1% formic acid)	Solvent B (%) (MeCN, 0.1% formic acid)
0	90	10
2.5	90	10
10	45	55
12	45	55
12.01	90	10
15	90	10

The detection was performed on a Micromass Quattro micro triple quadrupole mass spectrometer (Waters GmbH, Eschborn).

Ionization mode: Electrospray ionization, positive mode
Spray voltage: 4.5 kV
Desolvation temperature: 250 °C
Desolvation gas flow: 600 L/h

4. Investigation of Cell Toxicity on Five Different Cell Lines

4.1. Maintenance of Cell Culture

The experiments were performed in an *in vitro* model of cell cytoxicity analyses on hepatocellular carcinoma (HepG2), human breast adenocarcinoma (MCF-7), human neuroblastoma (SH-SY5Y), human lung fibroblast (MRC-5) and chinese hamster ovary (CHO-K1). The cells lines were cultured in appropriate medium, supplemented with 10% (*v*/*v*) of fetal bovine serum (FBS) and 1% antibiotic/antimycotic solution. For subculture, cells were dissociated with trypsin-EDTA (Cultilab), split into a 1:3 ratio and subcultured into Petri dishes with 25 cm^2 growth area. Culture medium was replaced every 2 days until the cells reached the total confluence after 4–5 days of initial seeding. Cells were maintained in the following controlled conditions: 95% of humidified atmosphere, 5% of CO_2 and constant temperature of 37 °C.

4.2. Cytotoxicity

The assessment of cell viability was performed according to the MTT colorimetric assay. The cytotoxicity of the compounds was assessed on 5 different cell lines. Cells (5×10^3/well) in 200 µL appropriate medium containing 10% FBS were seeded on 96-well plates and incubated overnight. These cells were subsequently treated with different concentrations of compound ***(R)*-28** for 48 h. The effects were estimated by colorimetric assay based on the conversion of tetrazolium salts (MTT) after 3 h of incubation to a blue formazan product by active mitochondria. The absorbance was read at 570 nm using a Spectramax i5 microplate reader. Results were expressed as percentage of control.

Cell Lines

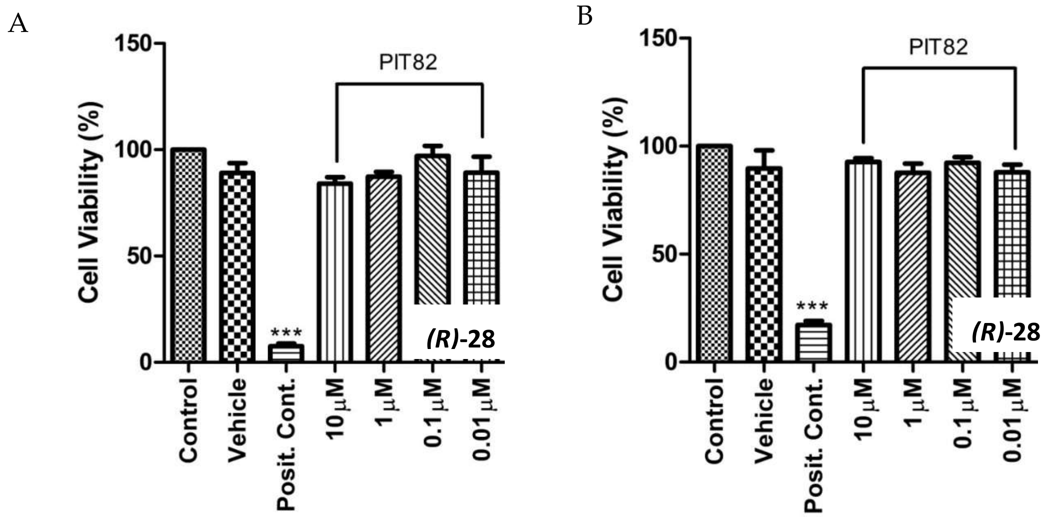

Figure S2. Evaluation of the cytotoxic potential of ***(R)*-28** on cell lines. (**A**) Human lung fibroblast cell line MRC-5 and (**B**) chinese hamster ovary cell line CHO-K1. Cell viability was evaluated by MTT assay, after 48 h treatment with ***(R)*-28**. Mean values ± SEM of three independent experiments are shown. *** $p < 0.001$ compared with control. ***(R)*-28**; Vehicle (DMSO); Positive Control (hydrogen peroxide).

Cancer Cell Lines

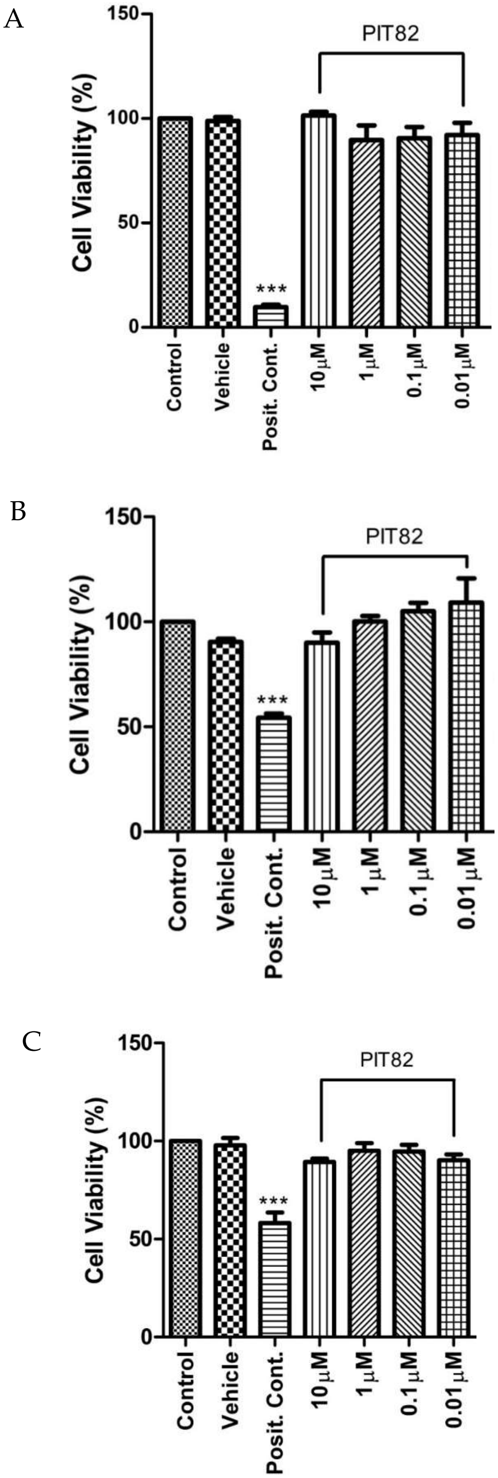

Figure S3. Evaluation of the cytotoxic potential of ***(R)*-28** on cancer cell lines. (**A**) Hepatocellular carcinoma cell line HepG2, (**B**) human breast adenocarcinoma cell line MCF-7 and (**C**) human neuroblastoma cell line SH-SY5Y. Cell viability was evaluated by MTT assay, after 48 h treatment with ***(R)*-28**. Mean values ± SEM of three independent experiments are shown. *** $p < 0.001$ compared with control. ***(R)*-28**; Vehicle (DMSO); Positive Control (hydrogen peroxide).

5. Investigation of Cellular GSK-3α/β Inhibition by *(R)*-28 and Its Neuroprotective Effects

5.1. Cell Cultures

Human neuronal SH-SY5Y cells (Sigma Aldrich, St. Louis, MO, USA) were routinely grown in Dulbecco's modified Eagle's Medium supplemented with 10% fetal bovine serum, 2 mM L-glutamine, 50 U/mL penicillin and 50 μg/mL streptomycin at 37 °C in a humidified incubator with 5% CO_2.

5.2. Neuronal Viability

SH-SY5Y cells were seeded in a 96-well plate at 2×10^4 cells/well, incubated for 24 h and then treated with various concentrations (1.25–40 μM) of ***(R)*-28** for 24 h. Cell viability, in terms of mitochondrial activity, was evaluated by MTT assay, as previously described [14].

5.3. Neuroprotective Activity toward H_2O_2

SH-SY5Y cells were seeded in a 96-well plate at 2×10^4 cells/well, incubated for 24 h and subsequently treated with ***(R)*-28** (5 μM) and H_2O_2 (100 μM) for 1 h. Then, cells were starved in complete medium for 22 h. The neuroprotective activity was measured by using the MTT assay as previously described [15]. Data are expressed as a percentage of neurotoxicity versus untreated cells.

5.4. $OA\beta_{1\text{–}42}$ Preparation for the Determination of Neuroprotective Activity

$A\beta_{1\text{–}42}$ peptide (AnaSpec, Fremont, CA, USA) was first dissolved in 1,1,1,3,3,3-hexafluoroisopropanol to 1 mg/mL, sonicated, incubated at rt for 24 h, and lyophilised to obtain an unaggregated $A\beta_{1\text{–}42}$ peptide film that was solubilised with DMSO and stored at −20 °C until use. The aggregation of $A\beta_{1\text{–}42}$ peptide into oligomers was performed as previously described [16].

5.5. Neuroprotective Activity toward $OA\beta_{1\text{–}42}$

SH-SY5Y cells were seeded in a 96-well plate at 3×10^4 cells/well, incubated for 24 h, and subsequently treated with ***(R)*-28** (5 μM) and $OA\beta_{1\text{-}42}$ (10 μM) for 4 h. The neuroprotective activity was measured by using the MTT assay as previously described [17]. Data are expressed as a percentage of neurotoxicity versus untreated cells.

5.6. Western Blotting

SH-SY5Y cells were seeded in 60 mm dishes at 2×10^6 cells/dish, incubated for 24 h and subsequently treated with ***(R)*-28** (1 μM) for 1 h at 37 °C in 5% CO_2. At the end of incubation, cells were trypsinized and the cellular pellet was resuspended in complete lysis buffer containing leupeptin (2 μg/mL), PMSF (100 μg/mL) and cocktail of protease/phosphatase inhibitors (100×). Small amounts were removed for the determination of the protein concentration using the Bradford method. The samples (30 μg proteins) were run on 4–15% SDS polyacrylamide gels (Bio-rad Laboratories S.r.L., Hercules, CA, USA) and electroblotted onto 0.45 μm nitrocellulose membranes. The membranes were incubated at 4 °C overnight with primary antibody recognizing phospho-GSK3α/β (Ser21/9), (1:1000; Cell Signaling Technology Inc, Danvers, MA, USA), or anti-phospho-GSK3(Tyr279/Tyr216), (1:1000; EMD Millipore, Darmstadt, Germany). After washing with TBS-T (TBS +0.05% Tween20), the membranes were incubated with secondary antibodies (1:2000; GE Healthcare). Enhanced chemiluminescence was used to visualize the bands (ECL; Bio-rad Laboratories). The membranes were then reprobed with GSK3α/β, (1:1000; Cell Signaling Technology Inc.). The data were analyzed by densitometry, using Quantity One software (Bio-Rad Laboratories® S.r.L.). The values were normalized and expressed as mean ± SD of densitometry in each experimental group.

5.7. Statistical Analysis

Results are shown as mean ± standard deviation (SD) of three independent experiments. Statistical analysis was performed using Student's t-test. Differences were considered significant at $p < 0.05$. Analyses were performed using GraphPad PRISM software (v. 5.0; GraphPad Software, La Jolla, CA, USA) on a Windows platform.

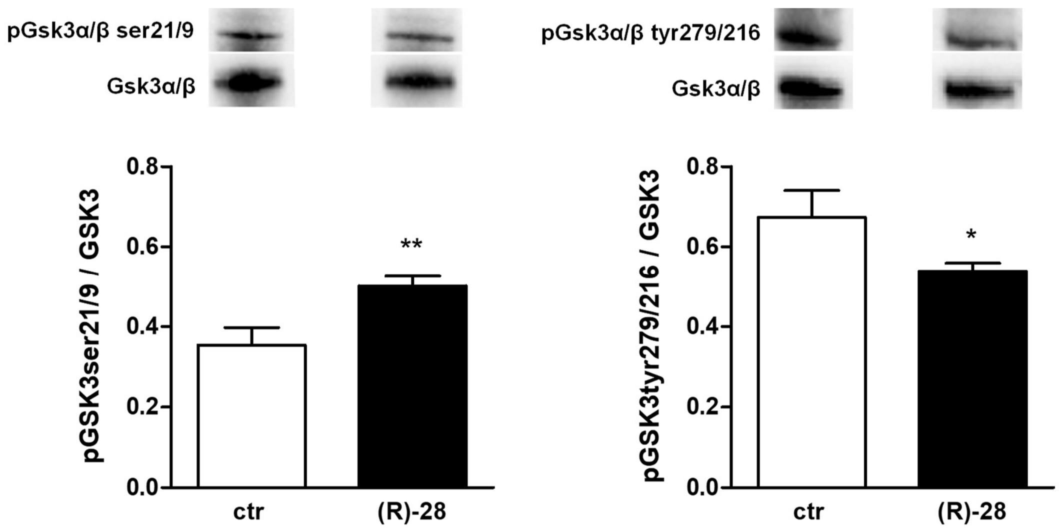

Figure S4. Compound ***(R)*-28** inhibits the GSK-3β activity in neuronal SH-SY5Y cells. Cells were incubated with ***(R)*-28** (1 μM) for 1 h. At the end of incubation, the phosphorylation of GSK-3α/β (Ser21/9) (inactive GSK-3α/β form) and GSK-3α/β (Tyr279/Tyr216) (active GSK-3α/β form) was determined by western blotting. Data are expressed as ratio between phospho-GSK-3α/β and total GSK-3β levels normalized against β-Actin. Mean values ± SD of at least three independent experiments are shown (* $p < 0.05$ and ** $p < 0.01$ vs untreated cells).

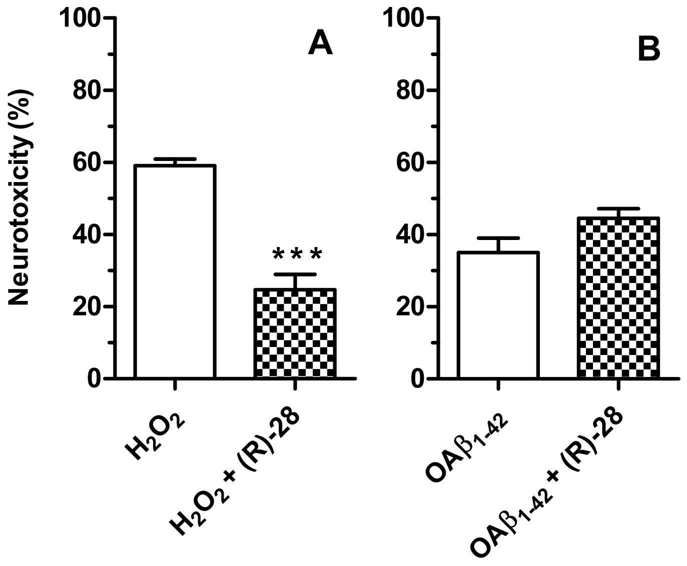

Figure S5. Neuroprotective effects of ***(R)*-28** against the neurotoxicity induced by H_2O_2 and $OA\beta_{1\text{-}42}$ in SH-SY5Y cells. **(A)** Cells were incubated with ***(R)*-28** (5 µM) and H_2O_2 (100 µM) for 1 h and then starved in complete medium for 22 h. **(B)** Cells were incubated with ***(R)*-28** (5 µM) and $OA\beta_{1\text{-}42}$ (10 µM) for 4 h. The neurotoxicity was evaluated by MTT assay as described above. Data are expressed as percentages of neurotoxicity versus untreated cells. Mean values ± SD of three independent experiments are shown (*** $p < 0.001$ vs cells treated with H_2O_2).

6. Preparation of 4-chloro-9*H*-pyrimido[4,5-*b*]indoles 3a–l

The substitution pattern of the non-aromatic ring in the 9*H*-pyrimido[4,5-*b*]indole core was defined by utilizing appropriate *o*-halonitrobenzenes (**S1a**–**k**) in the first step of the synthetic route (Scheme S1). These commercially available starting materials were reacted with ethyl cyanoacetate under highly basic conditions giving substitution products **S2a**–**k**. Reductive ring closure was then effected with elemental zinc in acetic acid and typically generated 2-aminoindoles **S3a**–**k** along with characteristic by-products, which presumably are the corresponding 1-hydroxy-2-aminoindoles [18]. However, a purification of these mixtures was unnecessary in most cases, as the following condensation with formamide predominently afforded the desired 3,9-dihydro-4*H*-pyrimido[4,5-*b*]indol-4-ones **S1a**–**k**. The introduction of a methyl group in the 2-position of the pyrimidine ring of **S4l** was achieved applying a protocol modified from Showalter et al. [18]. To this end, **S3c** was converted to an amidine intermediate with acetonitrile, which was then cyclized to **S4l** with aqueous NaOH in EtOH. The reaction of **S4a**–**l** with $POCl_3$ finally delivered 4-chloro-9*H*-pyrimido[4,5-*b*]indoles **3a**–**l**.

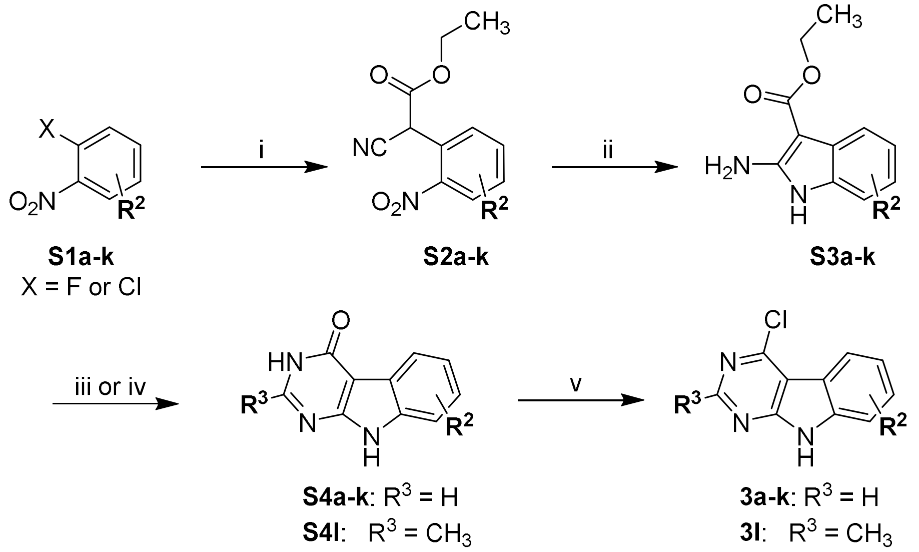

Scheme S1. Synthetic route to 4-chloro-9*H*-pyrimido[4,5-*b*]indoles **3a–l**. For the definition of R^2 see Scheme 1 in the main manuscript. Reagents and conditions: (i) ethyl cyanoacetate, NaH, DMF, 0 °C to rt or 75 °C in case of **S2a–g** or ethyl cyanoacetate, K*t*BuO, THF, 0 °C to 60 °C in case of **S2h–k** (ii) Zn^0, AcOH, 90 °C; (iii) NH_4HCOO, formamide, 160 °C in case of **S4a–d** and **S4h–k** or formamide, 160–190 °C in case of **S4e–g**; (iv) (1) **S3c**, HCl, MeCN, rt to reflux, (2) $NaOH_{(aq)}$, EtOH, reflux (in case of **S4l**); (v) $POCl_3$, DIPEA, chlorobenzene, rt to 80–100 °C in case of **3a, 3c–e**, **3h,i** and **3k,l** or DIPEA, $POCl_3$ (neat), rt to 80 °C in case of **3b**, **3f** and **3j** or $POCl_3$ (neat), rt to 80 °C in case of **3g**.

6.1. General Procedure H

The appropriate intermediate was dissolved in glacial AcOH. The solution was heated to 80–90 °C and zinc powder was added in portions. The suspension was stirred at 80–90 °C until reaction control by HPLC indicated complete consumption of the starting material. The suspension was left to cool down to rt and filtered rinsing the residue with glacial AcOH or EtOAc. The filtrate was concentrated under reduced pressure to leave a residue of AcOH which was neutralized by addition of saturated $NaHCO_3$ solution. The resulting precipitate was filtered off, washed with demineralised water and dried over P_2O_5 in vacuo. The crude material isolated from these reactions typically consisted of a mixture of the title compounds and the corresponding 1-hydroxyindoles. These crude mixtures were used in the next step without further purification.

6.2. General Procedure I

The appropriate intermediate was suspended in formamide and ammonium formate was added. The mixture was stirred at 160 °C until reaction control by HPLC indicated complete consumption of the starting material. The suspension was left to cool down to rt and poured into ice-cold water. The resulting precipitate was filtered off, rinsed thoroughly with demineralised water and dried over P_2O_5 in vacuo. The crude product was used in the next step without further purification.

6.3. General Procedure J

The appropriate intermediate was suspended in chlorobenzene. DIPEA was added followed by careful addition of $POCl_3$ (used in stochiometric amounts or as co-solvent). The mixture was stirred at 80–100 °C until reaction control by HPLC indicated complete consumption of the starting material. After cooling down to rt the mixture was carefully transferred into stirring demineralised water of rt. The highly acidic aqueous mixture was neutralized with aqueous NaOH solution. The resulting precipitate was filtered off, rinsed thoroughly with demineralised water and dried over P_2O_5 in

vacuo. If not stated otherwise, the crude material was purified by hot filtration from toluene as described previously [5].

Detailed Procedures for the Preparation of Intermediates S3a–k.

Ethyl 2-amino-1*H*-indole-3-carboxylate (**S3a**)

The title compound was prepared by a two-step procedure. In the first step ethyl cyanoacetate (32.1 g, 283.79 mmol) was drop-added to an ice-cooled stirring suspension of NaH (11.4 g of a 60% dispersion in mineral oil, 283.79 mmol) in dry DMF (20 mL) under N_2 atmosphere. The mixture was left to warm to rt for 30 min. A solution of 1-fluoro-2-nitrobenzene (**S1a**) (20.0 g, 141.74 mmol) in dry DMF (10 mL) was drop-added at 0 °C. The mixture was left to warm to rt for 30 min and then heated to 75 °C for 3 h. After cooling down to rt, 10% $HCl_{(aq)}$ (80 mL) was added to acidify the mixture, which was then extracted with EtOAc (3 × 80 mL). Combined organic layers were washed with saturated NaCl solution (2 × 100 mL), dried over Na_2SO_4 and concentrated under reduced pressure. The oily residue contained ethyl 2-cyano-2-(2-nitrophenyl)acetate (**S2a**), as well as the excessive ethyl cyanoacetate and was used in the next step without further purification; ESI-MS: (*m*/*z*) 257.0 $[M + Na]^+$, 232.9 $[M - H]^-$; HPLC method B: t_r = 3.399 min.

The crude material obtained from the first step was reacted with zinc powder (111.2 g, 1.70 mol) in glacial AcOH (50 mL) according to general procedure H (reaction time 2 h, after complete addition of zinc). The precipitate obtained during the aqueous work-up was not filtered off, but extracted repeatedly with EtOAc. Combined organic layers were dried over Na_2SO_4 and evaporated to dryness. 26.7 g of the crude product were yielded (92% crude yield over two steps) and used in the next step without further purification; HPLC method B: t_r = 5.176 min.

Ethyl 2-amino-6-fluoro-1*H*-indole-3-carboxylate (**S3b**)

The title compound was prepared by a two-step procedure. In the first step ethyl cyanoacetate (6.0 g, 52.80 mmol) was drop-added to an ice-cooled stirring suspension of NaH (2.1 g of a 60% dispersion in mineral oil, 52.80 mmol) in dry DMF (4 mL) under N_2 atmosphere. The mixture was left to warm to rt for 30 min. A solution of 1,4-difluoro-2-nitrobenzene (**S1b**) (4.0 g, 25.14 mmol) in dry DMF (4 mL) was drop-added and the mixture stirred at rt for 30 min and then heated to 75 °C for another 30 min. After cooling down to rt, 10% $HCl_{(aq)}$ (20 mL) was added to acidify the mixture, which was then extracted with EtOAc (3 × 20 mL). Combined organic layers were washed with saturated NaCl solution (2 × 40 mL), dried over Na_2SO_4 and concentrated under reduced pressure. The oily residue contained ethyl 2-cyano-2-(4-fluoro-2-nitrophenyl)acetate (**S2b**), as well as excessive ethyl cyanoacetate and was used in the next step without further purification.

The crude material obtained from the first step was reacted with zinc powder (19.7 g, 301.31 mmol) in glacial AcOH (50 mL) according to general procedure H (reaction time 1.5 h). 4.6 g of the crude product were yielded (82% crude yield over two steps) and used in the next step without further purification; ^1H NMR (300 MHz, DMSO-d_6) δ 10.70 (s, 1H), 7.48 (dd, *J* = 8.5, 5.6 Hz, 1H), 6.94 (dd, *J* = 9.7, 2.4 Hz, 1H), 6.78 (ddd, *J* = 10.2, 8.5, 2.5 Hz, 1H), 6.71 (s, 2H), 4.22 (q, *J* = 7.1 Hz, 2H), 1.31 (t, *J* = 7.1 Hz, 3H).

Ethyl 2-amino-6-bromo-1*H*-indole-3-carboxylate (**S3d**)

The title compound was prepared by a two-step procedure. In the first step ethyl cyanoacetate (8.1 g, 71.59 mmol) was drop-added to an ice-cooled stirring suspension of NaH (2.9 g of a 60% dispersion in mineral oil, 71.59 mmol) in dry DMF (15 mL) under N_2 atmosphere. The dropping funnel was purged with additional dry DMF (3 mL) and the mixture left to warm to rt for 10 min. A solution of 4-bromo-1-fluoro-2-nitrobenzene (**S1d**) (7.5 g, 34.01 mmol) in dry DMF (7.5 mL) was then drop-added to the stirring suspension. Again, the dropping funnel was purged with additional dry DMF (5 mL). The mixture was stirred at rt for 45 min. 10% $HCl_{(aq)}$ (40 mL) was added to acidify the mixture, which was then extracted with EtOAc (3 × 30 mL). Combined organic layers were washed

with saturated NaCl solution (5 × 30 mL), dried over Na_2SO_4 and concentrated under reduced pressure. The liquid residue was treated with ice-cold water resulting in a precipitate, which was filtered off and dried over P_2O_5 in vacuo. 10.7 g of crude ethyl 2-(4-bromo-2-nitrophenyl)-2-cyanoacetate (**S2d**) as a yellow solid (100% crude yield) were yielded and used in the next step without further purification. ^{1}H NMR (300 MHz, DMSO-d_6) δ 8.44 (d, *J* = 2.0 Hz, 1H), 8.13 (dd, *J* = 8.3, 2.1 Hz, 1H), 7.70 (d, *J* = 8.3 Hz, 1H), 6.25 (s, 1H), 4.22 (q, *J* = 7.1 Hz, 2H), 1.19 (t, *J* = 7.1 Hz, 3H); ^{13}C NMR (75 MHz, DMSO-d_6) δ 163.7, 147.4, 137.9, 134.7, 128.7, 124.9, 123.3, 115.0, 63.1, 40.8, 13.8; ESI-MS: (*m*/*z*) 310.8 [M − H]$^-$; HPLC method A: t_r = 6.985 min.

The crude material obtained from the first step (10.3 g, 32.81 mmol) was reacted with zinc powder (12.9 g, 196.83 mmol) in glacial AcOH (80 mL) according to general procedure H (reaction time 2.5 h). 9.4 g of the crude product as a grey-red solid were yielded (98% crude yield over two steps) and used in the next step without further purification.

Ethyl 2-amino-6-iodo-1*H*-indole-3-carboxylate (**S3e**)

The title compound was prepared by a two-step procedure. In the first step a solution of ethyl cyanoacetate (4.2 g, 36.97 mmol) in dry DMF (4 mL) was drop-added to an ice-cooled stirring suspension of NaH (1.48 g of a 60% dispersion in mineral oil, 36.97 mmol) in dry DMF (13 mL) under N_2 atmosphere. The dropping funnel was purged with additional dry DMF (2 mL) and the mixture left to warm to rt. A solution of 1-fluoro-4-iodo-2-nitrobenzene (**S1e**) (4.7 g, 17.60 mmol) in dry DMF (7 mL) was then drop-added to the stirring suspension during 15 min. The mixture was left stirring for 30 min at rt. 10% $HCl_{(aq)}$ (50 mL) was added to acidify the mixture, which was then extracted with EtOAc (3 × 60 mL). Combined organic layers were washed with saturated NaCl solution (3 × 60 mL), dried over Na_2SO_4 and concentrated under reduced pressure. The oily residue was treated with ice-cold water resulting in a yellow precipitate, which was filtered off and dried over P_2O_5 in vacuo. 6.5 g of crude ethyl 2-cyano-2-(4-iodo-2-nitrophenyl)acetate (**S2e**) as a yellow solid were yielded (>100% crude yield) and used in the next step without further purification; ^{1}H NMR (300 MHz, DMSO-d_6) δ 8.53 (d, *J* = 1.7 Hz, 1H), 8.27 (dd, *J* = 8.1, 1.7 Hz, 1H), 7.50 (d, *J* = 8.1 Hz, 1H), 6.21 (s, 1H), 4.22 (q, *J* = 7.1 Hz, 2H), 1.19 (t, *J* = 7.1 Hz, 3H); ^{13}C NMR (75 MHz, DMSO-d_6) δ 163.7, 147.1, 143.7, 134.5, 134.0, 125.1, 115.0, 96.5, 63.1, 40.9, 13.8. ESI-MS: (*m*/*z*) 359.2 [M − H]$^-$; HPLC method A: t_r = 6.970 min.

The crude material obtained from the first step (3.3 g, 9.08 mmol) was dissolved in glacial acetic acid (22 mL). Pronounced dehalogenation was observed when applying the general procedure H, therefore zinc powder (3.3 g, 49.94 mmol) was added in portions at rt and the mixture was then stirred at 80 °C for 30 min. The suspension was left to cool down to rt and filtered rinsing the residue with EtOAc. The filtrate was concentrated under reduced pressure to leave a residue of AcOH, which was neutralized by addition of saturated $NaHCO_3$ solution. The resulting precipitate was filtered off and washed with demineralised water. It was then redissolved in EtOAc and the solution was dried over Na_2SO_4 and evaporated to dryness. 2.5 g of the crude product as a black foam were yielded (85% crude yield over two steps) and used in the next step without further purification.

Ethyl 2-amino-6-methoxy-1*H*-indole-3-carboxylate (**S3f**)

The title compound was prepared by a two-step procedure. In the first step ethyl cyanoacetate (6.3 g, 55.98 mmol) was drop-added to an ice-cooled stirring suspension of NaH (2.2 g, 55.98 mmol) in dry DMF (35 mL) under N_2 atmosphere. The mixture was left to warm to rt for 30 min. A solution of 1-chloro-4-methoxy-2-nitrobenzene (**S1f**) (5.0 g, 26.66 mmol) in dry DMF (7 mL) was drop-added and the mixture stirred at rt for 30 min and then heated to 75 °C for 5 h. After cooling down to rt, 5N $HCl_{(aq)}$ was added to acidify the mixture followed by DCM (150 mL) and saturated NaCl solution (100 mL). The mixture was stirred at rt for 30 min and phases were separated. The aqueous layer was extracted with DCM (50 mL). Combined organic layers were dried over Na_2SO_4 and concentrated under reduced pressure. Purification of the residue by flash column chromatography (SiO_2, hexane:EtOAc gradient elution from 1:0 to 3:2) gave 4.3 g of ethyl 2-cyano-2-(4-methoxy-2-nitrophenyl)acetate (**S2f**); ^{1}H NMR (300 MHz, DMSO-d_6) δ 7.76 (d, *J* = 2.7 Hz, 1H), 7.65 (d, *J* = 8.7 Hz,

1H), 7.46 (dd, *J* = 8.6, 2.8 Hz, 1H), 6.13 (s, 1H), 4.21 (q, *J* = 7.0 Hz, 2H), 1.19 (t, *J* = 7.1 Hz, 3H); ESI-MS: (*m*/*z*) 287.3 $[M + Na]^+$, 263.1 $[M − H]^-$; HPLC method B: t_r = 4.414 min.

The purified material from the first step (4.3 g, 16.27 mmol) was reacted with zinc powder (12.8 g, 195.8 mmol) in glacial AcOH (50 mL) according to general procedure H (reaction time 1.5 h). Purification by flash column chromatography (SiO_2, hexane:EtOAc gradient elution from 1:0 to 3:2) gave 1.6 g of the title compound (26% yield over two steps); ^{1}H NMR (300 MHz, DMSO-d_6) δ 10.49 (s, 1H), 7.40 (d, *J* = 8.4 Hz, 1H), 6.73 (d, *J* = 2.3 Hz, 1H), 6.62–6.46 (m, 3H), 4.20 (q, *J* = 7.1 Hz, 2H), 1.30 (t, *J* = 7.1 Hz, 3H); ESI-MS: (*m*/*z*) 234.3 $[M + H]^+$, 257.3 $[M + Na]^+$, 233.1 $[M − H]^-$; HPLC method B: t_r = 4.805 min.

Ethyl 2-amino-6-(trifluoromethyl)-1*H*-indole-3-carboxylate (**S3g**)

The title compound was prepared by a two-step procedure. In the first step ethyl cyanoacetate (5.3 g, 46.55 mmol) was drop-added to an ice-cooled stirring suspension of NaH (1.9 g of a 60% dispersion in mineral oil, 46.55 mmol) in dry DMF (30 mL) under N_2 atmosphere. The mixture was left to warm to rt for 30 min. A solution of 1-chloro-2-nitro-4-(trifluoromethyl)benzene (**S1g**) (5.0 g, 22.17 mmol) in dry DMF (10 mL) was drop-added and the mixture stirred at rt for 30 min and then heated to 70 °C for 2 h. After cooling down to rt, 10% $HCl_{(aq)}$ was added to acidify the mixture, which was then extracted with EtOAc (3 × 50 mL). Combined organic layers were dried over Na_2SO_4 and concentrated under reduced pressure. The oily residue contained ethyl 2-cyano-2-(2-nitro-4-(trifluoromethyl)phenyl)acetate (**S2g**), as well as excessive ethyl cyanoacetate and was used in the next step without further purification; ESI-MS: (*m*/*z*) 301.1 $[M − H]^-$; HPLC method B: 6.395 min.

The crude material obtained from the first step was reacted with zinc powder (17.4 g, 266.04 mmol) in glacial AcOH (50 mL) according to general procedure G (reaction time 1.5 h). 6.8 g of the crude product were yielded (>100% crude yield over two steps) and used in the next step without further purification; ^{1}H NMR (300 MHz, DMSO-d_6) δ 10.91 (s, 1H), 7.67 (d, *J* = 8.2 Hz, 1H), 7.42 (d, *J* = 1.0 Hz, 1H), 7.26 (dd, *J* = 8.2, 1.0 Hz, 1H), 6.97 (br s, 2H), 4.24 (q, *J* = 7.1 Hz, 2H), 1.32 (t, *J* = 7.1 Hz, 3H); ESI-MS: (*m*/*z*) 270.9 $[M − H]^-$; HPLC method B: t_r = 8.259 min.

Ethyl 2-amino-5-chloro-1*H*-indole-3-carboxylate (**S3h**)

The title compound was prepared by a two-step procedure. In the first step a solution of ethyl cyanoacetate (6.2 g, 54.69 mmol) in dry THF (35 mL) was drop-added to an ice-cooled stirring suspension of K*t*BuO (6.1 g, 54.69 mmol) in dry THF (115 mL) under N_2 atmosphere. The mixture was left to warm to rt and a solution of 2,4-dichloro-1-nitrobenzene (**S1h**) (5.0 g, 26.04 mmol) in dry THF (30 mL) was drop-added. The mixture was stirred at 60 °C for 20 h. After cooling down to rt, 10% $HCl_{(aq)}$ (50 mL) was added and the mixture extracted with EtOAc (3 × 30 mL). Combined organic layers were dried over Na_2SO_4 and concentrated under reduced pressure. The oily residue contained ethyl 2-(5-chloro-2-nitrophenyl)-2-cyanoacetate (**S2h**), as well as excessive ethyl cyanoacetate and was used in the next step without further purification. Purification of a small portion for analytical purposes was performed by flash column chromatography (SiO_2; petroleum ether:EtOAc gradient elution from 4:1 to 3:1); ^{1}H NMR (300 MHz, DMSO-d_6) δ 8.36–8.25 (m, 1H), 7.92–7.79 (m, 2H), 6.18 (s, 1H), 4.22 (q, *J* = 7.0 Hz, 2H), 1.19 (t, *J* = 7.1 Hz, 3H); ^{13}C NMR (75 MHz, DMSO-d_6) δ 163.7, 145.5, 139.8, 133.2, 131.3, 128.2, 127.8, 115.0, 63.3, 41.1, 13.9. ESI-MS: (*m*/*z*) 266.8 $[M − H]^-$; HPLC method A: t_r = 6.130 min.

The crude material obtained from the first step was reacted with zinc powder (10.0 g, 152.32 mmol) in glacial AcOH (60 mL) according to general procedure G (reaction time 2 h). 7.0 g of the crude product as a dark red solid were yielded (>100% crude yield over two steps) and used in the next step without further purification.

Ethyl 2-amino-5-bromo-1*H*-indole-3-carboxylate (**S3i**)

The title compound was prepared by a two-step procedure. In the first step a solution of ethyl cyanoacetate (5.4 g, 47.73 mmol) in dry THF (30 mL) was drop-added to an ice-cooled stirring suspension of K*t*BuO (5.4 g, 47.73 mmol) in dry THF (100 mL) under N_2 atmosphere. The dropping funnel was purged with additional dry THF and the mixture left to warm to rt. A solution of 4-bromo-2-fluoro-1-nitrobenzene (**S1i**) (5.0 g, 22.73 mmol) in dry THF was then drop-added to the stirring suspension. The mixture was heated to reflux for 1.5 h. After cooling down to rt, 10% $HCl_{(aq)}$ (40 mL) was added followed by EtOAc (40 mL). Phases were separated and the aqueous layer was extracted with EtOAc (2 × 30 mL). Combined organic layers were dried over Na_2SO_4 and concentrated under reduced pressure. The oily residue contained ethyl 2-(5-bromo-2-nitrophenyl)-2-cyanoacetate (**S2i**), as well as the excessive ethyl cyanoacetate and was used in the next step without further purification. Purification of a small portion for analytical purposes was performed by flash column chromatography (SiO_2, petroleum ether:(EtOAc + MeOH 95+5) 3:1); ^{1}H NMR (300 MHz, DMSO-d_6) δ 8.25–8.17 (m, 1H), 8.05–7.96 (m, 2H), 6.19 (s, 1H), 4.23 (q, *J* = 7.1 Hz, 2H), 1.19 (t, *J* = 7.1 Hz, 3H); ^{13}C NMR (75 MHz, DMSO-d_6) δ 163.6, 145.8, 136.0, 134.2, 128.7, 128.0, 127.7, 114.9, 63.2, 40.9, 13.8; ESI-MS: (*m*/*z*) 311.0 $[M - H]^-$; HPLC method A: t_r = 6.437 min.

The crude material obtained from the first step was reacted with zinc powder (8.9 g, 136.38 mmol) in glacial AcOH (55 mL) according to general procedure H. Heat was applied after the complete addition of the zinc powder. After a reaction time of 2 h HPLC indicated incomplete conversion, therefore additional zinc powder was added (1.5 g, 22.73 mmol) and stirring continued for 1.5 h. 7.1 g of the crude product were yielded (>100% crude yield over two steps) and used in the next step without further purification; HPLC method A: t_r = 8.042 min.

Ethyl 2-amino-5-methoxy-1*H*-indole-3-carboxylate (**S3j**)

The title compound was prepared by a two-step procedure. In the first step a solution of ethyl cyanoacetate (6.9 g, 61.32 mmol) in dry THF (40 mL) was drop-added to an ice-cooled stirring suspension of K*t*BuO (6.87 g, 61.32 mmol) in dry THF (120 mL) under N_2 atmosphere. The mixture was left to warm to rt and a solution of 2-fluoro-4-methoxy-1-nitrobenzene (**S1j**) (5.0 g, 29.20 mmol) in dry THF (10 mL) was then drop-added to the stirring suspension. The mixture was heated to reflux for 5 h. After cooling down to rt, 10% $HCl_{(aq)}$ (50 mL) was added and the mixture extracted with EtOAc (2 × 30 mL). Combined organic layers were dried over Na_2SO_4 and concentrated under reduced pressure. The oily residue contained ethyl 2-cyano-2-(5-methoxy-2-nitrophenyl)acetate (**S2j**), as well as the excessive ethyl cyanoacetate and was used in the next step without further purification. Purification of a small portion for analytical purposes was performed by flash column chromatography (SiO_2, petroleum ether:EtOAc gradient elution from 3:1 to 3:2); ^{1}H NMR (200 MHz, DMSO-d_6) δ 8.39–8.29 (m, 1H), 7.34–7.22 (m, 2H), 6.14 (s, 1H), 4.21 (qd, *J* = 7.0, 0.8 Hz, 2H), 3.93 (s, 3H), 1.19 (t, *J* = 7.1 Hz, 3H); ^{13}C NMR (50 MHz, DMSO-d_6) δ 163.9, 139.2, 129.0, 128.7, 119.4, 115.3, 115.0, 62.8, 56.5, 41.8, 13.8; ESI-MS: (*m*/*z*) 286.9 $[M + Na]^+$, 262.9 $[M - H]^-$; HPLC method A: t_r = 6.002 min.

The crude material obtained from the first step was reacted with zinc powder (11.5 g, 175.0 mmol) in glacial AcOH (66 mL) according to general procedure H (reaction time 3 h). 5.6 g of the crude product as a red-brown solid were yielded (82% crude yield over two steps) and used in the next step without further purification. Purification of a small portion for analytical purposes was performed by flash column chromatography (SiO_2, DCM:MeOH 97:3); ^{1}H NMR (200 MHz, DMSO-d_6) δ 10.45 (s, 1H), 7.13 (d, *J* = 2.4 Hz, 1H), 6.99 (d, *J* = 8.5 Hz, 1H), 6.60 (br s, 2H), 6.49 (dd, *J* = 8.5, 2.5 Hz, 1H), 4.21 (q, *J* = 7.1 Hz, 2H), 3.71 (s, 3H), 1.32 (t, *J* = 7.1 Hz, 3H); ^{13}C NMR (50 MHz, DMSO-d_6) δ 165.8, 154.5, 153.9, 127.7, 127.3, 110.0, 106.4, 103.0, 84.0, 58.0, 55.2, 14.7; ESI-MS: (*m*/*z*) 233.2 $[M - H]^-$; HPLC method A: t_r = 5.545 min.

Ethyl 2-amino-4-chloro-1*H*-indole-3-carboxylate (**S3k**)

The title compound was prepared by a two-step procedure. In the first step a solution of ethyl cyanoacetate (8.6 g, 76.56 mmol) in dry THF (48 mL) was drop-added to an ice-cooled stirring suspension of K*t*BuO (8.6 g, 76.56 mmol) in dry THF (160 mL). The stirring suspension was left to warm to rt and a solution of 1,2-dichloro-3-nitrobenzene (**S1k**) (7.0 g, 36.46 mmol) in dry THF (44 mL) was drop-added. The mixture was stirred at 60 °C for 43 h, when additional K*t*BuO (1.6 g, 14.58 mmol) was added and stirring at 60 °C continued for another 34 h. After cooling down to rt, 10% $HCl_{(aq)}$ (70 mL) was added and the mixture extracted with EtOAc (3 × 30 mL). Combined organic layers were dried over Na_2SO_4 and concentrated under reduced pressure. The oily residue contained ethyl 2-(2-chloro-6-nitrophenyl)-2-cyanoacetate (**S2k**), as well as excessive ethyl cyanoacetate and was used in the next step without further purification. Purification of a small portion for analytical purposes was performed by flash column chromatography (SiO_2, petroleum ether:EtOAc gradient elution from 4:1 to 1:1); ^{1}H NMR (300 MHz, DMSO-d_6) δ 8.15 (dd, *J* = 8.2, 1.2 Hz, 1H), 8.06 (dd, *J* = 8.2, 1.2 Hz, 1H), 7.79 (t, *J* = 8.2 Hz, 1H), 6.37 (s, 1H), 4.23 (q, *J* = 6.9 Hz, 2H), 1.21 (t, *J* = 7.1 Hz, 3H), ^{13}C NMR (75 MHz, DMSO-d_6) δ 163.2, 149.7, 136.1, 135.5, 132.0, 124.8, 123.4, 114.3, 63.2, 38.2, 13.7; ESI-MS: (*m*/*z*) 266.9 [M − H]$^-$; HPLC method A: t_r = 5.470 min.

The crude material obtained from the first step was reacted with zinc powder (14.1 g, 215.3 mmol) in glacial AcOH (87 mL) according to general procedure H (reaction time 1.5 h). 7.6 g of the crude product as a brown solid were yielded (87% crude yield over two steps) and used in the next step without further purification; HPLC method A: t_r = 7.689 min.

Detailed Procedures for the Preparation of Intermediates S4a,b and S4d–l.

3,9-Dihydro-4*H*-pyrimido[4,5-*b*]indol-4-one (**S4a**)

S3a (26.7 g, 130.7 mmol) and ammonium formate (9.1 g, 144.3 mmol) were stirred in formamide (140 mL) at 170 °C for 18 h. After cooling down to rt, MeOH (150 mL) was added. The resulting precipitate was filtered off, washed with MeOH and dried under reduced pressure. 7.4 g of the crude product were yielded (30% crude yield) and used in the next step without further purification; ^{1}H NMR (300 MHz, DMSO-d_6) δ 12.31–12.04 (m, 2H), 8.12 (s, 1H), 7.98 (d, *J* = 7.7 Hz, 1H), 7.47 (d, *J* = 8.0 Hz, 1H), 7.36–7.29 (m, 1H), 7.23 (td, *J* = 7.6, 1.1 Hz, 1H); ESI-MS: (*m*/*z*) 183.8 [M − H]$^-$; HPLC method B: t_r = 2.066 min.

7-Fluoro-3,9-dihydro-4*H*-pyrimido[4,5-*b*]indol-4-one (**S4b**)

The title compound was prepared from **S3b** (4.0 g, 18.00 mmol) and ammonium formate (1.3 g, 20.70 mmol) in formamide (40 mL) according to general procedure I (reaction time 28 h). 3.2 g of the crude product were yielded (86% crude yield) and used in the next step without further purification; ESI-MS: (*m*/*z*) 201.9 [M − H]$^-$.

7-Bromo-3,9-dihydro-4*H*-pyrimido[4,5-*b*]indol-4-one (**S4d**)

The title compound was prepared from **S3d** (9.0 g, 31.79 mmol) and ammonium formate (2.3 g, 36.56 mmol) in formamide (65 mL) according to general procedure I (reaction time 22 h). 8.7 g of the crude product as a brown solid were yielded (>100% crude yield) and used in the next step without further purification; ESI-MS: (*m*/*z*) 261.8 [M − H]$^-$; HPLC method B: t_r = 4.027 min.

7-Iodo-3,9-dihydro-4H-pyrimido[4,5-b]indol-4-one (**S4e**)

The title compound was prepared from **S3e** (2.5 g, 6.83 mmol) in formamide (17 mL) according to general procedure I (reaction time 29 h). No ammonium formate was used for this reaction. 1.7 g of the crude product as a brown solid were yielded (73% crude yield) and used in the next step without further purification. Purification of a small portion for analytical purposes was performed by flash column chromatography (SiO_2, DCM:MeOH 9:1); 1H NMR (200 MHz, DMSO-d_6) δ 12.37–12.16 (m, 2H), 8.16 (s, 1H), 7.84–7.73 (m, 2H), 7.58–7.49 (m, 1H); 13C NMR (50 MHz, DMSO-d6) δ 158.1, 153.8, 148.2, 136.7, 129.6, 122.4, 121.5, 120.0, 100.1, 88.3; ESI-MS: (*m*/*z*) 310.2 $[M - H]^-$; HPLC method A: t_r = 5.770 min.

7-Methoxy-3,9-dihydro-4*H*-pyrimido[4,5-*b*]indol-4-one (**S4f**)

The title compound was prepared from **S3f** (1.6 g, 6.83 mmol) in formamide (40 mL) according to general procedure I (reaction time 16 h). No ammonium formate was used for this reaction. 1.5 g of the crude product were yielded (97% crude yield) and used in the next step without further purification; ^{1}H NMR (300 MHz, DMSO-d_6) δ 12.15 (s, 1H), 12.04 (s, 1H), 8.05 (s, 1H), 7.84 (d, *J* = 8.6 Hz, 1H), 6.96 (d, *J* = 2.2 Hz, 1H), 6.86 (dd, *J* = 8.6, 2.3 Hz, 1H), 3.81 (s, 3H); ESI-MS: (*m*/*z*) 213.9 $[M - H]^-$.

7-(Trifluoromethyl)-3,9-dihydro-4*H*-pyrimido[4,5-*b*]indol-4-one (**S4g**)

A suspension of **S3g** (4.5 g, 16.53 mmol) in formamide (75 mL) was stirred at 190 °C for 3 h. After cooling down to rt, MeOH (700 mL) was added and stirring continued at rt overnight. The mixture was filtered rinsing with fresh MeOH. The filtrate was concentrated under reduced pressure to the half and the resulting precipitate filtered off. The filtrate was then concentrated under reduced pressure to afford a dark brown oil. Water (300 mL) was added and the mixture stirred at rt for 1 h resulting in a precipitate, which was filtered off, washed with water and dried over P_2O_5 in vacuo. 1 g of the crude product were yielded (24% crude yield) and used in the next step without further purification; ESI-MS: (*m*/*z*) 251.9 $[M - H]^-$; HPLC method B: t_r = 5.113 min.

6-Chloro-3,9-dihydro-4*H*-pyrimido[4,5-*b*]indol-4-one (**S4h**)

The title compound was immediately converted into **3h** (see the detailed procedure for the preparation of **3h**).

6-Bromo-3,9-dihydro-4*H*-pyrimido[4,5-*b*]indol-4-one (**S4i**)

The title compound was prepared from **S3i** (7.1 g, 25.2 mmol) and ammonium formate (1.6 g, 25.9 mmol) in formamide (65 mL) according to general procedure I (reaction time 24 h). 7.3 g of the crude product as a brown solid were yielded (>100% crude yield) and used in the next step without further purification. Purification of a small portion by flash column chromatography for analytical purposes (SiO_2, petroleum ether:(EtOAc + MeOH 95+5) gradient elution from 3:2 to 1:4); ^{1}H NMR (300 MHz, DMSO-d_6) δ 12.49–12.19 (m, 2H), 8.16 (s, 1H), 8.06 (s, 1H), 7.54–7.36 (m, 2H); ^{13}C NMR (50 MHz, DMSO-d_6) δ 158.1, 154.3, 148.3, 134.2, 126.7, 123.9, 122.5, 113.8, 113.4, 99.6; HPLC method A: t_r = 5.689 min.

6-Methoxy-3,9-dihydro-4*H*-pyrimido[4,5-*b*]indol-4-one (**S4j**)

The title compound was prepared from **S3j** (5.3 g, 22.62 mmol) and ammonium formate (1.6 g, 26.02 mmol) in formamide (46 mL) according to general procedure I. Purification of a small portion for analytical purposes was performed by flash column chromatography (SiO_2, DCM:MeOH 9:1). 3.9 g of the crude product were yielded (80% crude yield) and used in the next step withour further purification; ^{1}H NMR (200 MHz, DMSO-d_6) δ 12.16 (s, 1H), 12.04 (s, 1H), 8.08 (s, 1H), 7.48 (d, *J* = 2.4 Hz, 1H), 7.38 (d, *J* = 8.8 Hz, 1H), 6.95 (dd, *J* = 8.9, 2.6 Hz, 1H), 3.80 (s, 3H); ^{13}C NMR (50 MHz, DMSO-

d$_6$) δ 158.5, 154.7, 153.8, 147.2, 130.0, 122.7, 113.6, 112.5, 102.8, 100.2, 55.4; ESI-MS: (*m*/*z*) 214.1 [M − H]$^-$; HPLC method A: t_r = 3.430 min.

5-Chloro-3,9-dihydro-4*H*-pyrimido[4,5-*b*]indol-4-one (**S4k**)

The title compound was prepared from **S3k** (7.0 g, 29.33 mmol) and ammonium formate (2.4 g, 38.13 mmol) in formamide (60 mL) according to general procedure I (reaction time 24 h). 5.5 g of the crude product as a brown solid were yielded (85% crude yield) and used in the next step without further purification. Purification of a small portion for analytical purposes was performed by flash column chromatography (SiO_2, DCM:MeOH 9:1). ^{1}H NMR (200 MHz, DMSO-*d*$_6$) δ 12.49 (s, 1H), 12.16 (s, 1H), 8.13 (s, 1H), 7.40 (dd, *J* = 7.4, 1.7 Hz, 1H), 7.35–7.18 (m, 2H); ^{13}C NMR (50 MHz, DMSO-*d*$_6$) δ 156.9, 154.3, 148.4, 136.9, 125.7, 125.0, 122.3, 120.7, 110.4, 99.4. ESI-MS: (*m*/*z*) 242.0 [M + Na]$^+$, 218.0 [M − H]$^-$; HPLC method A: t_r = 4.214 min.

7-Chloro-2-methyl-3,9-dihydro-4*H*-pyrimido[4,5-*b*]indol-4-one (**S4l**)

Ethyl 2-amino-6-chloro-1*H*-indole-3-carboxylate (**S3c**) was prepared by a two-step procedure from 1,4-dichloro-2-nitrobenzene (**S1c**) as described previously [5] and purified by flash column chromatography (SiO_2, DCM:MeOH 97.5:2.5). Purified **S3c** (400.0 mg, 1.68 mmol) was dissolved in dry acetonitrile (22 mL). The stirring solution was treated with hydrogen chloride gas at rt for 45 min and then refluxed for 2.5 h. After cooling the mixture overnight the formed precipitate was filtered off, washed with cold acetonitrile and dried under reduced pressure to yield 240 mg of the amidine intermediate. EtOH (50 mL) and 10% $NaOH_{(aq)}$ (5 mL) were added and the suspension refluxed for 3.5 h. After cooling down to rt the mixture was concentrated under reduced pressure. 10% $HCl_{(aq)}$ (5 mL) was added to the aqueous residue. The resulting precipitate was filtered off, washed with demineralised water and dried under reduced pressure. 127 mg of a white solid were yielded (32% crude yield) and used in the next step without further purification; ^{1}H NMR (300 MHz, DMSO-*d*$_6$) δ 12.20 (s, 1H), 12.12 (s, 1H), 7.90 (d, *J* = 8.3 Hz, 1H), 7.44 (d, *J* = 1.7 Hz, 1H), 7.22 (dd, *J* = 8.3, 1.9 Hz, 1H), 2.40 (s, 3H); ESI-MS: (*m*/*z*) 232.0 [M − H]$^-$.

Detailed Procedures for the Preparation of Intermediates 3a–l.

4-Chloro-9*H*-pyrimido[4,5-*b*]indole (**3a**)

The title compound was prepared from **S4a** (2.2 g, 11.61 mmol), DIPEA (2.3 g, 17.40 mmol) in $POCl_3$ (11 mL) and chlorobenzene (24 mL) according to general procedure J (reaction time 20 h at a temperature of 100°C). 1.8 g of the crude product as a brown solid were obtained (76% crude yield) and purified by hot filtration from toluene to yield 524 mg of a dark yellow solid; ^{1}H NMR (400 MHz, DMSO-*d*$_6$) δ 12.76 (s, 1H), 8.78 (s, 1H), 8.28 (d, *J* = 7.9 Hz, 1H), 7.68–7.60 (m, 2H), 7.46–7.40 (m, 1H); ESI-MS: (*m*/*z*) 202.0 [M − H]$^-$; HPLC method A: t_r = 7.570 min.

4-Chloro-7-fluoro-9*H*-pyrimido[4,5-*b*]indole (**3b**)

The title compound was prepared from **S4b** (1.5 g, 7.38 mmol) and DIPEA (1.9 g, 14.77 mmol) according to general procedure J (reaction time 4 h), but using $POCl_3$ (20 mL) as only solvent. 1.6 g of the crude product were obtained (98% crude yield) and purified by hot filtration from toluene in portions; ^{1}H NMR (300 MHz, DMSO-*d*$_6$) δ 12.91 (s, 1H), 8.78 (s, 1H), 8.27 (dd, *J* = 8.7, 5.4 Hz, 1H), 7.42 (dd, *J* = 9.5, 2.3 Hz, 1H), 7.33–7.22 (m, 1H); ^{13}C NMR (50 MHz, DMSO-*d*$_6$) δ 162.1 (d, *J* = 243.7 Hz), 156.5 (d, *J* = 1.2 Hz), 153.6, 150.8 (d, *J* = 1.4 Hz), 139.5 (d, *J* = 13.2 Hz), 124.0 (d, *J* = 10.7 Hz), 114.3 (d, *J* = 1.2 Hz), 110.8, 109.9 (d, *J* = 24.3 Hz), 99.1 (d, *J* = 26.6 Hz); ESI-MS: (*m*/*z*) 219.8 [M − H]$^-$; HPLC method B: t_r = 7.001 min.

4,7-Dichloro-9*H*-pyrimido[4,5-*b*]indole (**3c**)

The title compound was prepared in four steps from 1,4-dichloro-2-nitrobenzene (**S1c**) as described previously [5].

7-Bromo-4-chloro-9*H*-pyrimido[4,5-*b*]indole (**3d**)

The title compound was prepared from **S4d** (8.0 g, 30.3 mmol) and DIPEA (5.9 g, 45.44 mmol) in $POCl_3$ (24 mL) and chlorobenzene (60 mL) according to general procedure J (reaction time 7 h). 6.9 g of the crude product as a brown solid were obtained (81% crude yield) and purified by hot filtration in toluene in portions; ^1H NMR (200 MHz, DMSO-d_6) δ 12.86 (s, 1H), 8.78 (s, 1H), 8.09 (d, *J* = 8.5 Hz, 1H), 7.73 (d, *J* = 1.2 Hz, 1H), 7.51 (dd, *J* = 8.5, 1.5 Hz, 1H); ^{13}C NMR (50 MHz, DMSO-d_6) δ 156.1, 154.3, 151.4, 139.3, 124.6, 123.9, 121.1, 116.8, 114.9, 110.7; ESI-MS: (*m*/*z*) 280.0 [M − H]$^-$; HPLC method A: t_r = 8.804 min.

4-Chloro-7-iodo-9*H*-pyrimido[4,5-*b*]indole (**3e**)

S4e (1.8 g, 5.82 mmol) was suspended in chlorobenzene (12 mL) and $POCl_3$ (5 mL) was added carefully. DIPEA (1.1 g, 8.73 mmol) was added portionwise to the stirring suspension and the mixture then stirred at 80 °C for 7 h. The mixture was stirred at rt overnight and then carefully transferred into stirring demineralised water of rt. The stirring mixture was cooled by ice and neutralized with 50% $NaOH_{(aq)}$. The brown precipitate was filtered off and stirred in hot toluene (600 mL) for 1 h. The hot suspension was filtered rinsing with additional hot toluene. The filtrate was concentrated under reduced pressure resulting in a precipitate. The suspension was cooled by ice and filtered off rinsing with cold toluene. The residue was dried under reduced pressure. 843 mg of a brown solid were obtained (44% yield); ^1H NMR (200 MHz, DMSO-d_6) δ 12.82 (s, 1H), 8.79 (s, 1H), 7.99 (d, *J* = 8.3 Hz, 1H), 7.94 (d, *J* = 1.0 Hz, 1H), 7.70 (dd, *J* = 8.3, 1.5 Hz, 1H); ^{13}C NMR (50 MHz, DMSO-d_6) δ 155.9, 154.5, 151.5, 139.5, 130.4, 124.0, 120.8, 117.2, 110.9, 94.0; ESI-MS: (*m*/*z*) 328.0 [M − H]$^-$; HPLC method A: t_r = 9.375 min.

4-Chloro-7-methoxy-9*H*-pyrimido[4,5-*b*]indole (**3f**)

The title compound was prepared from **S4f** (1.4 g, 5.99 mmol) and DIPEA (1.8 g, 13.70 mmol) according to general procedure J (reaction time 5 h), but using $POCl_3$ (25 mL) as only solvent. 1.3 g of the crude product were obtained (87% crude yield) and purified by hot filtration in toluene; ^1H NMR (300 MHz, DMSO-d_6) δ 12.65 (s, 1H), 8.69 (s, 1H), 8.11 (d, *J* = 8.7 Hz, 1H), 7.08 (d, *J* = 2.2 Hz, 1H), 7.02 (dd, *J* = 8.7, 2.3 Hz, 1H), 3.89 (s, 3H); ESI-MS: (*m*/*z*) 231.9 [M − H]$^-$; HPLC method B: t_r = 6.760 min.

4-Chloro-7-(trifluoromethyl)-9*H*-pyrimido[4,5-*b*]indole (**3g**)

A suspension of **S4g** (1.0 g, 3.95 mmol) in $POCl_3$ (50 mL) was stirred at 80°C for 6 h. After cooling down to rt, the mixture was carefully transferred into stirring water of rt and then neutralized with 50% $NaOH_{aq}$. The precipitate was filtered off, washed with water and dried over P_2O_5 in vacuo. 845 mg of the crude product were obtained (79% crude yield). The crude material was not purified by hot filtration from toluene, but used in the next step without further purification; ^1H NMR (300 MHz, DMSO-d_6) δ 13.13 (s, 1H), 8.85 (s, 1H), 8.39 (d, *J* = 8.3 Hz, 1H), 7.87 (s, 1H), 7.69 (dd, *J* = 8.3, 1.0 Hz, 1H); ESI-MS: (*m*/*z*) 269.9 [M − H]$^-$; HPLC method B: t_r = 9.071 min.

4,6-Dichloro-9*H*-pyrimido[4,5-*b*]indole (**3h**)

The title compound was prepared by a two-step procedure. In the first step crude **S3h** (5.8 g) was reacted with ammonium formate (4.2 g, 66.19 mmol) in formamide (50 mL) according to general procedure I (reaction time 35 h). The solvent was replenished in between, as the reaction had dried out. Drying procedures gave 9.5 g of 6-chloro-3,9-dihydro-4*H*-pyrimido[4,5-*b*]indol-4-one (**S4h**) as a crude brown material with residual formamide. Purification of a small portion for analytical purposes was performed by flash column chromatography (SiO_2, DCM:MeOH 9:1); ^1H NMR (300

MHz, DMSO-d_6) δ 12.49–12.21 (m, 2H), 8.16 (s, 1H), 7.91 (d, *J* = 2.0 Hz, 1H), 7.49 (d, *J* = 8.6 Hz, 1H), 7.34 (dd, *J* = 8.6, 2.1 Hz, 1H); ESI-MS: (*m*/*z*) 218.0 [M − H]⁻; HPLC method A: t_r = 5.251 min. The crude material obtained from the first step (4.4 g) was reacted with DIPEA (3.8 g, 30.05 mmol) and $POCl_3$ (17 mL) in chlorobenzene (40 mL) according to general procedure J (reaction time 24 h). The reaction mixture was additionally stirred at rt overnight. The reaction seized at 85% conversion as calculated by HPLC and was therefore worked up according to the general procedure. 4.4 g of a brown solid were obtained and purified by hot filtration from toluene in portions to yield a total 600 mg of a dark yellow solid (25% yield over 4 steps); ^1H NMR (300 MHz, DMSO-d_6) δ 12.91 (s, 1H), 8.80 (s, 1H), 8.21–8.14 (m, 1H), 7.63 (d, *J* = 1.3 Hz, 2H); ESI-MS: (*m*/*z*) 235.9 [M − H]⁻; HPLC method A: t_r = 8.583 min.

6-Bromo-4-chloro-9*H*-pyrimido[4,5-*b*]indole (**3i**)

The title compound was prepared from **S4i** (4.6 g, 17.42 mmol) and DIPEA (3.4 g, 26.13 mmol) in chlorobenzene (35 mL) and $POCl_3$ (15 mL) according to general procedure J (reaction time 20 h). 3.7 g of the crude product as a brown solid were obtained (75% crude yield) and purified by hot filtration from toluene in portions; ^1H NMR (300 MHz, DMSO-d_6) δ 12.91 (s, 1H), 8.79 (s, 1H), 8.29 (d, *J* = 2.0 Hz, 1H), 7.73 (dd, *J* = 8.7, 2.0 Hz, 1H), 7.61–7.53 (m, 1H); ^{13}C NMR (50 MHz, DMSO-d_6) δ 156.1, 154.5, 151.8, 137.3, 130.9, 124.4, 119.5, 114.4, 113.7, 110.3; ESI-MS: (*m*/*z*) 280.0 [M − H]⁻; HPLC method A: t_r = 8.963 min.

4-Chloro-6-methoxy-9*H*-pyrimido[4,5-*b*]indole (**3j**)

The title compound was prepared from **S4j** (2.0 g, 9.29 mmol) and DIPEA (1.8 g, 13.93 mmol) according to general procedure J, but using $POCl_3$ (17.5 mL) as only solvent. 1.7 g of a brown solid were obtained (80% crude yield) and purified by hot filtration from toluene in portions; ^1H NMR (200 MHz, DMSO-d_6) δ 12.58 (s, 1H), 8.71 (s, 1H), 7.65 (d, *J* = 2.4 Hz, 1H), 7.52 (d, *J* = 8.8 Hz, 1H), 7.22 (dd, *J* = 8.8, 2.5 Hz, 1H), 3.85 (s, 3H); ^{13}C NMR (50 MHz, DMSO-d_6) δ 155.9, 154.7, 153.6, 151.2, 132.9, 118.2, 117.4, 113.1, 111.0, 104.8, 55.6; ESI-MS: (*m*/*z*) 231.8 [M − H]⁻; HPLC method A: t_r = 7.566 min.

4,5-Dichloro-9*H*-pyrimido[4,5-*b*]indole (**3k**)

The title compound was prepared from **S4k** (5.2 g, 23.68 mmol), DIPEA (4.6 g, 35.5 mmol) and $POCl_3$ (20 mL) in chlorobenzene (47 mL) according to general procedure J (reaction time 24 h). The wet crude material obtained was directly purified by hot filtration from toluene to yield 2.4 g (43% yield); ^1H NMR (400 MHz, DMSO-d_6) δ 13.12 (s, 1H), 8.77 (s, 1H), 7.57–7.55 (m, 2H), 7.40 (dd, *J* = 5.1, 3.7 Hz, 1H); ^{13}C NMR (101 MHz, DMSO-d_6) δ 156.2, 153.9, 151.3, 140.2, 129.2, 127.0, 123.6, 115.5, 111.2, 110.2; ESI-MS: (*m*/*z*) 236.0 [M − H]⁻; HPLC method A: t_r = 8.664 min.

4,7-Dichloro-2-methyl-9*H*-pyrimido[4,5-*b*]indole (**3l**)

The title compound was prepared from **S4l** (810.0 mg, 3.51 mmol), DIPEA (680.0 mg, 5.26 mmol) and $POCl_3$ (3 mL) in chlorobenzene (7 mL) according to general procedure J. The reaction mixture was left to stir at rt overnight after heating to 80°C for 6 h. The wet crude material obtained was directly purified by hot filtration in toluene to yield 512 mg of a beige solid (59% yield); ^1H NMR (400 MHz, DMSO-d_6) δ 12.62 (s, 1H), 8.15–8.09 (m, 1H), 7.56 (dd, *J* = 1.9, 0.4 Hz, 1H), 7.37 (dd, *J* = 8.4, 1.9 Hz, 1H), 2.67 (s, 3H); ^{13}C NMR (101 MHz, DMSO-d_6) δ 164.0, 157.1, 151.1, 139.0, 132.1, 123.2, 121.7, 116.7, 111.9, 108.1, 25.5; ESI-MS: (*m*/*z*) 251.8 [M + H]⁺, 249.8 [M − H]⁻; HPLC method A: t_r = 9.012 min.
